# The association between maternal body mass index and child obesity: A systematic review and meta-analysis

**DOI:** 10.1371/journal.pmed.1002817

**Published:** 2019-06-11

**Authors:** Nicola Heslehurst, Rute Vieira, Zainab Akhter, Hayley Bailey, Emma Slack, Lem Ngongalah, Augustina Pemu, Judith Rankin

**Affiliations:** 1 Institute of Health & Society, Newcastle University, Newcastle upon Tyne, United Kingdom; 2 Institute of Applied Health Sciences, University of Aberdeen, Aberdeen, United Kingdom; London School of Hygiene and Tropical Medicine, UNITED KINGDOM

## Abstract

**Background:**

There is a global obesity crisis, particularly among women and disadvantaged populations. Early-life intervention to prevent childhood obesity is a priority for public health, global health, and clinical practice. Understanding the association between childhood obesity and maternal pre-pregnancy weight status would inform policy and practice by allowing one to estimate the potential for offspring health gain through channelling resources into intervention. This systematic review and meta-analysis aimed to examine the dose–response association between maternal body mass index (BMI) and childhood obesity in the offspring.

**Methods and findings:**

Searches in MEDLINE, Child Development & Adolescent Studies, CINAHL, Embase, and PsycInfo were carried out in August 2017 and updated in March 2019. Supplementary searches included hand-searching reference lists, performing citation searching, and contacting authors. Two researchers carried out independent screening, data extraction, and quality assessment. Observational studies published in English and reporting associations between continuous and/or categorical maternal and child BMI or *z*-score were included. Categorical outcomes were child obesity (≥95th percentile, primary outcome), overweight/obesity (≥85th percentile), and overweight (85th to 95th percentile). Linear and nonlinear dose–response meta-analyses were conducted using random effects models. Studies that could not be included in meta-analyses were summarised narratively. Seventy-nine of 41,301 studies identified met the inclusion criteria (*n* = 59 cohorts). Meta-analyses of child obesity included 20 studies (*n* = 88,872); child overweight/obesity, 22 studies (*n* = 181,800); and overweight, 10 studies (*n* = 53,238). Associations were nonlinear and there were significantly increased odds of child obesity with maternal obesity (odds ratio [OR] 3.64, 95% CI 2.68–4.95) and maternal overweight (OR 1.89, 95% CI 1.62–2.19). Significantly increased odds were observed for child overweight/obesity (OR 2.69, 95% CI 2.10–3.46) and for child overweight (OR 1.80, 95% CI 1.25, 2.59) with maternal obesity. A limitation of this research is that the included studies did not always report the data in a format that enabled inclusion in this complex meta-analysis.

**Conclusions:**

This research has identified a 264% increase in the odds of child obesity when mothers have obesity before conception. This study provides substantial evidence for the need to develop interventions that commence prior to conception, to support women of childbearing age with weight management in order to halt intergenerational obesity.

## Abstract 2

Nicola Heslehurst and colleagues highlight the association between childhood obesity and maternal pre-pregnancy weight status.

## Author summary

**Why was this study done?:**

- Obesity is a leading cause of lifelong poor health globally, and is significantly associated with inequalities.
- Capitalising on opportunities for early-life prevention of obesity is a priority for public health, global health, and clinical practice.
- This research aimed to estimate the extent to which mothers’ pre-pregnancy body mass index was associated with the weight status of their children, and hence the extent to which this life course stage may be an opportunity for obesity prevention.

**What did the researchers do and find?:**

- This systematic review included 79 studies from international settings that investigated maternal pre-pregnancy body mass index and childhood weight status.
- Meta-analysis identified that maternal pre-pregnancy obesity was significantly associated with child obesity (OR 3.64, 95% CI 2.68–4.95; *n* = 88,872), child overweight/obesity combined (OR 2.69, 956% CI 2.10–3.46; *n* = 181,800), and child overweight (OR 1.80, 95% CI 1.25–2.59; *n* = 53,238).
- Meta-regression found increasing odds of child obesity with increasing child age.

**What do these findings mean?:**

- Our finding of 264% increased odds of child obesity when mothers have pre-pregnancy obesity suggests that commencing obesity prevention interventions prior to conception would be beneficial.
- This research adds to the substantial evidence base that the causes of obesity involve a complex interplay between in utero and life course exposures, which may unequally affect those with a predisposition to obesity.
- Little attention has been given to the preconception period among obesity prevention interventions to date; attention to this period may help to address the complex early-life inequalities associated with obesity development.

## Introduction

Halting childhood obesity is essential to tackle the global obesity crisis and for the future health of the population. Childhood obesity increases the risk of hypertension, cardiovascular disease, diabetes, reduced lung function, mental health conditions, and obesity in adulthood [1,2]. The prevalence of adult obesity has almost tripled over 40 years, with an estimated 13% of the world’s population having obesity, and with the highest prevalence being among women [2–4]. An estimated 41 million children aged 0–5 years and over 340 million aged 5–19 years have overweight or obesity [2,5]. The alarming increase in extreme obesity [6] demonstrates the limited impact of interventions to date in halting or reversing the obesity trend. Prevalence is particularly increasing in low- and middle-income countries, with almost half of overweight and obesity in children under 5 years occurring in Asia, and with a 50% increase in child overweight and obesity in Africa between 2000 and 2016 [2,3]. The cost of treating obesity and related comorbidities was estimated to be 76% higher than healthcare costs for patients with a recommended body mass index (BMI) in the US [7], further demonstrating the need for preventative action.

Prevention of childhood obesity is a priority for public health, global health, and clinical practice, yet interventions to date have produced disappointing results. A key question that remains unanswered is: When is the best life course stage to intervene? There are multiple published studies reporting associations between maternal pre-pregnancy weight status and offspring BMI, with some conflicting and inconsistent results on the extent to which these factors are associated. Understanding this association would inform public health policy and practice by allowing estimation of the potential for offspring health gain through channelling resources into early-life intervention. This systematic review and meta-analysis aimed to determine the dose–response association between maternal pre-pregnancy BMI and offspring obesity.

## Methods

This study is reported as per the MOOSE Checklist for Meta-analyses of Observational Studies (S1 Text). A 5-stage search strategy was implemented to limit the effect of publication bias, as database searches alone are insufficiently rigorous [8]. (1) The MEDLINE, CINAHL, Embase, Child Development & Adolescent Studies, and PsycInfo databases were searched using keywords and MeSH headings developed by an information scientist (S. Robalino) (S1 Fig). Searches were restricted to human studies published in English. No date restrictions were applied. (2) The reference lists of all studies that met the inclusion criteria and all related systematic reviews were hand searched. (3) Citation searches for all studies that met the inclusion criteria and all related systematic reviews were performed using Google Scholar Citations. (4) Any additional studies identified in stages 2 and 3 that met the inclusion criteria were subject to further reference list and citation searching. Stages 2–4 were repeated until no further new studies were identified. (5) Authors of included studies were contacted for additional data when required for inclusion in the meta-analyses. Database searches were completed in August 2017 and updated in March 2019.

Inclusion criteria were peer-reviewed studies reporting both the exposure variable (maternal pre- or early-pregnancy BMI) and the outcome variable (offspring BMI or *z*-score) among children aged 1–18 years. We did not restrict to continuous or categorical exposure or outcome data. Four combinations of data were reported in the included studies: (1) categorical maternal BMI and continuous child BMI/*z*-score, (2) continuous maternal BMI and continuous child BMI/*z*-score, (3) continuous maternal BMI and categorical child BMI/*z*-score, and (4) categorical maternal BMI and categorical child BMI/*z*-score.

Studies reporting duplicate data from the same cohort were excluded, except when data were reported as different combinations and were included in separate analyses. When multiple studies reported data from the same cohort, 3 authors (NH, ZA, and RV) selected which to include using a priority list based on study characteristics: all data required for meta-analysis present, a greater number of maternal BMI categories, child ages not combined, larger sample size, and adjusted analyses. Data extractions and quality assessments were carried out independently by 2 researchers for each included study (NH, RV, ZA, ES, HB, LN, JR, and AP) using a standardised protocol and the Newcastle–Ottawa scale, which assesses information bias, selection bias, and confounding in cohort studies (S2 Table; S2 Fig).

### Analysis of categorical outcomes

The primary outcome was childhood obesity. For the purposes of this systematic review, we categorised 3 outcome variables using BMI percentiles (or equivalent *z*-score categories): obese (≥95th percentile), overweight or obese (≥85th percentile), and overweight (85th to 95th percentile). If data were reported for the same children at multiple ages, then these were related and could not be included in the same analysis; the decision was made to use the oldest age in the meta-analyses and narratively report the younger ages. Dose–response meta-analyses were conducted to investigate the association between maternal and child BMI. When maternal BMI was reported in a continuous form, the reported study-specific linear trends (odds ratios [ORs]) for continuous BMI were used (assuming linearity). For categorical maternal BMI, the study-specific linear trends were derived using the method by Greenland and Longnecker [9], which requires the ORs, confidence intervals (CIs), and number of cases and participants for at least 2 exposure categories. If the adjusted ORs and CIs were not available, the respective unadjusted parameters were derived from the data. The maternal BMI exposure categories were underweight (BMI < 18.5 kg/m^2^), recommended BMI (18.5–24.9 kg/m^2^), overweight (BMI 25.0–29.9 kg/m^2^), and obese (BMI ≥ 30 kg/m^2^). For each category, the midpoint was calculated as the average of the lower and upper bound, and the respective OR was assigned to each midpoint. As the BMI midpoint was required for these analyses, upper and lower cut-offs were applied to open-ended BMI categories. For underweight, a lower limit of 13.5 kg/m^2^ was applied; the respective midpoint was 17 kg/m^2^. For obese, the midpoint was selected as being 35 kg/m^2^, reflecting that the majority of pregnant women with obesity have class I (BMI 30–34.9 kg/m^2^) or class II (BMI 35–39.9 kg/m^2^) obesity [10]. The summary ORs were calculated using the random effects model by DerSimonian and Laird [11].

A 2-stage, random effects, nonlinear dose–response meta-analysis [12–14] was conducted to assess potential nonlinear associations, using cubic splines regression to model maternal BMI (S1 Text). Studies reporting continuous maternal BMI or only 2 categories were excluded from the nonlinear analyses.

### Analysis of continuous outcomes

A dose–response meta-analysis was used to analyse continuous child BMI and *z*-score outcomes with categorical maternal BMI exposures, using maternal recommended BMI (18.5–24.9 kg/m^2^) as the reference group. As child BMI and *z*-scores are 2 different scales, we computed the standardised mean differences (SMDs) as effect sizes, which were combined using the method described by Crippa and Orsini [15]. This consisted of the estimation of flexible dose–response models within each study considering the covariance of the SMDs. A multivariate random effects model was used to combine the parameters describing the study-specific curves to address heterogeneity across studies.

Publication bias was tested for using Egger’s test [16]. A 2-sided *p*-value < 0.05 was considered statistically significant. Sensitivity analyses were performed by excluding 1 study at a time from each meta-analysis. Meta-regressions were carried out to explore additional factors identified a priori as being potentially important sources of heterogeneity. Heterogeneity among studies was evaluated using the *I*^2^ statistic [17] with a threshold of >75% representing considerable heterogeneity [18]. For those factors identified in the meta-regression as statistically significant sources of heterogeneity, subgroup meta-analyses were performed, and pooled ORs were reported for each group. For continuous variables, a linear prediction model was built, and the association between the OR and the continuous variable was plotted. The statistical analyses were conducted using *dosresmeta* [15] and *metafor* [19] packages for R version 3.4.1. Studies that met the inclusion criteria but did not present data suitable for inclusion in the meta-analyses, studies where duplicate data were reported for the same children at different ages, and studies identified in the updated search were summarised narratively and compared to the meta-analysis results. The systematic review was registered on the PROSPERO database (reference CRD42016035599).

## Results

**Fig 1:**
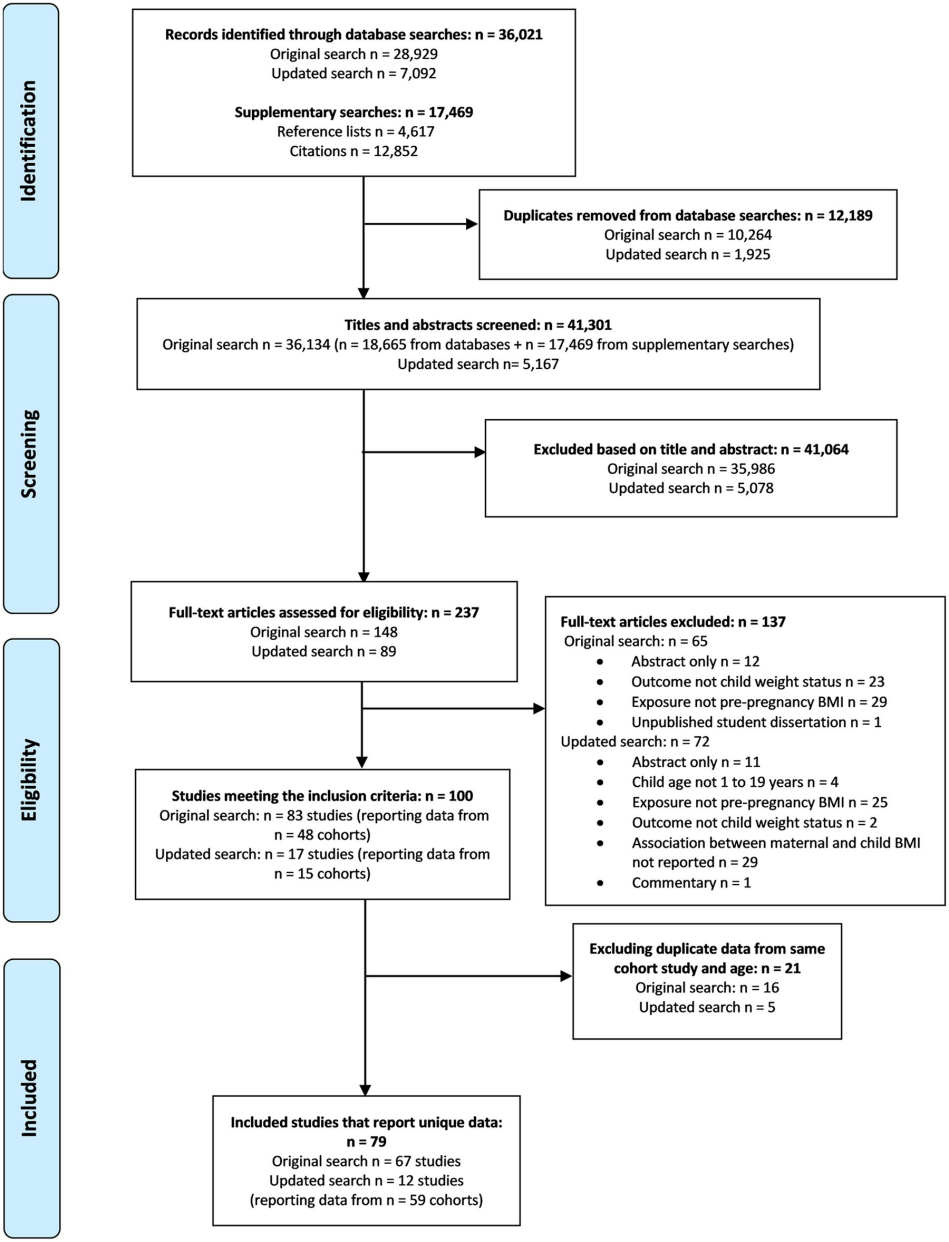
PRISMA flow diagram.

A total of 79 studies reporting data from 59 cohorts are reported in this review (Fig 1). The searches identified 41,301 studies, of which 100 studies met the inclusion criteria (Fig 1; S3 Table). Following exclusion of 21 studies that reported duplicate data (S4 Table), 79 studies remained [20–98], with sample sizes ranging from 70 to 100,612 (S5 Table); 67 studies were identified in the original searches [20,33–98], and a further 12 studies were identified in the updated searches that reported unique data not already included in the review or meta-analysis [21–32] (Fig 1; S5 Table). Of these studies, 56 were prospective, 21 reported national-level data, and the majority (*n* = 63) were published since 2010. Studies were predominantly from the US (*n* = 32), followed by the Netherlands (*n* = 8), UK (*n* = 6), China (*n* = 6), Australia (*n* = 5), Denmark (*n* = 3), Greece (*n* = 3), Norway (*n* = 3), Finland (*n* = 2), Canada (*n* = 2), Malaysia/Singapore (*n* = 2), and Chile, France, Japan, Spain, Sweden, and Sri-Lanka (*n* = 1 each); 1 study included populations from multiple European countries (S5 Table). Of the 9 studies from Asian countries, all except 1 [20] used Asian-specific BMI criteria. The quality score of studies ranged from 3 to 8; no studies were rated low quality, 26 medium quality, and 53 high quality (S6 Table). Additional information was requested from the authors of 56 studies: 8 authors provided additional data, 6 informed us they were unable to provide the data, 41 did not respond, and we were unable to contact the authors of 1 study (S7 Table).

From the original searches, 26 studies reported data for the primary outcome of child obesity (≥95th percentile); 20 of these could be pooled for meta-analysis [33–52]. Twenty-nine studies reported data for childhood overweight or obesity (≥85th percentile); 22 of these reported data that could be pooled for meta-analysis [20,33,34,36,38,40–44,46,52,56–61,70–73]. Fourteen studies reported data for child overweight (85th to 95th percentile); 10 of these could be pooled for meta-analysis [33,34,36,38,40–44,46]. Twenty-three studies reported data for continuous child BMI or *z*-score outcomes, 18 of which could be pooled for meta-analysis [33,36,38,41,70,71,73,76–86]. Some studies reported multiple outcomes and are included in multiple meta-analyses.

### Primary outcome: Child obesity (≥95th percentile)

**Fig 2:**
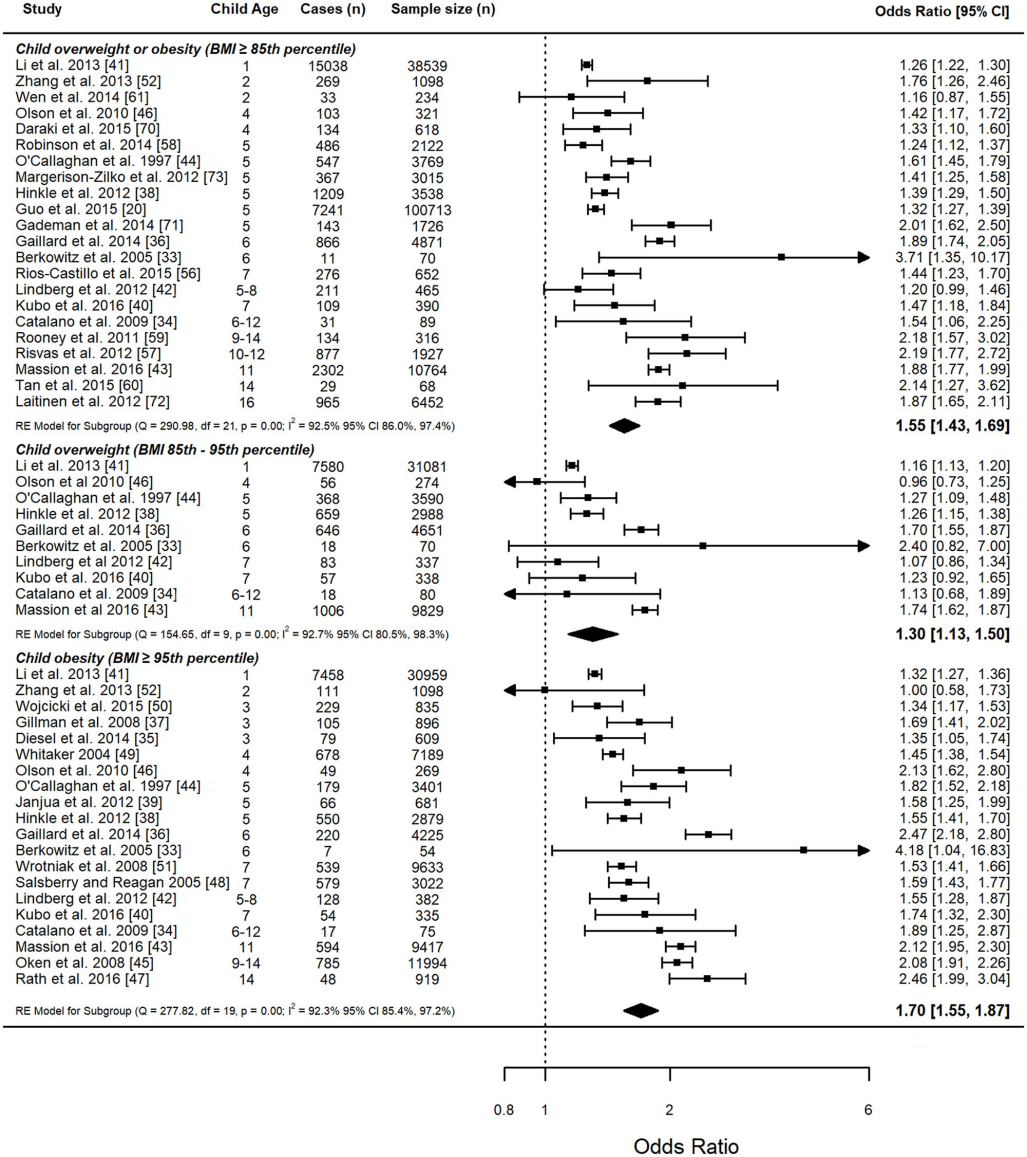
Linear meta-analysis of odds ratios and 95% confidence intervals for child weight status categories. Meta-analysis by child weight status categories: child obesity (≥95th percentile), overweight or obesity (≥85th percentile), and overweight (85th–95th percentile). Pooled summary data for each child weight status category represent the odds ratio and 95% CI for each 5-kg/m^2^ increase in maternal BMI. The size of the data markers indicates the weight assigned to each study in the meta-analysis. Squares represent the odds ratio, bars represent the 95% confidence interval, and diamonds represent the pooled analysis for each child BMI category. RE, random effects.

**Fig 3:**
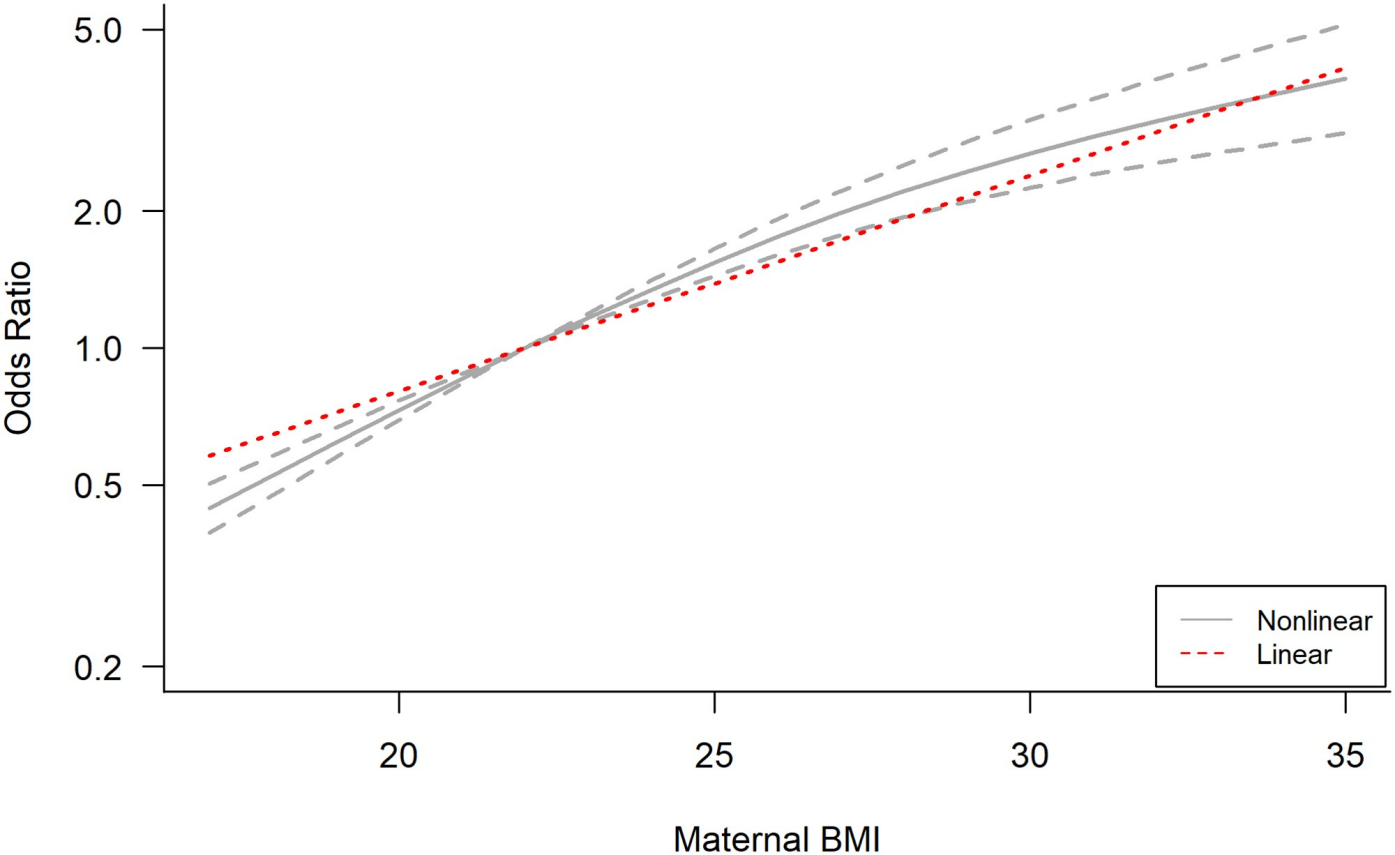
Comparison of linear and nonlinear association between maternal BMI and child obesity (≥95th percentile). Pooled dose–response association between maternal BMI and odds of child obesity. Maternal BMI was modelled with restricted cubic splines in a random effects dose–response model (grey line). Grey dashed lines represent the 95% confidence interval for the spline model. The red dotted line represents the linear trend. The value of 22 kg/m^2^ served as referent. The odds ratios are plotted on the log scale.

The 20 studies with data that could be pooled for meta-analysis included 12,475 cases of obesity among 88,872 children aged between 1 and 14 years. In the linear dose–response meta-analysis, the OR for each 5-kg/m^2^ increase in maternal BMI was 1.70 (95% CI 1.55–1.87) (Fig 2). Linearity of association between maternal BMI and child BMI was rejected (*p <* 0.001; S8 Table), although linear and nonlinear effect size estimates for each maternal BMI category were of a similar magnitude. Assuming a nonlinear association, there was a statistically significant decrease in the odds of child obesity when mothers had an underweight BMI compared with the reference group, and an increase in odds of 89% (OR 1.89, 95% CI 1.62–2.19) with maternal overweight and 264% (OR 3.64, 95% CI 2.68–4.95) with maternal obesity (Table 1; Fig 3). There was no evidence of publication bias in the analyses of children with obesity (*p* = 0.53; S3 Fig).

**Table 1:** Linear and nonlinear dose–response meta-analyses for maternal and child BMI.

Outcome	Model	Maternal underweight (BMI 17 kg/m^2^)^a^	Maternal reference (BMI 22 kg/m^2^)^a^	Maternal overweight (BMI 27 kg/m^2^)^a^	Maternal obesity (BMI 35 kg/m^2^)^a^
Child obesity (BMI ≥ 95th percentile)	Linear OR (95% CI)	0.60 (0.53, 0.67)	1.00	1.68 (1.50, 1.89)	3.68 (2.85, 5.21)
	Nonlinear OR (95% CI)	0.47 (0.39, 0.57)	1.00	1.89 (1.62, 2.19)	3.64 (2.68, 4.95)
Child overweight/obesity (BMI ≥ 85th percentile)	Linear OR (95% CI)	0.65 (0.60, 0.71)	1.00	1.54 (1.41, 1.67)	3.05 (2.45, 3.81)
	Nonlinear OR (95% CI)	0.51 (0.44, 0.60)	1.00	1.65 (1.47, 1.85)	2.69 (2.10, 3.46)
Child overweight (BMI 85th to 95th percentile)	Linear OR (95% CI)	0.77 (0.67, 0.88)	1.00	1.30 (1.13, 1.50)	1.99 (1.39, 2.85)
	Nonlinear OR (95% CI)	0.64 (0.53, 0.78)	1.00	1.41 (1.19, 1.67)	1.80 (1.25, 2.59)
Child continuous BMI and *z*-score	Linear SMD (95% CI)	−0.48 (−0.83, −0.13)	0.00	0.48 (0.13, 0.83)	1.24 (0.33, 2.15)
	Nonlinear SMD (95% CI)	−0.50 (−0.65, −0.35)	0.00	0.45 (0.31, 0.59)	0.99 (0.62, 1.36)

^a^BMI represents the maternal pre-/early-pregnancy BMI category midpoint estimate.

BMI, body mass index; CI, confidence interval; OR, odds ratio; SMD, standardized mean difference.

Additional data were available for child obesity between ages 1 and 13 and were not included in the meta-analysis [23,26,30,47–49,53–55,67–69] (S9 Table). All additional ORs for maternal obesity and child obesity were statistically significant, ranging from 1.37 to 5.58. The majority of additional ORs reported for maternal overweight and child obesity were statistically significant, ranging from 1.04 to 3.36.

### Secondary outcomes

#### Child overweight or obesity (≥85th percentile)

The 22 studies [20,33,34,36,38,40–44,46,52,56–61,70–73] with data available to be pooled for meta-analysis included 31,328 cases of overweight or obesity among 181,800 children aged 1 to 16 years. In the linear dose–response analysis, the OR for each 5-kg/m^2^ increase in maternal BMI was 1.55 (95% CI 1.43–1.69) (Fig 2). There was evidence of a nonlinear association (*p* < 0.001; S8 Table), with a statistically significant decrease in odds of child overweight or obesity when mothers had an underweight BMI compared with the reference group, and an increase in odds of child overweight or obesity of 65% (OR 1.65, 95% CI 1.47–1.85) for maternal overweight and 169% (OR 2.69, 95% CI 2.10–3.46) for maternal obesity (Table 1; S4 Fig). There was no evidence of publication bias in the analyses of overweight or obese children (*p* > 0.05; S3 Fig).

Additional data were available for child overweight or obesity between ages 1 and 14 years and were not included in the meta-analysis [21,22,25,27,29,31,53,59,62–66,69,74,97,98] (S10 Table). All additional ORs were statistically significant for all types of maternal BMI exposure: For maternal obesity, ORs ranged from 1.58 to 4.59; for maternal overweight, ORs ranged from 1.3 to 2.35; and for maternal overweight or obesity (BMI ≥ 25 kg/m^2^), ORs ranged from 1.13 to 4.00. The ORs for continuous maternal BMI and child overweight/obesity ranged between 1.09 and 1.60.

#### Child overweight (BMI 85th to 95th percentile)

The 10 studies [33,34,36,38,40–44,46] with data available to pool for meta-analysis included 10,491 cases of child overweight among 53,238 children aged 1 to 11 years. In the dose–response analysis, the OR for each 5-kg/m^2^ increase in maternal BMI was 1.30 (95% CI 1.13–1.50) (Fig 2). There was evidence of a nonlinear association (*p* < 0.001; S8 Table), with a statistically significant decrease in odds of child overweight for underweight maternal BMI compared with the reference group, and an increase in odds of child overweight of 41% (OR 1.41, 95% CI 1.19–1.67) for maternal overweight and 80% (OR 1.80, 95% CI 1.25–2.59) for maternal obesity (Table 1; S5 Fig). There was no evidence of publication bias in the analyses of child overweight (*p* = 0.71; S3 Fig).

Additional data were available for child overweight for children between ages 4 and 13 years and were not included in the meta-analysis [39,54,69,75] (S11 Table). All reported significantly increased odds of child overweight with maternal obesity, with ORs ranging between 1.26 and 2.29.

#### Continuous child BMI and *z*-score

The 18 studies [33,36,38,41,70,71,73,76–86] with data for meta-analysis of continuous child BMI (*n* = 11 studies) and *z*-score (*n* = 7 studies) outcomes and categorical maternal BMI exposures included data on 90,580 children (*n* = 43,877 for BMI; *n* = 46,703 for BMI *z*-score). Linear meta-analyses for BMI and BMI *z*-score showed a SMD of 0.09 (95% CI 0.01–0.17) for child BMI for every 1-kg/m^2^ increase in maternal BMI, and a SMD of 0.10 (95% CI –0.02 to 0.23) for child BMI *z*-score (S6 Fig). Linearity was not rejected for either measure when analysed separately (S8 Table). However, when analysing the pooled BMI and *z*-score data, linearity was rejected, and the nonlinear analysis showed that the SMD in child BMI was significantly decreased for maternal underweight, and increased for maternal overweight and obesity (Table 1; S7 Fig). There was no evidence of publication bias for the continuous outcomes (*p* = 0.995; S3 Fig).

Additional data were available for associations between categorical maternal BMI and continuous child BMI or *z*-score for children aged 1 to 9 years, but were not included in the meta-analysis [28,32,33,61,76,82,84,87–91] (S12 Table), and for associations between continuous maternal BMI and continuous child BMI or *z*-score for children aged 1 to 18 years [24,36,60,61,71,75,80,85,86,88,92–97] (S13 Table). All except 1 showed a significant association between increasing maternal BMI and increasing child BMI or *z*-score.

### Heterogeneity and sensitivity analyses

Sensitivity analyses for linear and nonlinear meta-analyses did not show any 1 study to be substantially influencing the overall direction of association, effect size, statistical significance, or heterogeneity (S14–S17 Tables). Heterogeneity was present in all analyses (*I*^2^ 92.3%–99.9%; S18 Table). Meta-regression identified child age and continent of study to contribute to heterogeneity for all categorical child BMI outcomes, but no factors substantially reduced heterogeneity between studies for continuous outcomes (S18 Table). Univariate adjustment for child age decreased the *I*^2^ to 81.2% for obesity analyses, 83.2% for overweight/obesity analyses, and 83.0% for overweight analyses; continent of study decreased the *I*^2^ to 76.4%, 80.3%, and 0.08%, respectively (S18 Table). When adjusting for both factors, the overall *I*^2^ decreased to 62.6%, 72.9%, and 25.4%, respectively. Subgroup meta-analysis for continent of study identified that ORs for child obesity and overweight were consistently highest in studies from Europe (S19 Table), and plots show that the predicted average OR for child obesity and overweight increases with increasing child age (S8–S10 Figs).

## Discussion

This systematic review aimed to determine the dose–response association between maternal pre-pregnancy BMI and child obesity. The meta-analyses identified significantly increased odds of child obesity with increasing maternal BMI; this association was strongest with maternal obesity, which increased the odds of child obesity by 264%, followed by maternal overweight, which increased the odds by 89%. Similar patterns were observed for the secondary categorical and continuous child BMI and *z*-score outcomes. Meta-regression found an association between child obesity and increasing child age, which may reflect the combination of in utero and child life course exposures. The development of obesity involves a complex interplay between physiological, environmental, psychological, social, and behavioural exposures [99]. For example, there is evidence of epigenetic processes in utero that contribute to offspring obesity, including alterations in DNA methylation and the gut microbiome [100]. Additional life course exposures include socio-economic status, food production and marketing, food insecurity, and obesogenic environments, which promote unhealthy lifestyles to which some individuals are genetically more susceptible [99,101–103]. If mothers were exposed to these complex factors, contributing to their own obesity development, then their children are also likely to be exposed to the same complex factors, which exacerbate in utero development and predisposition to obesity.

This systematic review has strengths and limitations. The rigorous search strategy involved an experienced information scientist, database searches were supplemented with additional searches, and we contacted authors in an attempt to maximise the number of studies included in the meta-analyses. Procedures to minimise human error and subjectivity included duplicate independent screening, data extraction, and quality assessment. The meta-analyses were complex given the inclusion of both categorical and continuous exposure and outcome data, and the fact that we did not restrict the outcome to either child BMI or *z*-score. The decision was made to include all combinations of data based on pre-existing knowledge of the variability in how data are reported in the published literature; had we restricted the inclusion criteria to facilitate a more straightforward meta-analysis, we would have incurred bias by excluding a well-established body of evidence. However, the complex analytical approach employed required specific data to be reported in the studies, which were not always available, and the efforts to contact authors had limited return on time invested. Future research should ensure that full data are reported in the publications to enable inclusion in more complex meta-analyses. There was substantial heterogeneity between studies. Meta-regression explored maternal and child clinical, socio-demographic, behavioural, and study design factors, yet only child age and the continent of study significantly contributed towards heterogeneity between studies. It must be noted that the meta-regression relied on the primary studies accounting for these factors in their analyses; for example, paternal BMI, gestational weight gain, and gestational diabetes were rarely considered. Future research using individual participant data would be an opportunity to further explore the complex picture of child obesity development to inform targeted interventions. Few studies reported maternal obesity classes; rather, there was a tendency to group all obesity as BMI ≥ 30 kg/m^2^. This resulted in wider confidence intervals and less certainty of the true effect size at the upper ends of maternal BMI. Obesity is not a homogeneous group and in order to better understand the differences within obesity, future research should use obesity classes when defining categories. Disappointingly, we identified limited data from low- and middle-income countries. Our inclusion criteria restricted to studies published in English, and we excluded 1 non-English-language study at the title and abstract stage that appeared to otherwise meet the inclusion criteria [104]. This study from Brazil identified a 10% increase in adolescent obesity per 1-kg/m^2^ increase in maternal pre-pregnancy BMI: OR 1.09 (95% CI 1.01–1.19) for males and OR 1.16 (95% CI 1.04–1.30) for females. These results are similar to our pooled linear meta-analysis result (OR 1.70 per 5-kg/m^2^ increase in maternal BMI). Given the global inequalities associated with childhood obesity, this is an important area for future research.

This research has identified the need for early intervention in the prevention of childhood obesity, starting before conception. For many years, obesity prevention interventions have targeted environmental settings, such as schools [105]. However, increasing obesity prevalence in preschool age children highlights the importance of earlier prevention, in the first 1,000 days of life, from conception to 2 years old. Considering the evidence on developmental origins of health and disease, it could be argued that the first 1,000 days is not early enough. Obesity prevention must start with women of childbearing age [106], and preconception has been identified as a critical life course time period for ending child obesity [107]. Little attention has been given to the preconception period among interventions to date [108]. This attention is essential for future public health and clinical research, policy, and practice, given the inequalities associated with obesity. The failure to implement preventative action increases intergenerational life course inequalities.

This systematic review and meta-analysis identified significantly increased odds of child obesity when mothers have obesity before conception. This study provides substantial evidence for the need to develop interventions commencing preconception, to support women of childbearing age with weight management to contribute to the prevention of childhood obesity. Given the complex interplay between physiological, social, economic, environmental, and behavioural factors in the development of obesity, multifactorial interventions targeting women of childbearing age are likely to be required to halt intergenerational obesity.

## Supporting information

S1 Fig Translation of search terms across databases. (DOCX)

S2 Fig Adapted Newcastle–Ottawa scale for cohort studies. (DOCX)

S3 Fig Tests for publication bias. (DOCX)

S4 Fig Comparison of linear and nonlinear association between maternal BMI and child overweight or obesity (≥85th percentile). (DOCX)

S5 Fig Comparison of linear and nonlinear association between maternal BMI and child overweight (85th to 95th percentile). (DOCX)

S6 Fig Linear meta-analysis of SMDs for all BMI and BMI *z*-score outcomes and association with 5-kg/m^2^ increase in maternal BMI. (DOCX)

S7 Fig Comparison of linear and nonlinear association between maternal BMI and continuous child BMI and *z*-score. (DOCX)

S8 Fig Scatterplot showing the relationship between child age and OR of child obesity (≥95th percentile). (DOCX)

S9 Fig Scatterplot showing the relationship between child age and OR of child overweight or obesity (≥85th percentile). (DOCX)

S10 Fig Scatterplot showing the relationship between child age and OR of child overweight (85th to 95th percentile). (DOCX)

S1 Table MOOSE Checklist for meta-analyses of Observational Studies. (DOCX)

S2 Table Data extraction protocol. (DOCX)

S3 Table Screening: Systematic review reference lists screened, and full papers screened and excluded. (DOCX)

S4 Table Screening: Studies excluded due to duplicate cohort data. (DOCX)

S5 Table Details of included studies as reported in the original papers. (DOCX)

S6 Table Quality scores for all included studies. (DOCX)

S7 Table Contacting authors for additional information. (DOCX)

S8 Table Nonlinear meta-analyses using cubic splines regression. (DOCX)

S9 Table Additional data reported that were not included in meta-analysis for child obesity (≥95th percentile). (DOCX)

S10 Table Additional data reported that were not included in meta-analysis for child overweight or obesity (≥85th percentile). (DOCX)

S11 Table Additional data reported that were not included in meta-analysis for child overweight (85th to 95th percentile). (DOCX)

S12 Table Additional data for narrative overview for continuous child BMI and *z*-score outcomes (categorical maternal BMI exposure). (DOCX)

S13 Table Additional data for narrative overview for continuous child BMI and *z*-score outcomes. (DOCX)

S14 Table Maternal BMI and child overweight/obesity (BMI ≥ 85th percentile) sensitivity analysis. (DOCX)

S15 Table Maternal BMI and child overweight (BMI 85th to 95th percentile) sensitivity analysis. (DOCX)

S16 Table Maternal BMI and child obesity (BMI ≥ 95th percentile) sensitivity analysis. (DOCX)

S17 Table Maternal BMI and continuous child BMI and BMI *z*-score outcomes (mean differences) sensitivity analysis. (DOCX)

S18 Table Results of univariate meta-regression models evaluating the effect of potential sources of heterogeneity. (DOCX)

S19 Table Subgroup meta-analysis: Odds of childhood weight status per 5-kg/m^2^ increase in maternal BMI, according to the continent of study. (DOCX)

S1 Text Dose–response meta-analysis methods. (DOCX)

## Abbreviations

BMI: body mass index
CI: confidence interval
OR: odds ratio
SMD: standardised mean difference
