# Supplementary material for: The association between maternal body mass index and child obesity: A systematic review and meta-analysis
Source: PLoS Med. 2019 Jun 11;16(6):e1002817. doi: 10.1371/journal.pmed.1002817 (PMC6559702; doi:10.1371/journal.pmed.1002817)
Supplement: S1 Fig — (DOCX) [file pmed.1002817.s001.docx]

# S1 Fig: Translation of search terms across databases

| **Ovid Medline 1946 to August 2017; updated March 2019**  1. Pre*pregnancy.mp.  2. Pre*gravid.mp.  3. Maternal.mp.  4. before pregnancy.mp.  5. Pre*conception.mp.  6. Peri*conception.mp.  7. Mothers/  8. Mother*.mp.  9. Pregnancy/  10. Pregnan*.mp.  11. 1 or 2 or 3 or 4 or 5 or 6 or 7 or 8 or 9 or 10  12. body mass index.mp. or Body Mass Index/  13. BMI.mp.  14. Thinness.mp. or Thinness/  15. Underweight.mp.  16. normal weight.mp.  17. Overweight.mp. or Overweight/  18. Obesity/  19. Obes*.mp.  20. body weight.mp. or Body Weight/  21. nutritional status.mp. or Nutritional Status/  22. 12 or 13 or 14 or 15 or 16 or 17 or 18 or 19 or 20 or 21  23. 11 and 22  24. Offspring.mp.  25. Child/  26. Child*.mp.  27. Infant*.mp. or Infant/  28. Infant, Newborn/ or newborn*.mp.  29. Toddler*.mp.  30. Child, Preschool/ or pre*school*.mp.  31. Adolescent/ or adolescen*.mp.  32. Teen*.mp.  33. school age.mp.  34. Kindergar*en.mp.  35. 24 or 25 or 26 or 27 or 28 or 29 or 30 or 31 or 32 or 33 or 34  36. p*ediatric obesity.mp. or Pediatric Obesity/  37. body composition.mp. or Body Composition/  38. 22 or 36 or 37  39. 35 and 38  40. Observational Study/ or Observational Studies as Topic/ or observational.mp.  41. Epidemiologic Research Design/ or Epidemiologic Methods/ or Epidemiologic Studies/  42. epidemiolog* stud*.mp.  43. Cohort Studies/ or cohort.mp.  44. Prospective Studies/ or prospective.mp.  45. Retrospective Studies/ or retrospective.mp.  46. Case-Control Studies/ or case*control.mp.  47. follow*up.mp. or Follow-Up Studies/  48. longitudinal.mp. or Longitudinal Studies/  49. Cross-Sectional Studies/ or cross*section*.mp.  50. anthropometry.mp. or Anthropometry/  51. 40 or 41 or 42 or 43 or 44 or 45 or 46 or 47 or 48 or 49 or 50  52. 23 and 39  53. 51 and 52  54. limit 53 to (english language and humans)  **Ovid Embase 1974 to August 2017; updated March 2019**  1. pre*pregnancy.mp.  2. pre*gravid.mp.  3. maternal.mp.  4. before pregnancy.mp.  5. pre*conception.mp.  6. peri*conception.mp.  7. mother/ or mother*.mp.  8. pregnancy/  9. pregnan*.mp.  10. 1 or 2 or 3 or 4 or 5 or 6 or 7 or 8 or 9  11. body mass index.mp. or body mass/  12. BMI.mp.  13. thinness.mp.  14. underweight.mp. or underweight/  15. normal weight.mp.  16. overweight.mp.  17. obesity/  18. obes*.mp.  19. body weight.mp. or body weight/  20. nutritional status.mp. or nutritional status/  21. maternal obesity.mp. or maternal obesity/  22. 11 or 12 or 13 or 14 or 15 or 16 or 17 or 18 or 19 or 20 or 21  23. 10 and 22  24. offspring.mp. or progeny/  25. child/  26. child*.mp.  27. infant/  28. infant*.mp.  29. newborn/  30. newborn*.mp.  31. toddler/ or toddler*.mp.  32. pre*school*.mp.  33. adolescence/ or adolescen*.mp.  34. teen*.mp.  35. school*age.mp.  36. kindergarten/ or kindergar*en.mp.  37. 24 or 25 or 26 or 27 or 28 or 29 or 30 or 31 or 32 or 33 or 34 or 35 or 36  38. childhood obesity/ or p*ediatric obesity.mp.  39. adolescent obesity.mp. or adolescent obesity/  40. body composition.mp. or body composition/  41. 11 or 12 or 13 or 14 or 15 or 16 or 17 or 18 or 19 or 20 or 38 or 39 or 40  42. 37 and 41  43. observational study/ or observational method/ or observational.mp.  44. epidemiolog* stud*.mp.  45. cohort analysis/ or cohort.mp.  46. prospective study/ or prospective.mp.  47. retrospective study/ or retrospective.mp.  48. case control study/ or case*control.mp.  49. follow-up studies/ or follow*up stud*.mp.  50. longitudinal study/ or longitudinal.mp.  51. cross*section*.mp. or cross-sectional study/  52. anthropometry.mp. or anthropometry/  53. 43 or 44 or 45 or 46 or 47 or 48 or 49 or 50 or 51 or 52  54. 23 and 42  55. 53 and 54  56. limit 55 to (human and english language)  **Ovid PsycINFO 1806 to August 2017; updated March 2019**  1. pre*pregnancy.mp.  2. pre*gravid.mp.  3. maternal.mp.  4. before pregnancy.mp.  5. pre*conception.mp.  6. peri*conception.mp.  7. MOTHERS/  8. mother*.mp.  9. PREGNANCY/  10. pregnan*.mp.  11. 1 or 2 or 3 or 4 or 5 or 6 or 7 or 8 or 9 or 10  12. Body Mass Index/ or body mass index.mp.  13. BMI.mp.  14. thinness.mp.  15. underweight.mp. or UNDERWEIGHT/  16. normal weight.mp.  17. overweight.mp. or OVERWEIGHT/  18. OBESITY/  19. obes*.mp.  20. body weight.mp. or Body Weight/  21. nutritional status.mp.  22. 12 or 13 or 14 or 15 or 16 or 17 or 18 or 19 or 20 or 21  23. 11 and 22  24. offspring.mp. or OFFSPRING/  25. child*.mp.  26. infant*.mp.  27. newborn*.mp.  28. toddler*.mp.  29. PRESCHOOL STUDENTS/ or pre*school.mp.  30. adolescen*.mp.  31. teen*.mp.  32. school*age.mp.  33. kindergar*en.mp.  34. 24 or 25 or 26 or 27 or 28 or 29 or 30 or 31 or 32 or 33  35. p*ediatric obesity.mp.  36. body composition.mp.  37. 22 or 35 or 36  38. 34 and 37  39. exp Observation Methods/ or observational.mp.  40. epidemiolog* stud*.mp.  41. COHORT ANALYSIS/ or cohort.mp.  42. PROSPECTIVE STUDIES/ or prospective.mp.  43. RETROSPECTIVE STUDIES/ or retrospective.mp.  44. case*control.mp.  45. Followup Studies/ or follow*up.mp.  46. LONGITUDINAL STUDIES/ or longitudinal.mp.  47. cross*section*.mp.  48. anthropometry.mp. or ANTHROPOMETRY/  49. 39 or 40 or 41 or 42 or 43 or 44 or 45 or 46 or 47 or 48  50. 23 and 38  51. 49 and 50  52. limit 51 to (human and english language)  **EBSCO CINAHL 1981 to August 2017; updated March 2019**  1. "pre*pregnancy"  2. "pre*gravid"  3. "maternal"  4. "before pregnancy"  5. "pre*conception"  6. "peri*conception"  7. (MH "Mothers")  8. "mother*"  9. (MH "Pregnancy")  10. "pregnan*"  11. S1 OR S2 OR S3 OR S4 OR S5 OR S6 OR S7 OR S8 OR S9 OR S10  12. (MH "Body Mass Index") OR ""body mass index""  13. "BMI"  14. (MH "Thinness") OR "Thinness"  15. "underweight"  16. ""normal weight""  17. "overweight"  18. (MH "Obesity")  19. "obes*"  20. (MH "Body Weight") OR ""body weight""  21. (MH "Nutritional Status") OR ""nutritional status""  22. S12 OR S13 OR S14 OR S15 OR S16 OR S17 OR S18 OR S19 OR S20 OR S21  23. S11 AND S22  24. "offspring"  25. (MH "Child")  26. "child*"  27. "infant*"  28. (MH "Infant, Newborn") OR "newborn*"  29. "toddler*"  30. "pre*school*"  31. (MH "Adolescence") OR "adolescen*"  32. "teen*"  33. ""school*age""  34. "kindergar*en"  35. S24 OR S25 OR S26 OR S27 OR S28 OR S29 OR S30 OR S31 OR S32 OR S33 OR S34  36. (MH "Pediatric Obesity") OR ""p*ediatric obesity""  37. (MH "Body Composition") OR ""body composition""  38. S22 OR S36 OR S37  39. S35 AND S38  40. (MH "Observational Methods") OR "observational"  41. (MH "Epidemiological Research")  42. ""epidemiolog* stud*""  43. "cohort"  44. (MH "Prospective Studies") OR "prospective"  45. (MH "Retrospective Design") OR "retrospective"  46. "case*control"  47. "follow*up"  48. "longitudinal"  49. "cross*section*" OR (MH "Cross Sectional Studies")  50. (MH "Anthropometry") OR "anthropometry"  51. S40 OR S41 OR S42 OR S43 OR S44 OR S45 OR S46 OR S47 OR S48 OR S49 OR S50  52. S23 AND S39  53. S51 AND S52  54. S51 AND S52  **EBSCO Child Development & Adolescent Studies 1927 to August 2017; updated March 2019**  1. pre*pregnancy  2. pre*gravid  3. maternal  4. "before pregnancy"  5. pre*conception  6. peri*conception  7. mother*  8. pregnan*  9. S1 OR S2 OR S3 OR S4 OR S5 OR S6 OR S7 OR S8  10. "body mass index"  11. BMI  12. thinness  13. underweight  14. "normal weight"  15. overweight  16. obes*  17. "body weight"  18. "nutritional status"  19. S10 OR S11 OR S12 OR S13 OR S14 OR S15 OR S16 OR S17 OR S18  20. S9 AND S19  21. offspring  22. child*  23. infant*  24. newborn*  25. toddler*  26. pre*school*  27. adolescen*  28. teen*  29. kindergar*en  30. S21 OR S22 OR S23 OR S24 OR S25 OR S26 OR S27 OR S28 OR S29  31. "p*ediatric obesity"  32. "body composition"  33. S19 OR S31 OR S32  34. S30 AND S33  35. observational  36. "epidemiolog* stud*"  37. cohort  38. prospective  39. retrospective  40. case*control  41. follow*up  42. longitudinal  43. cross*section*  44. anthropometry  45. S35 OR S36 OR S37 OR S38 OR S39 OR S40 OR S41 OR S42 OR S43 OR S44  46. S20 AND S34  47. S45 AND S46 |
| --- |
