# Supplementary material for: The association between maternal body mass index and child obesity: A systematic review and meta-analysis
Source: PLoS Med. 2019 Jun 11;16(6):e1002817. doi: 10.1371/journal.pmed.1002817 (PMC6559702; doi:10.1371/journal.pmed.1002817)
Supplement: S3 Fig — (DOCX) [file pmed.1002817.s003.docx]

# S3 Fig: Tests for publication bias

**Fig A: Child obesity**


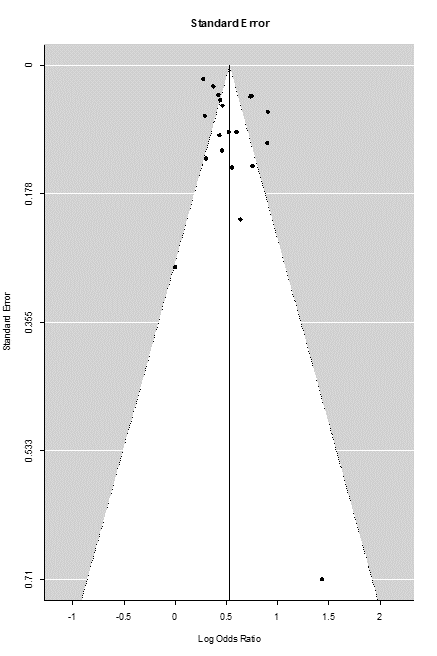


Test for funnel plot asymmetry: z = 0.6313, p = 0.5278

**Fig B: Child overweight and obese**


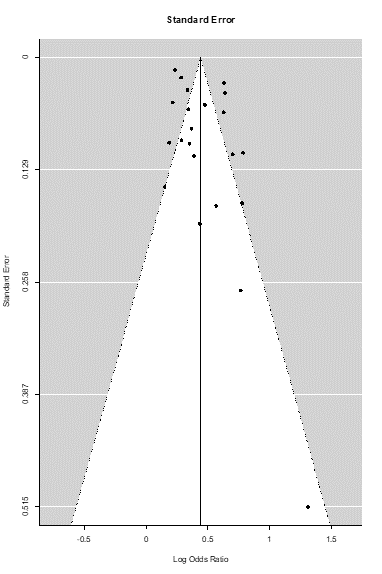


Test for funnel plot asymmetry: z = 1.9484, p = 0.0514

**Fig C: Child overweight**


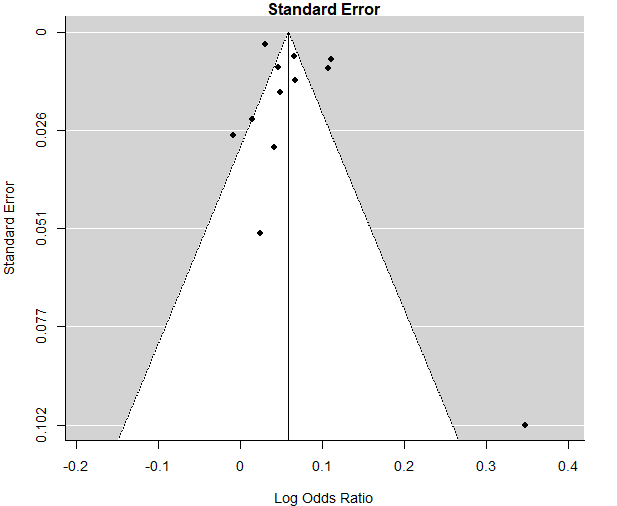


Test for funnel plot asymmetry: z = 0.8166, p = 0.4142

**Fig D: Continuous child BMI and BMI z-score**


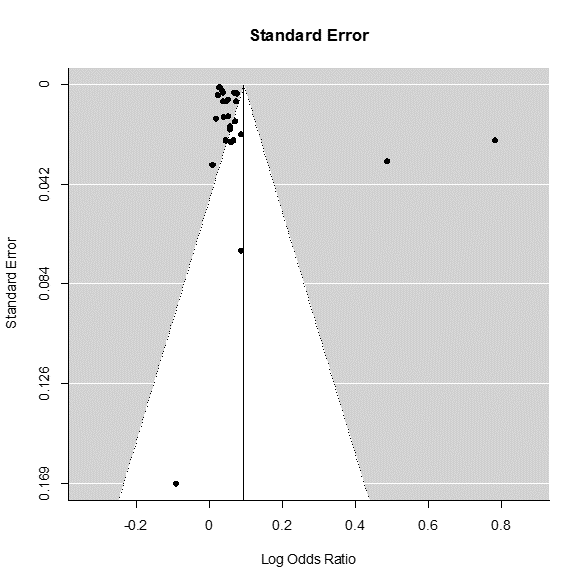


Test for funnel plot asymmetry: z = -0.0060, p = 0.9952
