# Supplementary material for: The association between maternal body mass index and child obesity: A systematic review and meta-analysis
Source: PLoS Med. 2019 Jun 11;16(6):e1002817. doi: 10.1371/journal.pmed.1002817 (PMC6559702; doi:10.1371/journal.pmed.1002817)
Supplement: S4 Fig — (DOCX) [file pmed.1002817.s004.docx]

# S4 Fig: Comparison of linear and nonlinear association between maternal BMI and child overweight or obesity (≥85^th^ percentile)


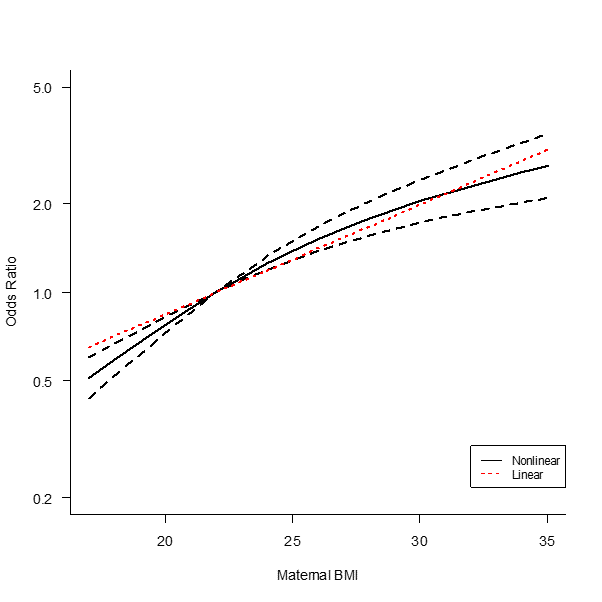


Legend: Pooled dose-response association between maternal BMI and odds of child overweight and obesity. Maternal BMI was modelled with restricted cubic splines in a random-effects dose-response model. Dash lines represent the 95% confidence interval for the spline model. The dotted line represents the linear trend. The value of 22kg/m^2^ served as referent. The odds ratios are plotted on the log scale.
