# Supplementary material for: The association between maternal body mass index and child obesity: A systematic review and meta-analysis
Source: PLoS Med. 2019 Jun 11;16(6):e1002817. doi: 10.1371/journal.pmed.1002817 (PMC6559702; doi:10.1371/journal.pmed.1002817)
Supplement: S6 Fig — (DOCX) [file pmed.1002817.s006.docx]

# S6 Fig: Linear meta-analysis of SMDs for all BMI and BMI z-score outcomes and association with 5-kg/m^2^ increase in maternal BMI

-0.5

0

0.5

1

Coefficient

Mesman et al. 2009[1]

Makela et al. 2013[2]

Leng et al. 2015[3]

Fleten et al. 2012[4]

Daraki et al. 2015[5]

Gademan et al. 2014[6]

Toeman et al. 2016[7]

Kaar et al. 2014[8]

Gaillard et al. 2004[9]

Berkowitz et al. 2005[10]

Kaar et al 2014[8]

Eisenmann et al. 2010[11]

Kaar et al 2014[8]

Kaar et al 2014[8]

Kaar et al 2014[8]

Kaar et al 2014[8]

Kaar et al 2014[8]

Kaar et al 2014[8]

Zalbahar et al. 2015[12]

Li et al. 2013[13]

Deierlein et al. 2011[14]

Margerison Zilko et al. 2012[15]

Jacota et al. 2016[16]

Hinkle et al. 2012[17]

Andres et al. 2015[18]

1

1

3

3

4

5

6

6

6

6

7

7

8

9

10

11

12

13

1

1

3

5

5-6

5

6

3171

150

1263

26587

618

1727

4852

5

4871

70

5

144

14

57

89

86

111

57

145

38539

263

3015

1069

3600

72

0.02 [ 0.01, 0.03]

0.09 [ 0.05, 0.13]

0.04 [ 0.02, 0.05]

0.04 [ 0.03, 0.04]

0.78 [ 0.74, 0.83]

0.07 [ 0.06, 0.09]

0.08 [ 0.07, 0.08]

0.09 [-0.05, 0.22]

0.07 [ 0.06, 0.07]

0.06 [ 0.02, 0.11]

-0.09 [-0.42, 0.24]

0.06 [ 0.01, 0.11]

0.01 [-0.06, 0.08]

0.06 [ 0.02, 0.09]

0.02 [-0.01, 0.05]

0.07 [ 0.04, 0.10]

0.04 [ 0.01, 0.07]

0.06 [ 0.02, 0.09]

0.04 [-0.00, 0.09]

0.03 [ 0.03, 0.03]

0.05 [ 0.03, 0.08]

0.05 [ 0.04, 0.06]

0.05 [ 0.03, 0.06]

0.04 [ 0.03, 0.04]

0.49 [ 0.42, 0.55]

***Continuous child BMI***

***Continuous child BMI z-score***

**Author(s) and Year**

**Child Age**

**Participants**

**(n)**

**Coefficient [95% CI]**

**0.09 [0.01, 0.17]**

**0.10 [-0.02, 0.23]**

Model for BMI (Q = 1117.21, df = 17, p = 0.00; I^2^ = 99.8%)

Model for BMI z-score (Q =

226.02,

df =

6

, p =

0.00

;

I

2

=

99.9

%)

Legend: Pooled summary data for each child BMI outcome type represents the odds ratio and 95% CI for each 1 unit increase in maternal BMI. The size of the data markers indicates the weight assigned to each study in the meta-analysis. Squares represent the odds ratio, bars represent the 95% confidence interval, and diamonds represent the pooled analysis for each child BMI category. Kaar *et al.* 2014[8] has multiple data included in the meta-analysis as these are different children recruited at the same time point for a cross sectional study with different ages rather than data for the same children followed up longitudinally.

**References**

1. Mesman I, Roseboom TJ, Bonsel GJ, Gemke RJ, van der Wal MF, Vrijkotte TGM. Maternal pre-pregnancy body mass index explains infant’s weight and BMI at 14 months: results from a multi-ethnic birth cohort study. Archives of disease in childhood. 2009;94(8):587-95.
2. Makela J, Lagstrom H, Kaljonen A, Simell O, Niinikoski H. Hyperglycemia and lower diet quality in pregnant overweight women and increased infant size at birth and at 13 months of age--STEPS study. Early Human Development. 2013;89(6):439-44.
3. Leng J, Li W, Zhang S, Liu H, Wang L, Liu G, et al. GDM Women's Pre-Pregnancy Overweight/Obesity and Gestational Weight Gain on Offspring Overweight Status. PLoS ONE. 2015;10(6):e0129536.
4. Fleten C, Nystad W, Stigum H, Skjaerven R, Lawlor DA, Davey Smith G, et al. Parent-offspring body mass index associations in the Norwegian Mother and Child Cohort Study: a family-based approach to studying the role of the intrauterine environment in childhood adiposity. Am J Epidemiol. 2012;176(2):83-92.
5. Daraki V, Georgiou V, Papavasiliou S, Chalkiadaki G, Karahaliou M, Koinaki S, et al. Metabolic profile in early pregnancy is associated with offspring adiposity at 4 years of age: the Rhea pregnancy cohort Crete, Greece. PLoS ONE. 2015;10(5):e0126327.
6. Gademan MG, Vermeulen M, Oostvogels AJ, Roseboom TJ, Visscher TL, van Eijsden M, et al. Maternal prepregancy BMI and lipid profile during early pregnancy are independently associated with offspring's body composition at age 5-6 years: the ABCD study. PLoS ONE. 2014;9(4):e94594.
7. Toemen L, Gishti O, Van Osch-Gevers L, Steegers EAP, Helbing WA, Felix JF, et al. Maternal obesity, gestational weight gain and childhood cardiac outcomes: Role of childhood body mass index. Int J Obes. 2016;40(7):1070-8.
8. Kaar JL, Crume T, Brinton JT, Bischoff KJ, McDuffie R, Dabelea D. Maternal obesity, gestational weight gain, and offspring adiposity: the exploring perinatal outcomes among children study. J Pediatr. 2014;165(3):509-15.
9. Gaillard R, Steegers EA, Duijts L, Felix JF, Hofman A, Franco OH, et al. Childhood cardiometabolic outcomes of maternal obesity during pregnancy: the Generation R Study. Hypertension. 2014;63(4):683-91.
10. Berkowitz RI, Stallings VA, Maislin G, Stunkard AJ. Growth of children at high risk of obesity during the first 6 y of life: implications for prevention. Am J Clin Nutr. 2005;81(1):140-6.
11. Eisenman JC, Sarzynski MA, Tucker J, Heelan KA. Maternal prepregnancy overweight and offspring fatness and blood pressure: role of physical activity. Pediatr Exerc Sci. 2010;22(3):369-78.
12. Zalbahar N, Jan Mohamed HJB, Loy SL, Najman J, McIntyre HD, Mamun A. Association of parental body mass index before pregnancy on infant growth and body composition: Evidence from a pregnancy cohort study in Malaysia. Obesity Research and Clinical Practice. 2016;10:S35-S47.
13. Li N, Liu E, Guo J, Pan L, Li B, Wang P, et al. Maternal prepregnancy body mass index and gestational weight gain on offspring overweight in early infancy. PLoS ONE. 2013;8(10):e77809.
14. Deierlein AL, Siega-Riz AM, Chantala K, Herring AH. The association between maternal glucose concentration and child BMI at age 3 years. Diabetes Care. 2011;34(2):480-4.
15. Margerison-Zilko CE, Shrimali BP, Eskenazi B, Lahiff M, Lindquist AR, Abrams BF. Trimester of maternal gestational weight gain and offspring body weight at birth and age five. Matern Child Health J. 2012;16(6):1215-23.
16. Jacota M, Forhan A, Saldanha-Gomes C, Charles MA, Heude B, for the EMCCSG. Maternal weight prior and during pregnancy and offspring's BMI and adiposity at 5–6 years in the EDEN mother–child cohort. Pediatric Obesity. 2016.
17. Hinkle SN, Sharma AJ, Swan DW, Schieve LA, Ramakrishnan U, Stein AD. Excess gestational weight gain is associated with child adiposity among mothers with normal and overweight prepregnancy weight status. J Nutr. 2012;142(10):1851-8.
18. Andres A, Hull HR, Shankar K, Casey PH, Cleves MA, Badger TM. Longitudinal body composition of children born to mothers with normal weight, overweight, and obesity. Obesity (Silver Spring). 2015;23(6):1252-8.
