# Supplementary material for: The association between maternal body mass index and child obesity: A systematic review and meta-analysis
Source: PLoS Med. 2019 Jun 11;16(6):e1002817. doi: 10.1371/journal.pmed.1002817 (PMC6559702; doi:10.1371/journal.pmed.1002817)
Supplement: S10 Fig — (DOCX) [file pmed.1002817.s010.docx]

# S10 Fig: Scatterplot showing the relationship between child age and OR of child overweight (85th to 95th percentile)


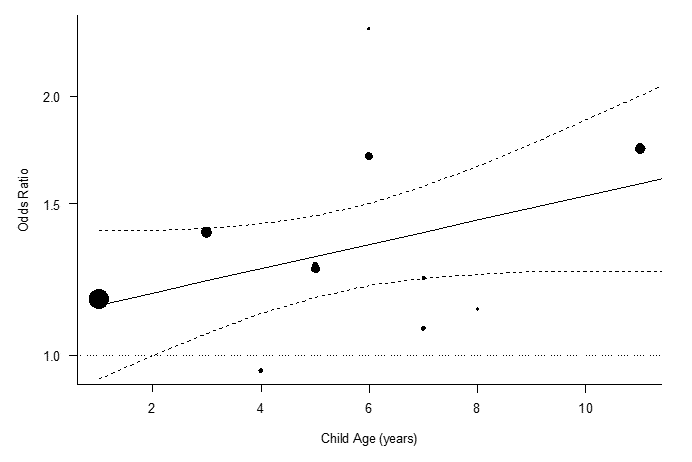


Legend: Dash lines represent the 95% confidence interval for odds ratio. Circles indicate observed odds ratios in individual studies; size of bubbles is proportional to precision (inverse of variance) of the odds ratios.
