# Supplementary material for: The association between maternal body mass index and child obesity: A systematic review and meta-analysis
Source: PLoS Med. 2019 Jun 11;16(6):e1002817. doi: 10.1371/journal.pmed.1002817 (PMC6559702; doi:10.1371/journal.pmed.1002817)
Supplement: S1 Table — (DOCX) [file pmed.1002817.s011.docx]

# S1 Table: MOOSE Checklist [1] for Meta-analyses of Observational Studies

| **Item No** | **Recommendation** | **Reported in manuscript section/paragraph number** |
| --- | --- | --- |
| Reporting of background should include | | |
| 1 | Problem definition | Introduction/1,2 |
| 2 | Hypothesis statement | n/a |
| 3 | Description of study outcome(s) | Methods/2,4 |
| 4 | Type of exposure or intervention used | Methods/2,4 |
| 5 | Type of study designs used | Methods/1,2 |
| 6 | Study population | Methods/2,3 |
| Reporting of search strategy should include | | |
| 7 | Qualifications of searchers (eg, librarians and investigators) | Methods/1 |
| 8 | Search strategy, including time period included in the synthesis and key words | Methods/1  S1 Fig |
| 9 | Effort to include all available studies, including contact with authors | Methods/1  Results/1  S7 Table |
| 10 | Databases and registries searched | Methods/1  S1 Fig |
| 11 | Search software used, name and version, including special features used (eg, explosion) | Methods/1  S1 Fig |
| 12 | Use of hand searching (eg, reference lists of obtained articles) | Methods/1 |
| 13 | List of citations located and those excluded, including justification | Results/1  Fig 1  S3/S4 Tables |
| 14 | Method of addressing articles published in languages other than English | Methods/1  Discussion/2 |
| 15 | Method of handling abstracts and unpublished studies | Methods/1  S4 Table |
| 16 | Description of any contact with authors | Methods/1  Results/1  S7 Table |
| Reporting of methods should include | | |
| 17 | Description of relevance or appropriateness of studies assembled for assessing the hypothesis to be tested | Methods/2-4 |
| 18 | Rationale for the selection and coding of data (eg, sound clinical principles or convenience) | Methods/2-4 |
| 19 | Documentation of how data were classified and coded (eg, multiple raters, blinding and interrater reliability) | Methods/3 |
| 20 | Assessment of confounding (eg, comparability of cases and controls in studies where appropriate) | Methods/7 |
| 21 | Assessment of study quality, including blinding of quality assessors, stratification or regression on possible predictors of study results | Methods/3,7  S2 Fig  S2 Table |
| 22 | Assessment of heterogeneity | Methods/7  S18/S19 Tables  S8-S10 Figs |
| 23 | Description of statistical methods (eg, complete description of fixed or random effects models, justification of whether the chosen models account for predictors of study results, dose-response models, or cumulative meta-analysis) in sufficient detail to be replicated | Methods/3-7  S1 Text |
| 24 | Provision of appropriate tables and graphics | Fig 1  S2 Table  S1/S2 Fig |
| Reporting of results should include | | |
| 25 | Graphic summarizing individual study estimates and overall estimate | Fig 2, Fig 3  S4-S7 Figs |
| 26 | Table giving descriptive information for each study included | S5 Table |
| 27 | Results of sensitivity testing (eg, subgroup analysis) | Results/11  S14-S19 Tables  S8-S10 Figs |
| 28 | Indication of statistical uncertainty of findings | Confidence intervals provided throughout narrative results, tables and figures in the main manuscript and in Supplementary Tables and Figures, and Discussion/2: discussion of limitations |
| Reporting of discussion should include | | |
| 29 | Quantitative assessment of bias (eg, publication bias) | Results/3,5,7,9  S3 Fig |
| 30 | Justification for exclusion (eg, exclusion of non-English language citations) | Discussion/2 |
| 31 | Assessment of quality of included studies | Results/1  S6 Table |
| Reporting of conclusions should include | | |
| 32 | Consideration of alternative explanations for observed results | Discussion/1,2 |
| 33 | Generalization of the conclusions (ie, appropriate for the data presented and within the domain of the literature review) | Discussion/1,3,4 |
| 34 | Guidelines for future research | Discussion/2,3 |
| 35 | Disclosure of funding source | Funding declaration |

**References:**

1. Stroup DF, Berlin JA, Morton SC, et al, for the Meta-analysis Of Observational Studies in Epidemiology (MOOSE) Group. Meta-analysis of Observational Studies in Epidemiology. A Proposal for Reporting. *JAMA*. 2000;283(15):2008-2012.
