# Supplementary material for: The association between maternal body mass index and child obesity: A systematic review and meta-analysis
Source: PLoS Med. 2019 Jun 11;16(6):e1002817. doi: 10.1371/journal.pmed.1002817 (PMC6559702; doi:10.1371/journal.pmed.1002817)
Supplement: S2 Table — (DOCX) [file pmed.1002817.s012.docx]

# S2 Table: Data extraction protocol

**ADAPTED COCHRANE COHORT STUDY DATA EXTRACTION TEMPLATE**

| **Reviewer Initials** |  | |
| --- | --- | --- |
| **Title of Paper** |  | |
| **Author(s)** (List all) |  | |
| **Year of Publication** |  | |
| **Journal** |  | |
| **Conflicts of Interest Declared by Author** | Are there any conflicts of interest?  Yes (please describe)  No  Not reported | |
| **Setting** | Local  Regional  National  International  Location: (region/city, country):  Study name or dataset (if reported): | |
| **Data collection time period** |  | |
| **Study Aim** | Reported Aim:  Is the aim directly related to the influence of prepregnancy BMI on child weight?  Yes, specific to maternal BMI  Yes, measures BMI among other exposures  No | |
| **Final Sample Size** |  | |
| **Ethical Approval Reported** | Yes  Not Reported | |
| **Outcome**  Child Weight Status  Note: Not fat mass or other body composition measure | Data Type: | Categorical  Continuous |
|  | Outcome Measure (e.g. BMI, weight for length): |  |
|  | Is a reference group defined? | Yes (provide details below)  No  Reference Group (name):  Definition: |
|  | Other Groups Reported | Group 1 (name): ………..  Definition: ………..  Group 2 (name): ………..  Definition: ………..  Group 3 (name):………..  Definition: ………..  Group 4 (name):…………  Definition: ……….. |
|  | Child Age Group(s) Reported: |  |
| **Exposure definition**  Prepregnancy or Early Pregnancy BMI | Data Type: | Categorical  Continuous |
|  | Is a reference group defined? | Yes (provide details below)  No  Reference Group (name): ………..  Definition: …….. |
|  | Other Groups Reported | Group 1 (name): Definition: kg/m^2^  Group 2 (name): Definition: kg/m^2^  Group 3 (name): Definition: kg/m^2^  Group 4 (name): Definition: kg/m^2^ |

**Methodology**:  Prospective Cohort  Retrospective Cohort Case Control  Not Defined

|  | **Total group** |
| --- | --- |
| **Number Identified** |  |
| **Number Excluded/Lost to Follow-Up** |  |
| **Final Number Included** |  |
| **All participants accounted for?** | Yes  No  Unclear |

| **Inclusion criteria** | |  |
| --- | --- | --- |
| **Exclusion criteria** | |  |
| **Ascertainment of Exposure –** measure of prepregnancy/early pregnancy BMI | Height | Explicitly measured  Unclear presume measured (e.g. ‘midwife recorded’)  Self-report  Unclear presume self-report (e.g. ‘midwife recorded’)  Unclear  Not reported |
|  | Weight | Explicitly measured  Unclear presume measured (e.g. ‘midwife recorded’)  Self-report  Unclear presume self-report (e.g. ‘midwife recorded’)  Unclear  Not reported |
|  | BMI | Explicitly measured  Unclear presume measured (e.g. ‘midwife recorded’)  Self-report  Unclear presume self-report (e.g. ‘midwife recorded’)  Unclear  Not reported |
| **Ascertainment of Outcome –** child weight | Height | Explicitly measured  Unclear presume measured (e.g. ‘midwife recorded’)  Self-report  Unclear presume self-report (e.g. ‘midwife recorded’)  Unclear  Not reported |
|  | Weight | Explicitly measured  Unclear presume measured (e.g. ‘midwife recorded’)  Self-report  Unclear presume self-report (e.g. ‘midwife recorded’)  Unclear  Not reported |
|  | BMI | Explicitly measured  Unclear presume measured (e.g. ‘midwife recorded’)  Self-report  Unclear presume self-report (e.g. ‘midwife recorded’)  Unclear  Not reported |

**Baseline Characteristics reported?** Yes No **(**if no do not complete, if yes populate with the data**)**

| **Baseline Characteristic**  (include unit of measurement) | **Definition** | **Total cohort** | **Maternal Under weight** | **Maternal Recommended Weight/Reference Group** | **Maternal Over weight** | **Maternal Obese group 1** | **Maternal Obese group 2** | **Maternal Obese group 3** | **P value** |
| --- | --- | --- | --- | --- | --- | --- | --- | --- | --- |
| Maternal Age |  |  |  |  |  |  |  |  |  |
| Marital Status |  |  |  |  |  |  |  |  |  |
| Ethnicity |  |  |  |  |  |  |  |  |  |
| Baseline BMI |  |  |  |  |  |  |  |  |  |
| Smoking Status |  |  |  |  |  |  |  |  |  |
| Gestational Weight Gain |  |  |  |  |  |  |  |  |  |
| Socio-Economic Status |  |  |  |  |  |  |  |  |  |
| Parity |  |  |  |  |  |  |  |  |  |
| Maternal Education Level |  |  |  |  |  |  |  |  |  |
| Income |  |  |  |  |  |  |  |  |  |
| Birthweight |  |  |  |  |  |  |  |  |  |
| Child Sex |  |  |  |  |  |  |  |  |  |

| **Author’s description of any differences between maternal BMI groups:** |  |
| --- | --- |
| **Data Analysis Methods:** |  |
| **Factors Adjusted for in Analysis:** |  |

**Data Analysis: Child BMI … Years Old**

|  | **Child Weight Group 1** N= | **Child Weight Group 2**  N= | **Child Weight Group 3**  N= | **Child Weight Group 4**  N= | **Total Children**  N= | **Unadjusted**  **Type of Statistic** (e.g. OR, RR, mean) | **Crude Statistical Result & CI** | **P value** | **Adjusted**  **Type of Statistic** (e.g. OR, RR, mean) | **Adjusted Statistical Result & CI** | **P value** |
| --- | --- | --- | --- | --- | --- | --- | --- | --- | --- | --- | --- |
| **Maternal Underweight**  N= |  |  |  |  |  |  |  |  |  |  |  |
| **Maternal Recommended Weight/Reference Group**  N= |  |  |  |  |  |  |  |  |  |  |  |
| **Maternal Overweight**  N= |  |  |  |  |  |  |  |  |  |  |  |
| **Maternal Obese group 1**  N= |  |  |  |  |  |  |  |  |  |  |  |
| **Maternal Obese group 2**  N= |  |  |  |  |  |  |  |  |  |  |  |
| **Maternal Obese group 3**  N= |  |  |  |  |  |  |  |  |  |  |  |
