# Supplementary material for: The association between maternal body mass index and child obesity: A systematic review and meta-analysis
Source: PLoS Med. 2019 Jun 11;16(6):e1002817. doi: 10.1371/journal.pmed.1002817 (PMC6559702; doi:10.1371/journal.pmed.1002817)
Supplement: S3 Table — (DOCX) [file pmed.1002817.s013.docx]

# S3 Table: Screening: systematic review reference lists screened, and full papers screened and excluded

**Table A: Reference lists of systematic reviews screened**

| Review | Number of references screened |
| --- | --- |
| Gaillard R, Santos S, Duijts L, Felix JF. Childhood Health Consequences of Maternal Obesity during Pregnancy: A Narrative Review. Ann Nutr Metab. 2017;69(3-4):171-80. | 91 |
| Godfrey KM, Reynolds RM, Prescott SL, *et al.* Influence of maternal obesity on the long-term health of offspring. Lancet Diabetes Endocrinol. 2017;5(1):53-64. | 110 |
| Patro B, Liber A, Zalewski B, Poston L, Szajewska, H, Koletzko B. Maternal and paternal body mass index and offspring obesity: a systematic review. Ann Nutr Metab. 2013;63(1-2):32-41. | 21 |
| Yu Z, Han S, Zhu J, Sun X, Ji C, Guo X. Pre-Pregnancy Body Mass Index in Relation to Infant Birth Weight and Offspring Overweight/Obesity: A Systematic Review and Meta-Analysis. PLoS ONE. 2013;8(4):e61627. | 92 |

**Table B: Original searches - full details of studies excluded following full paper review**

| **References of studies screened in full and excluded** | |
| --- | --- |
| **Exclusion Reason** | **Reference Number** |
| Abstract/poster only | 1-12 |
| Did not report data for analysis of both the exposure and outcome  Child weight status not an outcome  Pre-pregnancy BMI not an exposure | 13-64  13, 15, 17-18, 22, 24, 30, 32, 34, 39, 41-43, 47, 49-51, 53, 57-58, 62-64  14, 16, 19-21, 23, 25-29, 31, 33, 35-38, 30, 44-46, 48, 52, 54-56, 59-61 |
| Unpublished student dissertation | 65 |
| **Reference list of excluded studies** | |
| 1. Decrausaz SL, Stock JT, Fewtrell MS, Williams JE, Wells JCK. Maternal and paternal anthropometry influences on body size, body shape and obstetric capacity in growing girls. Am J Phys Anthropol. 2017;162:159. 2. Donnelly J, Walsh J, Horan M, Molloy EJ, McAuliffe F. Parental and perinatal pre-determinants of offspring adiposity at 2 years of age-analysis from the ROLO Kids study. Eur J Pediatr. 2016;175 (11):1449. 3. Eny KM, Chen S, Anderson LN, *et al.* Breastfeeding duration, maternal BMI and birthweight are associated with differences in BMI growth trajectories in early childhood. Obes Rev. 2016;17:92. 4. Eriksson J. Long-term consequences of maternal obesity on the health of offspring. Human Repro. 2016;31:i1. 5. Kerr JA, Long C, Clifford SA, Muller J, Hanvey AN, Wake M. Early life factors predicting change in childhood body mass index. Obesity Facts. 2016;9:40. 6. Mamun A. Overnutrition hypothesis using parental-offspring associations - a 30 years follow-up of a community based large birth cohort study. Obes Rev. 2016;17:65. 7. Nazareth M, Pinto E, Lopes C, Rego C. Influence of parental nutritional status on nutritional status of their offspring at 0-36 months. Results from EPACI Portugal 2012. Obesity Facts. 2017;10:239-240. 8. Thomas M, Gunzler D, Presley L, Catalano P. Maternal obesity and infant growth. Diabetes. 2017;66:A388-A389. 9. Wall CR, Murphy R, Waldie KE, Mitchell EA, Thompson JMD. The effects of maternal and early childhood dietary patterns on BMI, percentage body fat and sleep at 7 years. Cogent Medicine. Conference: 8th Excellence in Pediatrics Conference. 2016;3(1). 10. Wraw C, Gale C, Der G, Deary I. Maternal and offspring intelligence in relation to BMI across childhood and adolescence. Obesity Facts. 2017;10:22. 11. Zalbahar N, Jan Mohamed HJB, Loy SL, Najman J, McIntyre HD, Mamun A. Association of parental body mass index before pregnancy on infant growth and body composition: Evidence from a pregnancy cohort study in Malaysia. Obes Res Clin Pract. 2016;10:S35-S47. 12. Zulfqar T, Strazdins L, D'Este C, Dinh H, Banwell C. Maternal generation and overweight and obesity in 2-11 year old children: Evidence from birth cohort of the longitudinal study of Australian children. Obesity Facts. 2017;10:11. 13. Andersen CH, Thomsen PH, Nohr EA, Lemcke S. Maternal Body Mass Index Before Pregnancy as A Risk Factor for ADHD and Autism in Children. Eur Child Adolesc Psychiatry. 2017:1-10. 14. Aris IM, Bernard JY, Chen LW, *et al.* Modifiable Risk Factors of Early Childhood Overweight and Obesity in an Asian Population. Obes Rev. 2016;17:114 15. Aris IM, Soh SE, Tint MT, *et al.* Associations of Gestational Glycemia and Prepregnancy Adiposity with Offspring Growth and Adiposity in an Asian Population. Am J Clin Nutr. 2015;102(5):1104-12. 16. Araujo MLD, Cabral PC, De Arruda IKG, *et al.* Excessive Gain in Body Mass Index-for-age Z-score and Associated Factors: A Cohort Study in Female Adolescents. Eur J Clinl Nutr. 2017;71(4):525-29. 17. Baptiste-Roberts K, Nicholson WK, Wang NY, Brancati FL. Gestational Diabetes and Subsequent Growth Patterns of Offspring: The National Collaborative Perinatal Project. MatChild Health J. 2012;16(1):125-32. 18. Brandt S, Mos A, Lennerz B, *et al.* Plasma Insulin Levels in Childhood Are Related to Maternal Factors - Results of the Ulm Birth Cohort Study. Pediatr Diabetes. 2014;15(6):453-63. 19. Bornhorst C, Siani A, Russo P, *et al.* Early Life Factors and Inter-Country Heterogeneity in BMI Growth Trajectories of European Children: The IDEFICS Study. PLoS ONE. 2016;11(2) 20. Chen CM, Lou MF, Gau BS. Parental Body Mass Index Is Associated with Adolescent Obesity in Taiwan. Res NursHealth. 2016;39(6):399-405. 21. Chen LW, Aris IM, Bernard JY, *et al.* Associations of Maternal Macronutrient Intake During Pregnancy with Infant BMI Peak Characteristics and Childhood BMI. Am J Clinl Nutr. 2017;105(3):705 -13. 22. Chen LW, Tint MT, Fortier MV, *et al.* Maternal Macronutrient Intake During Pregnancy Is Associated with Neonatal Abdominal Adiposity: The Growing Up in Singapore Towards Healthy Outcomes (GUSTO) Study. J Nutr. 2016;146(8):1571-79. 23. Chiasson MA, Scheinmann R, Hartel D, *et al.* Predictors of Obesity in a Cohort of Children Enrolled in WIC as Infants and Retained to 3 Years of Age. J Comm Health. 2016;41(1):127-33. 24. Deardorff J, Berry-Millett R, Rehkopf D, Luecke E, Lahiff M, Abrams B. Maternal Pre-Pregnancy BMI, Gestational Weight Gain, and Age at Menarche in Daughters. Matern Child Health J. 2013;17(8):1391-8. 25. Duchin O, Marin C, Mora-Plazas M, Villamor E. Maternal Body Image Dissatisfaction and BMI Change in School-Age Children. Public Health Nutr. 2016;19(2):287-92. 26. Diesel JC, Eckhardt CL, Day NL, Brooks MM, Arslanian SA, Bodnar LM. Gestational Weight Gain and the Risk of Offspring Obesity at 10 and 16 Years: A Prospective Cohort Study in Low-Income Women. BJOG: An International Journal of Obstetrics & Gynaecology. 2015;122(10):1395-402. 27. Ensenauer R, Chmitorz A, Riedel C, *et al.* Effects of Suboptimal or Excessive Gestational Weight Gain on Childhood Overweight and Abdominal Adiposity: Results from a Retrospective Cohort Study. Int J Obes. 2013;37(4):505-12. 28. Figueiredo RAO, Roos E, Eriksson JG, Simola-Strom S, Weiderpass E. Maternal Alcohol and Tobacco Consumption and the Association with their 9 to 14-year-old Children's Body Mass Index. Scand J Public Health. 2017;45(5):503-10. 29. Fraser A, Tilling K, Macdonald-Wallis C,. Association of maternal weight gain in pregnancy with offspring obesity and metabolic and vascular traits in childhood. Circulation. 2010;121(23):2557-64. 30. Gao X, Yan Y, Xiang S, *et al.* The Mutual Effect of Pre-pregnancy Body Mass Index, Waist Circumference and Gestational Weight Gain on Obesity-related Adverse Pregnancy Outcomes: A Birth Cohort Study. PLoS ONE. 2017;12(6). 31. Geckil E, Aslan S, Ister ED, Simsek DK, Sahin T. Prevalence and Risk Factors of Obesity and Overweight in Elementary School-Age (5 to 15 years old) Children in South-Eastern Turkey. Iran J Pediatr. 2017;27(2). 32. Getz KD, Anderka MT, Werler MM, Jick SS. Maternal Pre-pregnancy Body Mass Index and Autism Spectrum Disorder among Offspring: A Population-Based Case-Control Study. Paed Perinat Epidemiol. 2016;30(5):479-87. 33. Gibson LY, Allen KL, Byrne SM, *et al.* Childhood Overweight and Obesity: Maternal and Family Factors. J Child Family Studies. 2016;25(11):3236-46. 34. Griffiths LJ, Hawkins SS, Cole TJ, Dezateux C, Millennium Cohort Study Child Health G. Risk Factors for Rapid Weight Gain in Preschool Children: Findings from a UK-Wide Prospective Study. Int J Obes. 2010;34(4):624-32. 35. Grummer-Strawn LM, Mei Z. Does Breastfeeding Protect against Pediatric Overweight? Analysis of Longitudinal Data from the Centers for Disease Control and Prevention Pediatric Nutrition Surveillance System. Pediatrics. 2004;113(2):e81-6. 36. Hassan NE, Wahba S, El-Alameey IR, *et al.* Dietary Behaviour Pattern and Physical Activity in Overweight and Obese Egyptian Mothers: Relationships with their Children's Body Mass Index. Macedonian Journal of Medical Sciences. 2016;4(3):353-58. 37. Hohwu L, Zhu JL, Graversen L, Li J, Sorensen TI, Obel C. Prenatal Parental Separation and Body Weight, Including Development of Overweight and Obesity Later in Childhood. PLoS ONE. 2015;10(3):e0119138. 38. Huh SY, Rifas-Shiman SL, Zera CA, *, et al.* Delivery by Caesarean Section and Risk of Obesity in Preschool Age Children: A Prospective Cohort Study. Archives of Disease in Childhood. 2012;97(7):610-6. 39. Jensen ET, van der Burg JW, O'Shea TM, *et al.* The Relationship of Maternal Prepregnancy Body Mass Index and Pregnancy Weight Gain to Neurocognitive Function at Age 10 Years among Children Born Extremely Preterm. Journal of Pediatrics. 2017;11. 40. Kain J, Corvalan C, Lera L, Galvan M, Uauy R. Accelerated Growth in Early Life and Obesity in Preschool Chilean Children. Obesity. 2009;17(8):1603-8. 41. Koupil I, Toivanen P. Social and Early-Life Determinants of Overweight and Obesity in 18-Year-Old Swedish Men. International Journal of Obesity. 2008;32(1):73-81. 42. Larsen SC, Angquist L, Laurin C, *et al.* Association Between Maternal Fish Consumption and Gestational Weight Gain: Influence of Molecular Genetic Predisposition to Obesity. PLoS ONE. 2016;11(3). 43. Lawlor DA, Lichtenstein P, Langstrom N. Association of Maternal Diabetes Mellitus in Pregnancy with Offspring Adiposity into Early Adulthood: Sibling Study in a Prospective Cohort of 280,866 Men from 248,293 Families. Circulation. 2011;123(3):258-65. 44. Lawlor DA, Smith GD, O'Callaghan M, *et al.* Epidemiologic Evidence for the Fetal Overnutrition Hypothesis: Findings from the Mater-University Study of Pregnancy and Its Outcomes. American Journal of Epidemiology. 2007;165(4):418-24. 45. Lawrence GM, Shulman S, Friedlander Y, *et al.* Associations of Maternal Pre-Pregnancy and Gestational Body Size with Offspring Longitudinal Change in BMI. Obesity. 2014;22(4):1165-71. 46. Lin X, Aris IM, Tint MT, *et al.* Ethnic Differences in Effects of Maternal Pre-Pregnancy and Pregnancy Adiposity on Offspring Size and Adiposity. Journal of Clinical Endocrinology & Metabolism. 2015;100(10):3641-50. 47. Macdonald KD, Vesco KK, Funk KL, *et al.* Maternal Body Mass Index Before Pregnancy is Associated with Increased Bronchodilator Dispensing in Early Childhood: A Cross-sectional Study. Pediatric Pulmonology. 2016. 48. Manios Y, Birbilis M, Moschonis G, *et al.* Childhood Obesity Risk Evaluation Based on Perinatal Factors and Family Sociodemographic Characteristics: Core Index. European Journal of Pediatrics. 2013;172(4):551-5. 49. Margerison Zilko CE, Rehkopf D, Abrams B. Association of Maternal Gestational Weight Gain with Short- and Long-Term Maternal and Child Health Outcomes. American Journal of Obstetrics and Gynecology. 2010;202(6):574.e1-8. 50. Nagl M, Steinig J, Klinitzke G, Stepan H, Kersting A. Childhood Maltreatment and Pre-pregnancy Obesity: A Comparison of Obese, Overweight, and Normal Weight Pregnant Women. Archives of Women's Mental Health. 2016;19(2):355-65. 51. Neutzling MB, Hallal PR, Araujo CL, *et al.* Infant Feeding and Obesity at 11 Years: Prospective Birth Cohort Study. International Journal of Pediatric Obesity. 2009;4(3):143-9. 52. Oostvogels AJ, Stronks K, Roseboom TJ, van der Post JA, van Eijsden M, Vrijkotte TG. Maternal Prepregnancy BMI, Offspring's Early Postnatal Growth, and Metabolic Profile at Age 5-6 Years: The ABCD Study. Journal of Clinical Endocrinology & Metabolism. 2014;99(10):3845-54. 53. Page KA, Romero A, Buchanan TA, Xiang AH. Gestational Diabetes Mellitus, Maternal Obesity, and Adiposity in Offspring. Journal of Pediatrics. 2014;164(4):807-10. 54. Parrino C, Vinciguerra F, La Spina N, *et al.* Influence of Early-life and Parental Factors on Childhood Overweight and Obesity. Journal of Endocrinological Investigation. 2016;39(11):1315-21. 55. Pei Z, Heinrich J, Fuertes E, *et al.* Cesarean Delivery and Risk of Childhood Obesity. Journal of Pediatrics. 2014;164(5):1068-73. 56. Razaz N, Tedroff K, Villamor E, Cnattingius S. Maternal Body Mass Index in Early Pregnancy and Risk of Epilepsy in Offspring. JAMA Neurology. 2017;74(6):668-76. 57. Schack-Nielsen L, Michaelsen KF, Gamborg M, Mortensen EL, Sorensen TI. Gestational Weight Gain in Relation to Offspring Body Mass Index and Obesity from Infancy through Adulthood. International Journal of Obesity. 2010;34(1):67-74. 58. Sridhar SB, Darbinian J, Ehrlich SF, *et al.* Maternal Gestational Weight Gain and Offspring Risk for Childhood Overweight or Obesity. Am J Obstet Gynecol. 2014;211(3):259.e1-8. 59. Stuebe AM, Forman MR, Michels KB. Maternal-Recalled Gestational Weight Gain, Pre-Pregnancy Body Mass Index, and Obesity in the Daughter. Int J Obes. 2009;33(7):743-52. 60. Taveras EM, Gillman MW, Kleinman K, Rich-Edwards JW, Rifas-Shiman SL. Racial/Ethnic Differences in Early-Life Risk Factors for Childhood Obesity. Pediatrics. 2010;125(4):686-95. 61. Wen X, Triche EW, Hogan JW, Shenassa ED, Buka SL. Prenatal Factors for Childhood Blood Pressure Mediated by Intrauterine and/or Childhood Growth? Pediatrics. 2011;127(3):e713-21. 62. Westberg AP, Salonen MK, Von Bonsdorff M, Kajantie E, Eriksson JG. Maternal Body Mass Index in Pregnancy and Offspring Physical and Psychosocial Functioning in Older Age: Findings from the Helsinki Birth Cohort Study (HBCS). Ann Med. 2016;48(4):268-74. 63. Wright CM, Emmett PM, Ness AR, Reilly JJ, Sherriff A. Tracking of Obesity and Body Fatness through Mid-Childhood. Arch Dis Child. 2010;95(8):612-7. 64. Zhao P, Liu E, Qiao Y, *et al.* Maternal Gestational Diabetes and Childhood Obesity at Age 9-11: Results of a Multinational Study. Diabetologia. 2016;59(11):2339-48. 65. Potential determinants and mechanisms responsible for obesity trajectories during early childhood, Park Hyojun: The University of Wisconsin – Madison, Population Health, US; 2017. | |

**Table C: Updated searches - full details of studies excluded following full paper review**

| **References of studies screened in full and excluded** | |
| --- | --- |
| **Exclusion Reason** | **Reference Number** |
| Abstract/poster only | 1-11 |
| Did not report data for analysis of both the exposure and outcome |  |
| Child weight status not an outcome | 12-13 |
| Pre-pregnancy BMI not an exposure | 14-38 |
| Associations between maternal and child weight status not reported | 39-67 |
| Child age less than 1 year or more than 19 years | 68-71 |
| Commentary only | 72 |
| **Reference list of excluded studies** | |
| 1. Decrausaz, S. L., J. T. Stock, M. S. Fewtrell, J. E. Williams and J. C. K. Wells (2017). "Maternal and paternal anthropometry influences on body size, body shape and obstetric capacity in growing girls." American Journal of Physical Anthropology 162: 159. 2. Erenberg, M., D. Landau, I. S. Vardi, H. Vardi, E. Sheiner and N. Bilenko (2018). "Pre-pregnancy overweight, obesity and excessive gestational weight gain, and the risk for large for gestational age and excessive neonatal weight gain during the first year of life." American Journal of Obstetrics and Gynecology 218 (1 Supplement 1): S402. 3. Falahi, F., L. K. Kupers, E. Corpeleijn and H. Snieder (2018). "Higher body mass index (BMI) and overweight/obesity in children with intrauterine smoke exposure." Obesity Facts 11 (Supplement 1): 55-56. 4. Fang, H., C. Lin, Y. Huang, Z. Tsai, C. Chiu, P. Kao, K. Lu, W. Lee, G. Chun-Chun, H. Tsai and T. Yao (2018). "Maternal pre-pregnancy overweight/obesity and development of allergic rhinitis and asthma in their offspring at age of 6 years." Allergy: European Journal of Allergy and Clinical Immunology 73 (Supplement 105): 333. 5. Handel, M., S. Larsen, J. Rohde, M. Stougaard, N. Olsen and B. Heitmann (2017). "Effects on physical activity patterns among preschool children predisposed to overweight and obesity in the Danish Healthy Start intervention." Obesity Facts 10: 233. 6. Murray, R., P. Titcombe, S. Barton, C. Cooper, H. Inskip, M. Hanson, K. Godfrey and K. Lillycrop (2018). "Maternal adiposity in late pregnancy associates with altered DNA methylation at the CDKN2A cluster." International Journal of Gynecology and Obstetrics 143 (Supplement 3): 187-188. 7. Ng, S., S. Y. Chan, I. Aris, M. T. Tint, L. W. Chen, P. Gluckman, K. Godfrey, F. Yap, K. H. Tan, Y. S. Lee and Y. S. Chong (2018). "Maternal circulating cotinine concentration in pregnancy and the trajectory of offspring weight and body mass index (BMI) in the first 36 months of life." BJOG: An International Journal of Obstetrics and Gynaecology 125 (Supplement 1): 114. 8. Saez, I. B. I., C. S. Gallardo, C. C. Duran and J. P. Acosta (2018). "Association between early rapid weight gain, duration of exclusive breastfeeding and maternal factors with excess malnutrition at 5 years of age." Pediatric Research 84 (3): 468. 9. Thomas, M., D. Gunzler, L. Presley and P. Catalano (2017). "Maternal obesity and infant growth." Diabetes 66: A388-A389. 10. Wang, X., M. P. Martinez and A. Xiang (2018). "Maternal obesity, diabetes during pregnancy, gestational weight gain, breastfeeding, and child's BMI growth trajectory from ages 2 to 6 years." Diabetes 67 (Supplement 1): A92. 11. Yamanouchi, L., M. C. Vieira, F. Miller, H. Gu, P. Seed, P. J. Chowienczyk, D. Pasupathy, L. Poston and P. D. Taylor (2018). "Maternal early-pregnancy BMI and determinants of childhood heart rate variability, blood pressure and cardiac structure at 3 years: UPBEAT TEMPO HEART study." Reproductive Sciences 25 (1): 267A. 12. Anderson, L. N., J. A. Knight, R. J. Hung, S. L. Hewko, R. A. Seeto, M. J. Martin, A. Fleming, J. L. Maguire, S. G. Matthews, K. E. Murphy, N. Okun, J. M. Jenkins, S. J. Lye, A. Bocking, R. Levitan, K. Murphy, A. Azad, J. Jenkins, M. Sermer, P. Shah, J. Shapiro, S. Hewko, R. Seeto, K. Foshay, M. Rocco, C. Brush, T. Selander, A. Patel, A. Oduwole and P. Saban (2018). "The Ontario Birth Study: A prospective pregnancy cohort study integrating perinatal research into clinical care." Paediatric and Perinatal Epidemiology 32(3): 290-301. 13. Deardorff, J., L. H. Smith, L. Petito, H. Kim and B. F. Abrams (2017). "Maternal Prepregnancy Weight and Children's Behavioral and Emotional Outcomes." American Journal of Preventive Medicine. 14. Winter, J. D., Y. Taylor, L. Mowrer, K. M. Winter and M. F. Dulin (2017). "BMI at birth and overweight at age four." Obesity Research and Clinical Practice 11(2): 151-157. 15. Jalali-Farahani, S., P. Amiri, B. Abbasi, M. Karimi, L. Cheraghi, M. S. Daneshpour and F. Azizi (2017). "Maternal Characteristics and Incidence of Overweight/Obesity in Children: A 13-Year Follow-up Study in an Eastern Mediterranean Population." Maternal and child health journal 21(5): 1211-1220. 16. Kondolot, M., S. Poyrazoglu, D. Horoz, A. Borlu, C. Altunay, E. Balcl, A. Ozturk, M. M. Mazlcloglu and S. Kurtoglu (2017). "Risk factors for overweight and obesity in children aged 2-6 years." Journal of Pediatric Endocrinology and Metabolism 30(5): 499-505. 17. A, N., P. Venkataraman, C. Kanniammal, A. Rani and J. Arulappan (2018). "Prevalence of Obesity and Associated Risk Factors among Adolescents in Kancheepuram, South India." International Journal of Nursing Education 10(1): 55-60. 18. Alhassan, B. A., Y. Liu, D. Slawson, J. M. Peterson, J. A. Marrs, W. A. Clark and A. Alamian (2018). "The influence of maternal body mass index and physical activity on select cardiovascular risk factors of preadolescent Hispanic children." PeerJ 2018 (12) (e6100). 19. Almari, M., S. Alsaedi, A. Mohammad and A. H. Ziyab (2018). "Associations of adiposity and parental diabetes with prediabetes among adolescents in Kuwait: A cross-sectional study." Pediatric Diabetes 19(8): 1362-1369. 20. Amigo, H., P. Bustos and R. Rona (2017). "The limache birth cohort study in Chile." Annals of Nutrition and Metabolism 71 (Supplement 2): 230. 21. Amini, M., M. D. Piraghaj, M. Khosravi and N. Lotfollahi (2018). "Dietary Patterns and Obesity Associated Factors in Primary School Children." Nutrition & Food Sciences Research 5(1): 1-8. 22. Amugsi, D. A., Z. T. Dimbuene, E. W. Kimani-Murage, B. Mberu and A. C. Ezeh (2017). "Differential effects of dietary diversity and maternal characteristics on linear growth of children aged 6-59 months in sub-Saharan Africa: a multi-country analysis." Public health nutrition 20(6): 1029-1045. 23. Ardic, C., O. Usta, E. Omar, C. Yildiz and E. Memis (2019). "Effects of infant feeding practices and maternal characteristics on early childhood obesity." Archivos Argentinos de Pediatria 117(1): 26-33. 24. Barzin, M., S. Aryannezhad, S. Serahati, A. Beikyazdi, F. Azizi, M. Valizadeh, M. Ziadlou and F. Hosseinpanah (2018). "Incidence of obesity and its predictors in children and adolescents in 10 years of follow up: Tehran lipid and glucose study (TLGS)." BMC Pediatrics 18 (1) (no pagination)(245). 25. El Kabbaoui, M., A. Chda, A. Bousfiha, L. Aarab, R. Bencheikh and A. Tazi (2018). "Prevalence of and risk factors for overweight and obesity among adolescents in Morocco." Eastern Mediterranean Health Journal 24(6): 512-521. 26. Geckil, E., S. Aslan, E. D. Ister, D. K. Simsek and T. Sahin (2017). "Prevalence and risk factors of obesity and overweight in elementary School-Age (5 to 15 years old) children in South-eastern Turkey." Iranian Journal of Pediatrics 27 (2) (e7218). 27. Greene-Cramer, B., M. B. Harrell, D. M. Hoelscher, S. Sharma, N. Ranjit, V. Gupta, G. Nazar and M. Arora (2018). "Association between parent and child weight status among private school children in Delhi, India." Global health promotion 25(2): 67-74. 28. Gregori, D., A. Hochdorn, D. Azzolina, P. Berchialla and G. Lorenzoni (2018). "Does Love Really Make Mothers Blind? A Large Transcontinental Study on Mothers' Awareness About Their Children's Weight." Obesity 26(7): 1211-1224. 29. Gridneva, Z., A. Rea, A. R. Hepworth, L. C. Ward, C. T. Lai, P. E. Hartmann and D. T. Geddes (2018). "Relationships between breastfeeding patterns and maternal and infant body composition over the first 12 months of lactation." Nutrients 10 (1)(45). 30. Hirschler, V., M. Martin, K. Oestreicher, C. Molinari, L. Boero, W. Tetzlaff and F. Brites (2017). "Association between apo B levels in mothers and in their pre-school age offspring." Cardiovascular and Hematological Agents in Medicinal Chemistry 15(1): 62-68. 31. Hoang, N. T. D., L. Orellana, T. D. Le, R. S. Gibson, A. F. Worsley, A. J. Sinclair and E. A. Szymlek-Gay (2018). "Anthropometric Status among 6–9-Year-Old School Children in Rural Areas in Hai Phong City, Vietnam." Nutrients 10(10): 1431. 32. Kerr, J. A., C. Long, S. A. Clifford, J. Muller, A. N. Gillespie, S. Donath and M. Wake (2017). "Early-life exposures predicting onset and resol of childhood overweight or obesity." Archives of Disease in Childhood 102(10): 922-929. 33. Kowal, M., S. Matusik, M. W. Pilecki, L. Kryst, J. Sobiecki and A. Woronkowicz (2017). "Overweight and obesity risk factors in children aged 3-7 years: a prospective study in the city of Krakow." Annals of human biology 44(8): 693-703. 34. Mueller, N., M. L. Garmendia, M. Reyes, C. Corvalan, A. Pereira and R. Uauy (2018). "Female offspring birth weight is associated with Body Mass Index, waist circumference and metabolic syndrome in Latin American women at 10-years postpartum." Diabetes Research and Clinical Practice 138: 90-98. 35. Naess, M., E. R. Sund, T. L. Holmen and K. Kvaloy (2018). "Implications of parental lifestyle changes and education level on adolescent offspring weight: A population based cohort study - The HUNT Study, Norway." BMJ Open 8 (8)(e023406). 36. Swanton, S., A. C. Choh, M. Lee, L. L. Laubach, J. K. Linderman, S. A. Czerwinski and M. J. Peterson (2017). "Body mass index associations between mother and offspring from birth to age 18: the Fels Longitudinal Study." Obesity Science and Practice 3(2): 127-133. 37. Vehapoglu, A., N. Goknar, O. Turel, E. Torun and G. Ozgurhan (2017). "Risk factors for childhood obesity: Do the birth weight, type of delivery, and mother's overweight have an implication on current weight status?" World Journal of Pediatrics 13(5): 457-464. 38. Hazrati, S., W. S. W. Wong, K. Huddleston, Y. Yui, N. Gilchrist, B. D. Solomon, J. Niederhuber and S. K. Hourigan (2018). "Clinical, Social, and Genetic Factors Associated with Obesity at 12 Months of Age." Journal of Pediatrics 196: 175-181.e177. 39. West, J., G. Santorelli, P. H. Whincup, L. Smith, N. A. Sattar, N. Cameron, D. Farrar, P. Collings, J. Wright and D. A. Lawlor (2018). "Association of maternal exposures with adiposity at age 4/5 years in white British and Pakistani children: findings from the Born in Bradford study." Diabetologia 61(1): 242-252. 40. AbdelHamid, E. R. (2017). "Nutritional behaviour pattern and physical activity in obese and overweight egyptian mothers: Relationships with their children's body mass index." European Journal of Pediatrics 176 (11): 1459. 41. Bar-Meir, M., Y. Friedlander, R. Calderon-Margalit and H. Hochner (2019). "Mode of delivery and offspring adiposity in late adolescence: The modifying role of maternal pre-pregnancy body size." PLoS ONE 14 (1)(e0209581). 42. Barros, A. J., L. P. Santos, F. Wehrmeister, J. V. Motta, A. Matijasevich, I. S. Santos, A. M. Menezes, H. Goncalves, M. C. Assuncao, B. L. Horta and F. C. Barros (2017). "Caesarean section and adiposity at 6, 18 and 30 years of age: results from three Pelotas (Brazil) birth cohorts." BMC public health 17(1): 256. 43. Chaparro, M. P., I. Koupil and L. Byberg (2017). "Maternal pre-pregnancy BMI and offspring body composition in young adulthood: the modifying role of offspring sex and birth order." Public health nutrition 20(17): 3084-3089. 44. Chen, L. W., I. M. Aris, J. Y. Bernard, M. T. Tint, A. Chia, M. Colega, P. D. Gluckman, L. P. C. Shek, S. M. Saw, Y. S. Chong, F. Yap, K. M. Godfrey, R. M. Van Dam, M. F. F. Chong and Y. S. Lee (2017). "Associations of maternal dietary patterns during pregnancy with offspring adiposity from birth until 54 months of age." Nutrients 9 (1) (2). 45. Chen, L. W., I. M. Aris, J. Y. Bernard, M. T. Tint, M. Colega, P. D. Gluckman, K. H. Tan, L. P. C. Shek, Y. S. Chong, F. Yap, K. M. Godfrey, R. M. Van Dam, M. F. F. Chong and Y. S. Lee (2017). "Associations of maternal macronutrient intake during pregnancy with infant BMI peak characteristics and childhood BMI." American Journal of Clinical Nutrition 105(3): 705-713. 46. Graversen, L., L. D. Howe, T. I. Sorensen, U. Sovio, L. Hohwu, K. Tilling, J. Laitinen, A. Taanila, A. Pouta, M. R. Jarvelin and C. Obel (2017). "Body mass index trajectories from 2 to 18years - exploring differences between European cohorts." Pediatric Obesity 12(2): 102-109. 47. Hammoud, N. M., G. H. A. Visser, L. van Rossem, D. H. Biesma, J. M. Wit and H. W. de Valk (2018). "Long-term BMI and growth profiles in offspring of women with gestational diabetes." Diabetologia 61(5): 1037-1045. 48. Holdsworth, E. A. and L. M. Schell (2017). "Maternal-infant interaction as an influence on infant adiposity." American journal of human biology: the official journal of the Human Biology Council 29(5). 49. Huang, Y., B. Yin, X. Liang, H. Mei, H. Lu, S. Xie, W. Bei, W. Mei and J. Zhang (2017). "Effect of maternal glycemia and weight status on offspring birth measures and BMI-z among Chinese population in the first year." Scientific reports 7(1): 16030. 50. Mourtakos, S. P., K. D. Tambalis, D. B. Panagiotakos, G. Antonogeorgos, C. D. Alexi, M. Georgoulis, G. Saade and L. S. Sidossis (2017). "Association between gestational weight gain and risk of obesity in preadolescence: a longitudinal study (1997-2007) of 5125 children in Greece." Journal of human nutrition and dietetics: the official journal of the British Dietetic Association 30(1): 51-58. 51. Mueller, N. T., S. L. Rifas, J. Chavarro, E. Oken and M. F. Hivert (2017). "Associations of delivery mode and labor with measures of childhood adiposity: Findings from Project Viva." FASEB Journal. Conference: Experimental Biology 31(1 Supplement 1). 52. Njuieyon, F., E. Cuadro-Alvarez, E. Martin, N. Lachaume, Y. Mrsic, F. Henaff, C. Maniassom, A. Defo and N. Elenga (2018). "Mother's obesity and high child's waist circumference are predictive factors of severe child's obesity: An observational study in French Guiana." BMC Pediatrics 18 (1)(188). 53. Pitchika, A., K. Vehik, S. Hummel, J. M. Norris, U. M. Uusitalo, J. Yang, S. M. Virtanen, S. Koletzko, C. Andren Aronsson, A. G. Ziegler and A. Beyerlein (2018). "Associations of Maternal Diabetes During Pregnancy with Overweight in Offspring: Results from the Prospective TEDDY Study." Obesity 26(9): 1457-1466. 54. Pringle, K. G., Y. Q. Lee, L. Weatherall, L. Keogh, C. Diehm, C. T. Roberts, S. Eades, A. Brown, R. Smith, E. R. Lumbers, L. J. Brown, C. E. Collins and K. M. Rae (2018). "Influence of maternal adiposity, preterm birth and birth weight centiles on early childhood obesity in an indigenous Australian pregnancy through to early childhood cohort study." Reproductive Sciences 25 (1): 133A. 55. Rzehak, P., W. H. Oddy, M. L. Mearin, V. Grote, T. A. Mori, H. Szajewska, R. Shamir, S. Koletzko, M. Weber, L. J. Beilin, R. C. Huang and B. Koletzko (2017). "Infant feeding and growth trajectory patterns in childhood and body composition in young adulthood." American Journal of Clinical Nutrition 106(2): 568-580. 56. Santos Ferreira, D. L., D. M. Williams, A. J. Kangas, P. Soininen, M. Ala-Korpela, G. D. Smith, M. R. Jarvelin and D. A. Lawlor (2017). "Association of pre-pregnancy body mass index with offspring metabolic profile: Analyses of 3 European prospective birth cohorts." PLoS Medicine 14 (8) (e1002376). 57. Schott, W., E. Aurino, M. E. Penny and J. R. Behrman (2018). "Adolescent mothers' anthropometrics and grandmothers' schooling predict infant anthropometrics in Ethiopia, India, Peru, and Vietnam." Annals of the New York Academy of Sciences 1416(1): 86-106. 58. Sundholm, J. K. M., L. Litwin, K. Rono, S. B. Koivusalo, J. G. Eriksson and T. Sarkola (2019). "Maternal obesity and gestational diabetes: Impact on arterial wall layer thickness and stiffness in early childhood - RADIEL study six-year follow-up." Atherosclerosis. 59. Tielemans, M., E. Steegers, T. Voortman, V. Jaddoe, F. Rivadeneira, O. Franco and J. Kiefte-de Jong (2017). "Protein intake during pregnancy and offspring body composition at 6 years: the Generation R Study." European Journal of Nutrition 56(6): 2151-2160. 60. Wraw, C., C. Gale, G. Der and I. Deary (2017). "Maternal and offspring intelligence in relation to BMI across childhood and adolescence." Obesity Facts 10: 22. 61. Xu, R. Y., Y. Q. Zhou, X. M. Zhang, Y. P. Wan and X. Gao (2019). "A two-year study of parental obesity status and childhood obesity in China." Nutrition, Metabolism and Cardiovascular Diseases 29(3): 260-267. 62. Yeung, E. H., R. Sundaram, A. Ghassabian, Y. Xie and G. Buck Louis (2017). "Parental Obesity and Early Childhood Development." Pediatrics 139(2). 63. Yeung, H., M. Leff and K. E. Rhee (2017). "Effect of Exclusive Breastfeeding Among Overweight and Obese Mothers on Infant Weight-for-Length Percentile at 1 Year." Breastfeeding Medicine: The Official Journal of the Academy of Breastfeeding Medicine 12: 39-47. 64. Zhu, Y., S. F. Olsen, P. Mendola, T. I. Halldorsson, S. Rawal, S. N. Hinkle, E. H. Yeung, J. E. Chavarro, L. G. Grunnet, C. Granstrom, A. A. Bjerregaard, F. B. Hu and C. Zhang (2017). "Are you what your mother ate: Maternal artificially-sweetened beverages intake during pregnancy and offspring growth and obesity through age 7 years?" FASEB Journal. Conference: Experimental Biology 31(1 Supplement 1). 65. Liu J et al., Associations of maternal gestational weight gain with the risk of offspring obesity and body mass index Z scores beyond the mean, Annals of Epidemiology, https://doi.org/10.1016/j.annepidem.2019.01.007 66. Donkor HM, Grundt JH, Júlíusson PB, et al. Social and somatic determinants of underweight, overweight and obesity at 5 years of age: a Norwegian regional cohort study. BMJ Open 2017;0:e014548 67. Oostvogels et al. Does maternal pre-pregnancy overweight or obesity influence offspring's growth patterns from birth up to 7 years? The ABCD-study. Early Human Development 113 (2017) 62–70 68. Chiavaroli, V., J. G. B. Derraik, S. A. Hopkins, R. O. Rodrigues, W. S. Cutfield and P. L. Hofman (2017). "Greater maternal BMI early in pregnancy is associated with increased adiposity in 7-year-old offspring, but without adverse effects on metabolism." International Journal of Pediatric Endocrinology. Conference: 9th Biennial Scientific Meeting of the Asia Pacific Paediatric Endocrine Society, APPES and the 50th Annual Meeting of the Japanese Society for Pediatric Endocrinology, JSPE. Japan.(pagination). 69. Andres, A., M. L. Ruebel, R. A. Krukowski, T. M. Badger, K. Shankar and M. A. Cleves (2017). "First trimester maternal adiposity is associated with infant body fat at age 2 weeks: A longitudinal follow-up study." FASEB Journal. Conference: Experimental Biology 31(1 Supplement 1). 70. Ohlendorf, J. M., K. Robinson and M. Garnier-Villarreal (2019). "The impact of maternal BMI, gestational weight gain, and breastfeeding on early childhood weight: Analysis of a statewide WIC dataset." Preventive Medicine 118: 210-215. 71. Kaseva, N., M. Vaarasmaki, H. M. Matinolli, M. Sipola-Leppanen, M. Tikanmaki, K. Heinonen, A. Lano, D. Wolke, S. Andersson, M. R. Jarvelin, K. Raikkonen, J. G. Eriksson and E. Kajantie (2018). "Pre-pregnancy overweight or obesity and gestational diabetes as predictors of body composition in offspring twenty years later: Evidence from two birth cohort studies." International Journal of Obesity 42(4): 872-879. 72. Freemark, M. (2018). "Determinants of risk for childhood obesity." New England Journal of Medicine 379(14): 1371-1372. | |
