# Supplementary material for: The association between maternal body mass index and child obesity: A systematic review and meta-analysis
Source: PLoS Med. 2019 Jun 11;16(6):e1002817. doi: 10.1371/journal.pmed.1002817 (PMC6559702; doi:10.1371/journal.pmed.1002817)
Supplement: S4 Table — (DOCX) [file pmed.1002817.s014.docx]

# S4 Table: Screening: studies excluded due to duplicate cohort data

| **Child Ages^a^** | **1** | | **2** | | **3** | | **4** | | **5** | | **6** | **7** | | **8** | | **9** | | **10** | | **11** | | **12** | **13** | | **14** | **15** | **16** | **17** | **Reason for decision** |
| --- | --- | --- | --- | --- | --- | --- | --- | --- | --- | --- | --- | --- | --- | --- | --- | --- | --- | --- | --- | --- | --- | --- | --- | --- | --- | --- | --- | --- | --- |
| **Amsterdam Born Child and their Development (ABCD) Study** | | | | | | | | | | | | | | | | | | | | | | | | | | | | | |
| de Hoog ML, van Eijsden M, Stronks K, Gemke RJ, Vrijkotte TG. Overweight at age two years in a multi-ethnic cohort (ABCD study): the role of prenatal factors, birth outcomes and postnatal factors. BMC Public Health. 2011;11:611. |  | |  | |  | |  | |  | |  |  | |  | |  | |  | |  | |  |  | |  |  |  |  | Gademan *et al.* 2014[1] reported more BMI categories |
| Gademan MG, Vermeulen M, Oostvogels AJ, *et al.* Maternal prepregancy BMI and lipid profile during early pregnancy are independently associated with offspring's body composition at age 5-6 years: the ABCD study. PLoS ONE [Electronic Resource]. 2014;9:e94594. |  | |  | |  | |  | |  | |  |  | |  | |  | |  | |  | |  |  | |  |  |  |  |  |
| Harskamp-van Ginkel MW, London SJ, Magnus MC, Gademan MG, Vrijkotte TG. A Study on Mediation by Offspring BMI in the Association between Maternal Obesity and Child Respiratory Outcomes in the Amsterdam Born and Their Development Study Cohort. PLoS One. 2015;10:e0140641. |  | |  | |  | |  | |  | |  |  | |  | |  | |  | |  | |  |  | |  |  |  |  |  |
| Mesman I, Roseboom TJ, Bonsel GJ, Gemke RJ, van der Wal MF, Vrijkotte TG. Maternal pre-pregnancy body mass index explains infant's weight and BMI at 14 months: results from a multi-ethnic birth cohort study. Arch Dis Child. 2009;94:587-595. |  | |  | |  | |  | |  | |  |  | |  | |  | |  | |  | |  |  | |  |  |  |  |  |
| Mintjens S, Gemke RJBJ, van Poppel MNM, Vrijkotte TGM, Roseboom TJ, van Deutekom AW. Maternal Prepregnancy Overweight and Obesity Are Associated with Reduced Physical Fitness But Do Not Affect Physical Activity in Childhood: The Amsterdam Born Children and Their Development Study. Childhood Obesity. 2018;15(1):31-9.^d^ |  | |  | |  | |  | |  | |  |  | | ^b^ | | | |  | |  | |  |  | |  |  |  |  |  |
| Sirkka O, Vrijkotte T, Halberstadt J, Abrahamse-Berkeveld M, Hoekstra T, Seidell J, et al. Prospective associations of age at complementary feeding and exclusive breastfeeding duration with body mass index at 5-6 years within different risk groups. Pediatr Obes. 2018;13(8):522-9.^d^ |  | |  | |  | |  | | ^b^ | | |  | |  | |  | |  | |  | |  |  | |  |  |  |  |  |
| **Alaska Pregnancy Risk Assessment Monitoring System (PRAMS)** | | | | | | | | | | | | | | | | | | | | | | | | | | | | | |
| Wojcicki JM, Young MB, Perham-Hester KA, de Schweinitz P, Gessner BD. Risk factors for obesity at age 3 in Alaskan children, including the role of beverage consumption: results from Alaska PRAMS 2005-2006 and its three-year follow-up survey, CUBS, 2008-2009. PLoS ONE. 2015;10:e0118711. |  | |  | |  | |  | |  | |  |  | |  | |  | |  | |  | |  |  | |  |  |  |  |  |
| **Avon Longitudinal Study of Parents and Children (ALSPAC)** | | | | | | | | | | | | | | | | | | | | | | | | | | | | | |
| Reilly JJ, Armstrong J, Dorosty AR, *et al.* Early life risk factors for obesity in childhood: cohort study. BMJ. 2005;330:1357. |  | |  | |  | |  | |  | |  |  | |  | |  | |  | |  | |  |  | |  |  |  |  |  |
| Davey Smith G, Steer C, Leary S, Ness A. Is there an intrauterine influence on obesity? Evidence from parent child associations in the Avon Longitudinal Study of Parents and Children (ALSPAC). Arch Dis Child. 2007;92:876-880. |  | |  | |  | |  | |  | |  |  | |  | |  | |  | |  | |  |  | |  |  |  |  |  |
| **Bassett Mothers Health Project** | | | | | | | | | | | | | | | | | | | | | | | | | | | | | |
| Olson CM, Strawderman MS, Dennison BA. Maternal weight gain during pregnancy and child weight at age 3 years. Mat Child Health J. 2009;13:839-846. |  | |  | |  | |  | |  | |  |  | |  | |  | |  | |  | |  |  | |  |  |  |  |  |
| Olson CM, Demment MM, Carling SJ, Strawderman MS. Associations Between Mothers' and Their Children's Weights at 4 Years of Age. Child Obes. 2010;6:201-207. |  | |  | |  | |  | |  | |  |  | |  | |  | |  | |  | |  |  | |  |  |  |  |  |
| **Beginnings Study** | | | | | | | | | | | | | | | | | | | | | | | | | | | | | |
| Andres A, Hull HR, Shankar K, Casey PH, Cleves MA, Badger TM. Longitudinal body composition of children born to mothers with normal weight, overweight, and obesity. Obesity. 2015;23:1252-1258 |  | |  | |  | |  | |  | |  |  | |  | |  | |  | |  | |  |  | |  |  |  |  |  |
| **Child Health and Development Studies** | | | | | | | | | | | | | | | | | | | | | | | | | | | | | |
| Margerison-Zilko CE, Shrimali BP, Eskenazi B, Lahiff M, Lindquist AR, Abrams BF. Trimester of maternal gestational weight gain and offspring body weight at birth and age five. Mat Child Health J. 2012;16:1215-1223. |  | |  | |  | |  | |  | |  |  | |  | |  | |  | |  | |  |  | |  |  |  |  |  |
| **Cohort Study of Young Girls' Nutrition, Environment and Transitions (CYGNET)** | | | | | | | | | | | | | | | | | | | | | | | | | | | | | |
| Kubo A, Ferrara A, Laurent CA, *et al.* Associations between Maternal Pregravid Obesity and Gestational Diabetes and the Timing of Pubarche in Daughters. Am J Epidemiol. 2016;184:7-14. |  | |  | |  | |  | |  | | ^b^ | | | | |  | |  | |  | |  |  | |  |  |  |  |  |
| **Danish National Birth Cohort** | | | | | | | | | | | | | | | | | | | | | | | | | | | | | |
| Ajslev TA, Andersen CS, Gamborg M, Sorensen TI, Jess T. Childhood overweight after establishment of the gut microbiota: the role of delivery mode, pre-pregnancy weight and early administration of antibiotics. Int J Obes (Lond). 2011;35:522-529. |  | |  | |  | |  | |  | |  | ^c^ | |  | |  | |  | |  | |  |  | |  |  |  |  | Ajslev *et al.* 2011[2] reported more BMI categories and Morgen *et al.* 2017[3] reported multiple age groups |
| Ajslev TA, Andersen CS, Ingstrup KG, Nohr EA, Sørensen TIA. Maternal Postpartum Distress and Childhood Overweight. PLoS ONE. 2010;5:e11136. |  | |  | |  | |  | |  | |  |  | |  | |  | |  | |  | |  |  | |  |  |  |  |  |
| Andersen CS, Gamborg M, Sorensen TI, Nohr EA. Weight gain in different periods of pregnancy and offspring's body mass index at 7 years of age. Int Jf Pediatr Obes. 2011;6:e179-186. |  | |  | |  | |  | |  | |  |  | |  | |  | |  | |  | |  |  | |  |  |  |  |  |
| Morgen C, Angquist L, Baker J, Andersen A, Michaelsen K, SoRensen T. Prenatal risk factors infuencing childhood BMI and overweight independent of birth weight and infancy BMI - A path analysis within the Danish national birth cohort. Obesity Facts. 2017;10:21-22. |  | |  | |  | |  | |  | |  | ^c^ | |  | |  | |  | |  | |  |  | |  |  |  |  |  |
| Sorensen T, Ajslev TA, Angquist L, Morgen CS, Ciuchi IG, Davey Smith G. Comparison of associations of maternal peri-pregnancy and paternal anthropometrics with child anthropometrics from birth through age 7 y assessed in the Danish National Birth Cohort. Am J Clin Nutr. 2016;104:389-396. |  | |  | |  | |  | |  | |  |  | |  | |  | |  | |  | |  |  | |  |  |  |  |  |
| **Delaware Mother Baby Cohort** | | | | | | | | | | | | | | | | | | | | | | | | | | | | | |
| Ehrenthal DB, Maiden K, Rao A, *et al.* Independent relation of maternal prenatal factors to early childhood obesity in the offspring. Obstet Gynecol. 2013;121:115-121. |  | |  | |  | |  | |  | |  |  | |  | |  | |  | |  | |  |  | |  |  |  |  |  |
| **Early Childhood Longitudinal Study-Birth Cohort** | | | | | | | | | | | | | | | | | | | | | | | | | | | | | |
| Hinkle SN, Sharma AJ, Swan DW, Schieve LA, Ramakrishnan U, Stein AD. Excess gestational weight gain is associated with child adiposity among mothers with normal and overweight prepregnancy weight status. J Nutr. 2012;142:1851-1858. |  | |  | |  | |  | |  | |  |  | |  | |  | |  | |  | |  |  | |  |  |  |  | Hinkle *et al.* 2012[4] did not combine age groups Kitsantas *et al.* 2010[5] made more adjustments Weden *et al.* 2012[6] contains more than one cohort |
| Hinkle SN, Sharma AJ, Kim SY, Schieve LA. Maternal prepregnancy weight status and associations with children's development and disabilities at kindergarten. Int J Obes (2005). 2013;37:1344-1351. |  | |  | |  | |  | | ^b^ | | |  | |  | |  | |  | |  | |  |  | |  |  |  |  |  |
| Kitsantas P, Pawloski LR, Gaffney KF. Maternal prepregnancy body mass index in relation to Hispanic preschooler overweight/obesity. Eur J Pediatr. 2010;169:1361-1368. |  | |  | |  | |  | |  | |  |  | |  | |  | |  | |  | |  |  | |  |  |  |  |  |
| Kitsantas P, Gaffney KF. Risk profiles for overweight/obesity among preschoolers. Early Hum Dev. 2010;86:563-568. |  | |  | |  | |  | |  | |  |  | |  | |  | |  | |  | |  |  | |  |  |  |  |  |
| Weden MM, Brownell P, Rendall MS. Prenatal, Perinatal, Early Life, and Sociodemographic Factors Underlying Racial Differences in the Likelihood of High Body Mass Index in Early Childhood. Am J Public Health. 2012;102:2057-2067. |  | |  | |  | | ^b^ | | | |  |  | |  | |  | |  | |  | |  |  | |  |  |  |  |  |
| Flores G, Lin H. Factors predicting overweight in US kindergartners. Am J Clin Nutr. 2013;97:1178-1187. |  | |  | |  | |  | | ^b^ | | |  | |  | |  | |  | |  | |  |  | |  |  |  |  |  |
| Flores G, Lin H. Factors predicting severe childhood obesity in kindergarteners. Int J Obes (Lond). 2013;37:31-39. |  | |  | |  | |  | | ^b^ | | |  | |  | |  | |  | |  | |  |  | |  |  |  |  |  |
| **EDEN Mother–Child Cohort** | | | | | | | | | | | | | | | | | | | | | | | | | | | | | |
| Jacota M, Forhan A, Saldanha-Gomes C, Charles MA, Heude B, for the EMCCSG. Maternal weight prior and during pregnancy and offspring's BMI and adiposity at 5–6 years in the EDEN mother–child cohort. Pediatr Obes. 2016 |  | |  | |  | |  | | ^b^ | | |  | |  | |  | |  | |  | |  |  | |  |  |  |  |  |
| **Exeter Family Study of Childhood Health (EFSOCH)** | | | | | | | | | | | | | | | | | | | | | | | | | | | | | |
| Knight B, Shields BM, Hill A, Powell RJ, Wright D, Hattersley AT. The impact of maternal glycemia and obesity on early postnatal growth in a nondiabetic Caucasian population. Diab Care. 2007;30:777-783. |  | |  | |  | |  | |  | |  |  | |  | |  | |  | |  | |  |  | |  |  |  |  |  |
| **Exploring Perinatal Outcomes among Children Study (EPOCH)** | | | | | | | | | | | | | | | | | | | | | | | | | | | | | |
| Kaar JL, Crume T, Brinton JT, Bischoff KJ, McDuffie R, Dabelea D. Maternal obesity, gestational weight gain, and offspring adiposity: the exploring perinatal outcomes among children study. J Pediatr. 2014;165:509-515. |  | |  | |  | | 10.5 | |  | | ^b^ | | | | | | | | | | | | | |  |  |  |  | Kaar *et al.* 2014[7] reported frequency data |
| Crume TL, Ogden L, Daniels S, Hamman RF, Norris JM, Dabelea D. The impact of in utero exposure to diabetes on childhood body mass index growth trajectories: the EPOCH study. J Pediatr. 2011;158:941-946. |  | | ^b^ | | | | ^b^ | | | | | ^b^ | | | | | | ^b^ | | | | | | |  |  |  |  |  |
| Bellatorre A, Scherzinger A, Stamm E, Martinez M, Ringham B, Dabelea D. Fetal Overnutrition and Adolescent Hepatic Fat Fraction: the Exploring Perinatal Outcomes in Children Study. J Pediatr. 2018;192:165-70.e1.^d^ |  |  | |  | |  | |  | |  | | |  | |  | |  | |  | |  |  | |  |  |  |  |  |  |
| **Generation R Study** | | | | | | | | | | | | | | | | | | | | | | | | | | | | | |
| Gaillard R, Steegers EA, Duijts L, *et al.* Childhood cardiometabolic outcomes of maternal obesity during pregnancy: the Generation R Study. Hypertension. 2014;63:683-691. |  | |  | |  | |  | |  | | ^c^ |  | |  | |  | |  | |  | |  |  | |  |  |  |  | Durmus *et al.* 2012[8] did not combine age groups |
| Durmus B, Arends LR, Ay L, *et al.* Parental anthropometrics, early growth and the risk of overweight in pre-school children: the Generation R Study. Pediatric Obesity. 2012;8:339-350. |  | |  | |  | |  | |  | |  |  | |  | |  | |  | |  | |  |  | |  |  |  |  |  |
| Heppe DH, Kiefte-de Jong JC, Durmus B, *et al.* Parental, fetal, and infant risk factors for preschool overweight: the Generation R Study. Pediatric Research. 2013;73:120-127. |  | |  | | ^b^ | | | |  | |  |  | |  | |  | |  | |  | |  |  | |  |  |  |  |  |
| Toemen L, Gishti O, van Osch-Gevers L, *et al.* Maternal obesity, gestational weight gain and childhood cardiac outcomes: role of childhood body mass index. Int J Obes (Lond). 2016;40:1070-1078. |  | |  | |  | |  | |  | | ^c^ |  | |  | |  | |  | |  | |  |  | |  |  |  |  |  |
| Jharap VV, Santos S, Steegers EAP, Jaddoe VWV, Gaillard R. Associations of maternal obesity and excessive weight gain during pregnancy with subcutaneous fat mass in infancy. Early Human Dev. 2017;108:23-28. |  | |  | |  | |  | |  | |  |  | |  | |  | |  | |  | |  |  | |  |  |  |  |  |
| **Greek Childhood Obesity (GRECO) Study** | | | | | | | | | | | | | | | | | | | | | | | | | | | | | |
| Risvas G, Papaioannou I, Panagiotakos DB, Farajian P, Bountziouka V, Zampelas A. Perinatal and family factors associated with preadolescence overweight/obesity in Greece: the GRECO study. J Epidemiol Glob Health. 2012;2:145-153. |  | |  | |  | |  | |  | |  |  | |  | |  | | ^b^ | | | | |  | |  |  |  |  |  |
| **Growing Up Today Study** | | | | | | | | | | | | | | | | | | | | | | | | | | | | | |
| Oken E, Rifas-Shiman SL, Field AE, Frazier AL, Gillman MW. Maternal gestational weight gain and offspring weight in adolescence. Obstet Gynecol. 2008;112:999-1006. |  | |  | |  | |  | |  | |  |  | |  | | ^b^ | | | | | | | | | |  |  |  |  |
| **Healthy Beginnings Trial** | | | | | | | | | | | | | | | | | | | | | | | | | | | | | |
| Wen LM, Baur LA, Rissel C, Xu H, Simpson JM. Correlates of body mass index and overweight and obesity of children aged 2 years: findings from the healthy beginnings trial. Obesity. 2014;22:1723-1730. |  | |  | |  | |  | |  | |  |  | |  | |  | |  | |  | |  |  | |  |  |  |  |  |
| **Healthy Growth Study** | | | | | | | | | | | | | | | | | | | | | | | | | | | | | |
| Birbilis M, Moschonis G, Mougios V, Manios Y, Healthy Growth Study g. Obesity in adolescence is associated with perinatal risk factors, parental BMI and sociodemographic characteristics. Eur J Clin Nutr. 2013;67:115-121. |  | |  | |  | |  | |  | |  |  | |  | | ^b^ | | | | | | | | |  |  |  |  |  |
| **Maternal Health Practices and Child Development Project** | | | | | | | | | | | | | | | | | | | | | | | | | | | | | |
| Diesel JC, Eckhardt CL, Day NL, Brooks MM, Arslanian SA, Bodnar LM. Is gestational weight gain associated with offspring obesity at 36 months? Pediatr Obesity. 2014;10:305-310. |  | |  | |  | |  | |  | |  |  | |  | |  | |  | |  | |  |  | |  |  |  |  |  |
| **Mater-University Study of Pregnancy** | | | | | | | | | | | | | | | | | | | | | | | | | | | | | |
| O'Callaghan MJ, Williams GM, Andersen MJ, Bor W, Najman JM. Prediction of obesity in children at 5 years: a cohort study. J Paediatr Child Health. 1997;33:311-316. |  | |  | |  | |  | | ^c^ | |  |  | |  | |  | |  | |  | |  |  | |  |  |  |  |  |
| Mamun AA, Lawlor DA, O'Callaghan MJ, Williams GM, Najman JM. Family and early life factors associated with changes in overweight status between ages 5 and 14 years: findings from the Mater University Study of Pregnancy and its outcomes. Int J Obes (Lond). 2005;29:475-482. |  | |  | |  | |  | | ^c^ | |  |  | |  | |  | |  | |  | |  |  | |  |  |  |  |  |
| **Millennium Cohort Study** | | | | | | | | | | | | | | | | | | | | | | | | | | | | | |
| Basatemur E, Gardiner J, Williams C, Melhuish E, Barnes J, Sutcliffe A. Maternal prepregnancy BMI and child cognition: a longitudinal cohort study. Pediatrics. 2013;131:56-63. |  | |  | |  | |  | |  | |  |  | |  | |  | |  | |  | |  |  | |  |  |  |  | Weng *et al.* 2013[9] reported more BMI categories and made more adjustments |
| Hawkins SS, Cole TJ, Law C. An ecological systems approach to examining risk factors for early childhood overweight: findings from the UK Millennium Cohort Study. J Epidemiol Comm Health. 2009;63:147-155. |  | |  | |  | |  | |  | |  |  | |  | |  | |  | |  | |  |  | |  |  |  |  |  |
| Weng SF, Redsell SA, Nathan D, Swift JA, Yang M, Glazebrook C. Estimating overweight risk in childhood from predictors during infancy. Pediatrics. 2013;132:e414-421. |  | |  | |  | |  | |  | |  |  | |  | |  | |  | |  | |  |  | |  |  |  |  |  |
| Ramasubramanian L, Lane S, Rahman A. The association between maternal serious psychological distress and child obesity at 3 years: a cross-sectional analysis of the UK Millennium Cohort Data. Child Care Health Dev. 2013;39:134-140. |  | |  | |  | |  | |  | |  |  | |  | |  | |  | |  | |  |  | |  |  |  |  |  |
| Massion S, Wickham S, Pearce A, Barr B, Law C, Taylor-Robinson D. Exploring the impact of early life factors on inequalities in risk of overweight in UK children: findings from the UK Millennium Cohort Study. Arch Dis Child. 2016. |  | |  | |  | |  | |  | |  |  | |  | |  | |  | |  | |  |  | |  |  |  |  |  |
| **National Collaborative Perinatal Project** | | | | | | | | | | | | | | | | | | | | | | | | | | | | | |
| Wrotniak BH, Shults J, Butts S, Stettler N. Gestational weight gain and risk of overweight in the offspring at age 7 y in a multicenter, multiethnic cohort study. Am J Clin Nutr. 2008;87:1818-1824. |  | |  | |  | |  | |  | |  |  | |  | |  | |  | |  | |  |  | |  |  |  |  |  |
| **National Longitudinal Survey of Youth (NLSY)** | | | | | | | | | | | | | | | | | | | | | | | | | | | | | |
| Li C, Kaur H, Choi WS, Huang TT, Lee RE, Ahluwalia JS. Additive interactions of maternal prepregnancy BMI and breast-feeding on childhood overweight. Obes Res. 2005;13:362-371. |  | | ^b^ | | | | | | | | | ^b^ | | | | | | | | ^b^ | | | | | |  |  |  | Salsberry *et al.* 2005[10] reported frequency data Weden *et al.* 2012[6] contains more than one cohort |
| Reagan PB, Salsberry PJ, Fang MZ, Gardner WP, Pajer K. African-American/white differences in the age of menarche: accounting for the difference. Soc Sci Med (1982). 2012;75:1263-1270 |  | |  | |  | |  | |  | |  |  | |  | |  | |  | |  | |  |  | |  |  |  |  |  |
| Weden MM, Brownell P, Rendall MS. Prenatal, Perinatal, Early Life, and Sociodemographic Factors Underlying Racial Differences in the Likelihood of High Body Mass Index in Early Childhood. Am J Public Health. 2012;102:2057-2067. |  | |  | |  | | ^b^ | | | |  |  | |  | |  | |  | |  | |  |  | |  |  |  |  |  |
| Salsberry PJ, Reagan PB. Dynamics of early childhood overweight. Pediatrics. 2005;116:1329-1338. |  | | ^b^ | | | | ^b^ | | | | ^b^ | | |  | |  | |  | |  | |  |  | |  |  |  |  |  |
| Salsberry PJ, Reagan PB. Taking the long view: the prenatal environment and early adolescent overweight. Res Nurs Health. 2007;30:297-307. |  | |  | |  | |  | |  | |  |  | |  | |  | |  | |  | | ^b^ | | |  |  |  |  |  |
| Robinson CA, Cohen AK, Rehkopf DH, *et al.* Pregnancy and post-delivery maternal weight changes and overweight in preschool children. Prev Med. 2014;60:77-82. |  | |  | |  | | ^b^ | | | |  |  | |  | |  | |  | |  | |  |  | |  |  |  |  |  |
| **Northern Finland Birth Cohort** | | | | | | | | | | | | | | | | | | | | | | | | | | | | | |
| Jaaskelainen A, Pussinen J, Nuutinen O, *et al.* Intergenerational transmission of overweight among Finnish adolescents and their parents: a 16-year follow-up study. Int J Obes (Lond.). 2011;35:1289-1294. |  | |  | |  | |  | |  | |  |  | |  | |  | |  | |  | |  |  | |  |  |  |  | Laitinen *et al.* 2012[11] had a larger sample size |
| Laitinen J, Jaaskelainen A, Hartikainen AL, *et al.* Maternal weight gain during the first half of pregnancy and offspring obesity at 16 years: a prospective cohort study. BJOG. 2012;119:716-723. |  | |  | |  | |  | |  | |  |  | |  | |  | |  | |  | |  |  | |  |  |  |  |  |
| **Norwegian Mother and Child Cohort Study** | | | | | | | | | | | | | | | | | | | | | | | | | | | | | |
| Fleten C, Nystad W, Stigum H, *et al.* Parent-offspring body mass index associations in the Norwegian Mother and Child Cohort Study: a family-based approach to studying the role of the intrauterine environment in childhood adiposity. Am J Epidemiol. 2012;176:83-92. |  | |  | | ^c^ | |  | |  | |  |  | |  | |  | |  | |  | |  |  | |  |  |  |  |  |
| Stamnes Kopp UM, Dahl-Jorgensen K, Stigum H, Frost Andersen L, Naess O, Nystad W. The associations between maternal pre-pregnancy body mass index or gestational weight change during pregnancy and body mass index of the child at 3 years of age. Int J Obes (Lond). 2012;36:1325-1331. |  | |  | | ^c^ | |  | |  | |  |  | |  | |  | |  | |  | |  |  | |  |  |  |  |  |
| **Population-based Premarital and Perinatal Health Care System** | | | | | | | | | | | | | | | | | | | | | | | | | | | | | |
| Guo L, Liu J, Ye R, Liu J, Zhuang Z, Ren A. Gestational Weight Gain and Overweight in Children Aged 3-6 Years. J Epidemiol. 2015;25:536-543. |  | |  | | ^b^ | | | | | | |  | |  | |  | |  | |  | |  |  | |  |  |  |  |  |
| **Pregnancy Infection and Nutrition and Postpartum Studies** | | | | | | | | | | | | | | | | | | | | | | | | | | | | | |
| Deierlein AL, Siega-Riz AM, Chantala K, Herring AH. The association between maternal glucose concentration and child BMI at age 3 years. Diabetes Care. 2011;34:480-484. |  | |  | |  | |  | |  | |  |  | |  | |  | |  | |  | |  |  | |  |  |  |  |  |
| **Prenatal Exposures and Preeclampsia Prevention** | | | | | | | | | | | | | | | | | | | | | | | | | | | | | |
| Tan HC, Roberts J, Catov J, Krishnamurthy R, Shypailo R, Bacha F. Mother's pre-pregnancy BMI is an important determinant of adverse cardiometabolic risk in childhood. Pediatr Diabetes. 2015;16:419-426. |  | |  | |  | |  | |  | |  |  | |  | |  | |  | |  | |  |  | |  |  |  |  |  |
| **Project Viva** | | | | | | | | | | | | | | | | | | | | | | | | | | | | | |
| Oken E, Kleinman KP, Belfort MB, Hammitt JK, Gillman MW. Associations of gestational weight gain with short- and longer-term maternal and child health outcomes. Am J Epidemiol. 2009;170:173-180. |  | |  | |  | |  | |  | |  |  | |  | |  | |  | |  | |  |  | |  |  |  |  | Gillman *et al.* 2008[12] data was more suitable for meta-analysis |
| Gillman MW, Rifas-Shiman SL, Kleinman K, Oken E, Rich-Edwards JW, Taveras EM. Developmental origins of childhood overweight: potential public health impact. Obesity. 2008;16:1651-1656 |  | |  | |  | |  | |  | |  |  | |  | |  | |  | |  | |  |  | |  |  |  |  |  |
| **Kaiser Permanente Northern California** | | | | | | | | | | | | | | | | | | | | | | | | | | | | | |
| Pham MT, Brubaker K, Pruett K, Caughey AB. Risk of childhood obesity in the toddler offspring of mothers with gestational diabetes. Obstet Gynecol. 2013;121:976-982. |  | | ^b^ | | | | | |  | |  |  | |  | |  | |  | |  | |  |  | |  |  |  |  |  |
| **Rhea Pregnancy Cohort** | | | | | | | | | | | | | | | | | | | | | | | | | | | | | |
| Daraki V, Georgiou V, Papavasiliou S, *et al.* Metabolic profile in early pregnancy is associated with offspring adiposity at 4 years of age: the Rhea Pregnancy Cohort Crete, Greece. PLoS ONE. 2015;10:e0126327. |  | |  | |  | |  | |  | |  |  | |  | |  | |  | |  | |  |  | |  |  |  |  |  |
| **Population: Midwest USA** | | | | | | | | | | | | | | | | | | | | | | | | | | | | | |
| Eisenman JC, Sarzynski MA, Tucker J, Heelan KA. Maternal prepregnancy overweight and offspring fatness and blood pressure: role of physical activity. Pediatr Exerc Sci. 2010;22:369-378. |  | |  | |  | |  | |  | |  |  | |  | |  | |  | |  | |  |  | |  |  |  |  |  |
| Rooney BL, Mathiason MA, Schauberger CW. Predictors of obesity in childhood, adolescence, and adulthood in a birth cohort. Matern Child Health J. 2011;15:1166-1175. |  | |  | |  | | ^b^ | | | |  |  | |  | | ^b^ | | | | | | | | | |  |  |  |  |
| **Special Supplemental Nutrition Program for Women, Infants, and Children** | | | | | | | | | | | | | | | | | | | | | | | | | | | | | |
| Whitaker RC. Predicting preschooler obesity at birth: the role of maternal obesity in early pregnancy. Pediatrics. 2004;114:e29-36. |  | |  | |  | |  | |  | |  |  | |  | |  | |  | |  | |  |  | |  |  |  |  |  |
| **Steps to Health Development (STEPS) Study** | | | | | | | | | | | | | | | | | | | | | | | | | | | | | |
| Makela J, Lagstrom H, Kaljonen A, Simell O, Niinikoski H. Hyperglycemia and lower diet quality in pregnant overweight women and increased infant size at birth and at 13 months of age-STEPS study. Early Human Dev. 2013;89:439-444. |  | |  | |  | |  | |  | |  |  | |  | |  | |  | |  | |  |  | |  |  |  |  |  |
| **Universiti Sains Malaysia (USM) Pregnancy Cohort** | | | | | | | | | | | | | | | | | | | | | | | | | | | | | |
| Zalbahar N, Jan Mohamed HJ, Loy SL, Najman J, McIntyre HD, Mamun A. Association of parental body mass index before pregnancy on infant growth and body composition: Evidence from a pregnancy cohort study in Malaysia. Obes Res Clin Pract. 2015. |  | |  | |  | |  | |  | |  |  | |  | |  | |  | |  | |  |  | |  |  |  |  |  |
| **Wisconsin Nutrition and Growth Study** | | | | | | | | | | | | | | | | | | | | | | | | | | | | | |
| Lindberg SM, Adams AK, Prince RJ. Early Predictors of Obesity and Cardiovascular Risk Among American Indian Children. Mat Child Health J. 2012;16:1879-1886. |  | |  | |  | |  | | ^b^ | | | | | | |  | |  | |  | |  |  | |  |  |  |  |  |
| **Birth Cohort Study of Chinese Infants in Shenyang, Wuhan and Guangzhou** | | | | | | | | | | | | | | | | | | | | | | | | | | | | | |
| Mei H, Guo B, Yin B, *et al.* Interactive Effects of Early Exclusive Breastfeeding and Pre-Pregnancy Maternal Weight Status on Young Children’s BMI – A Chinese Birth Cohort. PLoS ONE. 2015;10:e0144357 |  | |  | |  | |  | |  | |  |  | |  | |  | |  | |  | |  |  | |  |  |  |  | Zhang *et al.* 2013[13] reported more BMI categories |
| Zhang J, Himes JH, Guo Y, *et al.* Birth weight, growth and feeding pattern in early infancy predict overweight/obesity status at two years of age: a birth cohort study of Chinese infants. PLoS ONE. 2013;8:e64542. |  | |  | |  | |  | |  | |  |  | |  | |  | |  | |  | |  |  | |  |  |  |  |  |
| Mei H, Guo S, Lu H, Pan Y, Mei W, Zhang B, et al. Impact of parental weight status on children’s body mass index in early life: evidence from a Chinese cohort. BMJ Open. 2018;8(6):e018755.^d^ |  | |  | |  | |  | |  | |  |  | |  | |  | |  | |  | |  |  | |  |  |  |  |  |
| **Infant Growth Study** | | | | | | | | | | | | | | | | | | | | | | | | | | | | | |
| Berkowitz RI, Stallings VA, Maislin G, Stunkard AJ. Growth of children at high risk of obesity during the first 6 y of life: implications for prevention. Am J Clin Nutr. 2005;81:140-146. |  | |  | |  | |  | |  | |  |  | |  | |  | |  | |  | |  |  | |  |  |  |  |  |
| **Kaiser Permanente Southern California** | | | | | | | | | | | | | | | | | | | | | | | | | | | | | |
| Bider-Canfield Z, Martinez MP, Wang X, *et al.* Maternal obesity, gestational diabetes, breastfeeding and childhood overweight at age 2 years. Pediatr Obesity. 2017;12:171-178. |  | |  | |  | |  | |  | |  |  | |  | |  | |  | |  | |  |  | |  |  |  |  |  |
| **Longitudinal Study of Pregnancy Outcomes and Childhood Psychomotor Development** | | | | | | | | | | | | | | | | | | | | | | | | | | | | | |
| Janjua NZ, Mahmood B, Islam MA, Goldenberg RL. Maternal and early childhood risk factors for overweight and obesity among low-income predominantly black children at age five years: A prospective cohort study. J Obes. 2012. |  | |  | |  | |  | |  | |  |  | |  | |  | |  | |  | |  |  | |  |  |  |  |  |
| **Population: 16 Schools in Sri Lanka** | | | | | | | | | | | | | | | | | | | | | | | | | | | | | |
| Rathnayake KM, Satchithananthan A, Mahamithawa S, Jayawardena R. Early life predictors of preschool overweight and obesity: a case-control study in Sri Lanka. BMC Public Health. 2013;13:994. |  | |  | | ^b^ | | | | | |  |  | |  | |  | |  | |  | |  |  | |  |  |  |  |  |
| **Population: Santiago Public Kindergartens, Chile** | | | | | | | | | | | | | | | | | | | | | | | | | | | | | |
| Rios-Castillo I, Cerezo S, Corvalan C, Martinez M, Kain J. Risk factors during the prenatal period and the first year of life associated with overweight in 7-year-old low-income Chilean children. Mat Child Nutr. 2015;11:595-605. |  | |  | |  | |  | |  | |  |  | |  | |  | |  | |  | |  |  | |  |  |  |  |  |
| **Population: MetroHealth Medical Centre, Cleveland, Ohio, US** | | | | | | | | | | | | | | | | | | | | | | | | | | | | | |
| Catalano PM, Farrell K, Thomas A, *et al.* Perinatal risk factors for childhood obesity and metabolic dysregulation. Am J Clin Nutr. 2009;90:1303-1313. |  | |  | |  | |  | |  | |  | ^b^ | | | | | | | |  | |  |  | |  |  |  |  |  |
| **Population: Tianjin, Northern China** | | | | | | | | | | | | | | | | | | | | | | | | | | | | | |
| Leng J, Li W, Zhang S, *et al.* GDM Women's Pre-Pregnancy Overweight/Obesity and Gestational Weight Gain on Offspring Overweight Status. PLoS ONE. 2015;10:e0129536. | ^b, c^ | | | | | | | | | |  |  | |  | |  | |  | |  | |  |  | |  |  |  |  |  |
| Li N, Liu E, Guo J, *et al.* Maternal prepregnancy body mass index and gestational weight gain on offspring overweight in early infancy. PLoS ONE. 2013;8:e77809. | ^c^ | |  | |  | |  | |  | |  |  | |  | |  | |  | |  | |  |  | |  |  |  |  |  |
| Wang J, Liu E, Wang Y, Qiao Y, Zhang T, Li B, et al. Association of early pregnancy body mass index and children's birth weight with risk of being overweight in childhood. American Journal of Human Biology. 2018;30(5):e23174.^d^ |  | |  | |  | |  | |  | |  |  | |  | |  | |  | |  | |  |  | |  |  |  |  |  |
| **Population: Zhejiang, China** | | | | | | | | | | | | | | | | | | | | | | | | | | | | | |
| Jin WY, Lv Y, Bao Y, *et al.* Independent and combined effects of maternal prepregnancy body mass index and gestational weight gain on offspring growth at 0-3 years of age. BioMed Res Int. 2016. |  | |  | |  | |  | |  | |  |  | |  | |  | |  | |  | |  |  | |  |  |  |  |  |
| **Family Atherosclerosis Monitoring in Early Life (FAMILY) Cohort** | | | | | | | | | | | | | | | | | | | | | | | | | | | | | |
| Li A, Teo KK, Morrison KM, *et al.* A genetic link between prepregnancy body mass index, postpartum weight retention, and offspring weight in early childhood. Obesity. 2017;25:236-243. | ^b^ | | | | | | | | | |  |  | |  | |  | |  | |  | |  |  | |  |  |  |  |  |
| **Western Australia Pregnancy Cohort Study** | | | | | | | | | | | | | | | | | | | | | | | | | | | | | |
| Rath SR, Marsh JA, Newnham JP, *et al.* Parental pre-pregnancy BMI is a dominant early-life risk factor influencing BMI of offspring in adulthood. Obes Sci Pract. 2016;2:48-57. |  | |  | |  | |  | |  | |  |  | |  | |  | |  | |  | |  |  | |  |  |  |  |  |
| **Collaborative Perinatal Project** | | | | | | | | | | | | | | | | | | | | | | | | | | | | | |
| Terry MB, Wei Y, Esserman D, McKeague IW, Susser E. Pre- and postnatal determinants of childhood body size: cohort and sibling analyses. J Dev Orig Health Dis. 2011;2:99-111. |  | |  | |  | |  | |  | |  |  | |  | |  | |  | |  | |  |  | |  |  |  |  |  |
| **ToyBox Study** | | | | | | | | | | | | | | | | | | | | | | | | | | | | | |
| Androutsos O, Moschonis G, Ierodiakonou D, Karatzi K, De Bourdeaudhuij I, et al. Perinatal and lifestyle factors mediate the association between maternal education and preschool children's weight status: the ToyBox study. Nutrition. 2018;48: 6-12.^d^ |  | |  | |  | | ^b^ | | | | |  | |  | |  | |  | |  | |  |  | |  |  |  |  |  |
| **Growing Up in Singapore Towards healthy Outcomes (GUSTO) study** | | | | | | | | | | | | | | | | | | | | | | | | | | | | | |
| Aris IM, Bernard JY, Chen LW, Tint MT, Pang WW, et al. Modifiable risk factors in the first 1000 days for subsequent risk of childhood overweight in an Asian cohort: significance of parental overweight status. International Journal of Obesity. 2018;42: 44.^d^ |  | |  | |  | |  | |  | |  |  | |  | |  | |  | |  | |  |  | |  |  |  |  |  |
| **Canadian Healthy Infant Longitudinal Development (CHILD) study** | | | | | | | | | | | | | | | | | | | | | | | | | | | | | |
| Bridgman SL, Azad MB, Persaud RR, Chari RS, Becker AB, et al. Impact of maternal pre‐pregnancy overweight on infant overweight at 1 year of age: associations and sexspecific differences. Pediatric Obesity. 2018;13: 579-589.^d^ |  | |  | |  | |  | |  | |  |  | |  | |  | |  | |  | |  |  | |  |  |  |  |  |
| **Population: Fukuroi, Japan** | | | | | | | | | | | | | | | | | | | | | | | | | | | | | |
| Fujita Y, Kouda K, Nakamura H, Iki M. Relationship Between Maternal Pre-pregnancy Weight and Offspring Weight Strengthens as Children Develop: A Population-Based Retrospective Cohort Study. Journal of Epidemiology. 2018; JE20170137.^d^ |  | |  | |  | |  | |  | |  |  | |  | |  | |  | |  | |  |  | |  |  |  |  |  |
| **Growth and Feeding during Infancy and Early Childhood in Aragon (CALINA) study** | | | | | | | | | | | | | | | | | | | | | | | | | | | | | |
| Iguacel I, Escartín L, Fernández-Alvira JM, Iglesia I, Labayen I, et al. Early life risk factors and their cumulative effects as predictors of overweight in Spanish children. International Journal of Public Health 2018:63: 501-512.^d^ |  | |  | |  | |  | |  | |  |  | |  | |  | |  | |  | |  |  | |  |  |  |  | Iguacel *et al.* 2018[14] reported maternal BMI categories |
| Iguacel I, Fernandez-Alvira JM, Labayen I, Moreno LA, Samper MP, Rodriguez G. Social vulnerabilities as determinants of overweight in 2-, 4- and 6-year-old Spanish children. European journal of public health. 2018;28(2):289-95.^d^ |  | |  | |  | |  | |  | |  |  | |  | |  | |  | |  | |  |  | |  |  |  |  |  |
| **University of California, San Francisco Medical Center and San Francisco General Hospital** | | | | | | | | | | | | | | | | | | | | | | | | | | | | | |
| Kjaer TW, Faurholt-Jepsen D, Medrano R, Elwan D, Mehta K, et al. Higher birthweight and maternal pre-pregnancy BMI persist with obesity association at age 9 in high risk Latino children. Journal of Immigrant and Minority Health. 2019;1-9.^d^ |  | |  | |  | |  | |  | |  |  | |  | |  | |  | |  | |  |  | |  |  |  |  |  |
| **Boston Birth Cohort** | | | | | | | | | | | | | | | | | | | | | | | | | | | | | |
| Mao G, Nachman RM, Sun Q, Zhang X, Koehler K, et al. Individual and Joint Effects of Early-Life Ambient PM 2.5 Exposure and Maternal Prepregnancy Obesity on Childhood Overweight or Obesity. Environmental Health Perspectives. 2017;125: 067005.^d^ |  | | ^b^ | | | | | | | | | | | | | | |  | |  | |  |  | |  |  |  |  |  |
| **STORK Groruddalen, Oslo** | | | | | | | | | | | | | | | | | | | | | | | | | | | | | |
| Toftemo I, Jenum AK, Lagerløv P, Júlίusson PB, Falk RS, et al. Contrasting patterns of overweight and thinness among preschool children of different ethnic groups in Norway, and relations with maternal and early life factors. BMC Public Health. 2018; 1056.^d^ |  | |  | |  | | ^b^ | | | |  |  | |  | |  | |  | |  | |  |  | |  |  |  |  |  |
| **Population: Uppsala and Orebro, Sweden** | | | | | | | | | | | | | | | | | | | | | | | | | | | | | |
| Wallby T, Lagerberg D, Magnusson M. Relationship between breastfeeding and early childhood obesity: Results of a prospective longitudinal study from birth to 4 years. Breastfeeding Medicine. 2017; 12: 48-53.^d^ |  | |  | |  | |  | |  | |  |  | |  | |  | |  | |  | |  |  | |  |  |  |  |  |
| **Population: Affiliated Hospital of Jining Medical University, China** | | | | | | | | | | | | | | | | | | | | | | | | | | | | | |
| Zhang W, Niu F, Ren X. Association of maternal pre‐pregnancy body mass index and gestational weight gain with Chinese infant growth. Journal of Paediatrics and Child Health. 2018.^d^ |  | |  | |  | |  | |  | |  |  | |  | |  | |  | |  | |  |  | |  |  |  |  |  |
| **Melbourne Infant Feeding Activity and Nutrition Trial (InFANT) Program** | | | | | | | | | | | | | | | | | | | | | | | | | | | | | |
| Zheng M, Bowe SJ, Hesketh KD, Bolton K, Laws R, et al. Relative effects of postnatal rapid growth and maternal factors on early childhood growth trajectories. Paediatric and Perinatal Epidemiology. 2019.^d^ |  | |  | |  | |  | |  | |  |  | |  | |  | |  | |  | |  |  | |  |  |  |  |  |

Footnote:

^a^ Ages from the studies included in the systematic review meta-analysis and narrative analysis are highlighted in green and excluded studies and ages are highlighted in red.

^b^ Coloured cells are merged where data was reported as a combined age group.

^c^ Where the same age group for a cohort is highlighted in green more than once, the data are included in different analyses.

^d^ Studies identified from a search update in March 2019.

**References:**

1. Gademan MG, Vermeulen M, Oostvogels AJ, Roseboom TJ, Visscher TL, van Eijsden M, et al. Maternal prepregancy BMI and lipid profile during early pregnancy are independently associated with offspring's body composition at age 5-6 years: the ABCD study. PLoS ONE. 2014;9(4):e94594.

2. Ajslev TA, Andersen CS, Gamborg M, Sorensen TI, Jess T. Childhood overweight after establishment of the gut microbiota: the role of delivery mode, pre-pregnancy weight and early administration of antibiotics. Int J Obes (Lond). 2011;35(4):522-9.

3. Morgen C, Angquist L, Baker J, Andersen A, Michaelsen K, SoRensen T. Prenatal risk factors infuencing childhood BMI and overweight independent of birth weight and infancy BMI - A path analysis within the Danish national birth cohort. Obesity Facts. 2017;10:21-2.

4. Hinkle SN, Sharma AJ, Swan DW, Schieve LA, Ramakrishnan U, Stein AD. Excess gestational weight gain is associated with child adiposity among mothers with normal and overweight prepregnancy weight status. J Nutr. 2012;142(10):1851-8.

5. Kitsantas P, Pawloski LR, Gaffney KF. Maternal prepregnancy body mass index in relation to Hispanic preschooler overweight/obesity. Eur J Pediatr. 2010;169(11):1361-8.

6. Weden MM, Brownell P, Rendall MS. Prenatal, perinatal, early life, and sociodemographic factors underlying racial differences in the likelihood of high body mass index in early childhood. Am J Public Health. 2012;102(11):2057-67.

7. Kaar JL, Crume T, Brinton JT, Bischoff KJ, McDuffie R, Dabelea D. Maternal obesity, gestational weight gain, and offspring adiposity: the exploring perinatal outcomes among children study. J Pediatr. 2014;165(3):509-15.

8. Durmus B, Arends LR, Ay L, Hokken-Koelega AC, Raat H, Hofman A, et al. Parental anthropometrics, early growth and the risk of overweight in pre-school children: the Generation R Study. Pediatr Obes. 2012;8(5):339-50.

9. Weng SF, Redsell SA, Nathan D, Swift JA, Yang M, Glazebrook C. Estimating overweight risk in childhood from predictors during infancy. Pediatrics. 2013;132(2):e414-21.

10. Salsberry PJ, Reagan PB. Dynamics of early childhood overweight. Pediatrics. 2005;116(6):1329-38.

11. Laitinen J, Jaaskelainen A, Hartikainen AL, Sovio U, Vaarasmaki M, Pouta A, et al. Maternal weight gain during the first half of pregnancy and offspring obesity at 16 years: a prospective cohort study. Bjog. 2012;119(6):716-23.

12. Gillman MW, Rifas-Shiman SL, Kleinman K, Oken E, Rich-Edwards JW, Taveras EM. Developmental origins of childhood overweight: potential public health impact. Obesity (Silver Spring). 2008;16(7):1651-6.

13. Zhang J, Himes JH, Guo Y, Jiang J, Yang L, Lu Q, et al. Birth weight, growth and feeding pattern in early infancy predict overweight/obesity status at two years of age: a birth cohort study of Chinese infants. PLoS ONE. 2013;8(6):e64542.

14. Iguacel I, Escartín L, Fernández-Alvira JM, Iglesia I, Labayen I, Moreno LA, et al. Early life risk factors and their cumulative effects as predictors of overweight in Spanish children. International Journal of Public Health. 2018;63(4):501-12.
