# Supplementary material for: The association between maternal body mass index and child obesity: A systematic review and meta-analysis
Source: PLoS Med. 2019 Jun 11;16(6):e1002817. doi: 10.1371/journal.pmed.1002817 (PMC6559702; doi:10.1371/journal.pmed.1002817)
Supplement: S6 Table — (DOCX) [file pmed.1002817.s016.docx]

# S6 Table: Quality scores for all included studies

| **Paper** | **Newcastle Ottawa Scale question number and score allocated^a^** | | | | | | | |  | **Independent reviewer initials^d^** |
| --- | --- | --- | --- | --- | --- | --- | --- | --- | --- | --- |
|  | **1** | **2** | **3** | **4** | **5** | **6** | **7** | **Total stars^b^** | **Category^c^** |  |
| Ajslev *et al.* 2011[1] | a* | a* | c | a & b** | c | a* | b* | 6 | High | ZA & ES |
| Andres *et al.* 2015[2] | b* | a* | c | b* | a* | a* | d | 5 | Medium | ZA & ES |
| Basatemur *et al.* 2013[3] | a* | a* | b* | c | a* | a* | b* | 6 | High | HB & ZA |
| Berkowitz *et al.* 2005[4] | d | a* | d | b* | a* | a* | b* | 5 | Medium | HB & AP |
| Bider-Canfield *et al.* 2017[5] | a* | a* | a* | a & b** | a* | a* | b* | 8 | High | ZA & AP |
| Birbilis *et al.* 2013[6] | a* | a* | c | b* | a* | a* | a* | 6 | High | ZA & HB |
| Catalano *et al.* 2009[7] | a* | a* | a* | a & b** | a* | a* | d | 7 | High | ZA & LN |
| Daraki *et al.* 2015[8] | a* | a* | c | a & b** | a* | a* | c | 6 | High | HB & ZA |
| Davey Smith *et al.* 2007[9] | a* | a* | c | c | a* | a* | b* | 5 | Medium | ZA & AP |
| de Hoog *et al.* 2011[10] | b* | a* | c | c | a* | a* | b* | 5 | Medium | ZA & LN |
| Deierlein *et al.* 2011[11] | b* | a* | b* | b* | a* | a* | b* | 7 | High | HB & JR |
| Diesel *et al.* 2014[12] | c | a* | c | c | a* | a* | b* | 4 | Medium | HB & ZA |
| Durmus *et al.* 2012[13] | a* | a* | b* | b* | a* | a* | b* | 7 | High | HB & NH |
| Ehrenthal *et al.* 2013[14] | b* | a* | c | a & b** | b* | a* | b* | 7 | High | HB & RV |
| Eisenman *et al.* 2010[15] | a* | a* | c | c | d | a* | d | 3 | Medium | ZA & LN |
| Fleten *et al.* 2012[16] | a* | a* | b* | a & b** | c | a* | b* | 7 | High | ZA & AP |
| Gademan *et al.* 2014[17] | a* | a* | c | b* | a* | a* | b* | 6 | High | HB & JR |
| Gaillard *et al.* 2014[18] | a* | a* | a* | b* | a* | a* | b* | 7 | High | ZA & ES |
| Gillman *et al.* 2008[19] | a* | a* | b* | b* | a* | a* | b* | 7 | High | ZA & HB |
| Guo *et al.* 2015[20] | a* | a* | a* | b* | a* | a* | b* | 7 | High | ZA & HB |
| Hinkle *et al.* 2012[21] | c | a* | c | a & b** | a* | a* | b* | 6 | High | HB & ZA |
| Jacota *et al.* 2016[22] | a* | a* | c | c | a* | a* | b* | 5 | Medium | ZA & LN |
| Janjua *et al.* 2012[23] | c | a* | b* | b* | a* | a* | b* | 6 | High | HB & ZA & NH (Pilot) |
| Jharap *et al.* 2017[24] | a* | a* | c | a & b** | a* | a* | b* | 7 | High | ZA & LN |
| Jin *et al.* 2016[25] | b* | a* | a* | a & b** | a* | a* | a* | 8 | High | ZA & AP |
| Kaar *et al.* 2014[26] | a* | a* | a* | a & b** | a* | a* | d | 7 | High | ZA & ES |
| Kitsantas *et al.* 2010[27] | c | a* | c | b* | a* | a* | d | 4 | Medium | ZA & ES |
| Knight *et al.* 2007[28] | b* | a* | c | a* | a* | a* | d | 5 | Medium | ZA & HB |
| Kubo *et al.* 2016[29] | a* | a* | c | c | a* | a* | b* | 5 | Medium | ZA & LN & ES (Pilot) |
| Laitinen *et al.* 2012[30] | a* | a* | c | a & b** | a* | a* | b* | 7 | High | ZA & LN |
| Leng *et al.* 2015[31] | c | a* | a* | b* | a* | a* | a* | 6 | High | ZA & LN |
| Li *et al.* 2005[32] | a* | a* | c | b* | a* | a* | c | 5 | Medium | ZA & LN |
| Li *et al.* 2013[33] | a* | a* | a* | a & b** | a* | a* | b* | 8 | High | ZA & LN |
| Li *et al.* 2017[34] | b* | a* | c | a & b** | a* | a* | b* | 7 | High | ZA & AP |
| Lindberg *et al.* 2012[35] | c | a* | d | c | a* | a* | b* | 4 | Medium | ZA & ES |
| Makela *et al.* 2013[36] | c | a* | a* | c | a* | a* | b* | 5 | Medium | ZA & LN |
| Mamun *et al.* 2005[37] | a* | a* | b* | b* | a* | a* | b* | 7 | High | ZA & LN |
| Margerison Zilko *et al.* 2012[38] | b* | a* | a* | b* | a* | a* | b* | 7 | High | HB & AP |
| Massion *et al.* 2016[39] | b* | a* | d | c | a* | a* | d | 4 | Medium | ZA & HB |
| Mesman *et al.* 2009[40] | a* | a* | c | b* | a* | a* | b* | 6 | High | ZA & LN |
| Morgen *et al.* 2017[41] | a* | a* | c | b* | c | a* | b* | 5 | Medium | ZA & LN |
| O’Callaghan *et al.* 1997[42] | a* | a* | c | b* | a* | a* | c | 5 | Medium | HB & ZA |
| Oken *et al.* 2008[43] | b* | a* | c | b* | c | a* | b* | 5 | Medium | ZA & ES |
| Olson *et al.* 2009[44] | b* | a* | a* | b* | b* | a* | b* | 7 | High | HB & AP |
| Olson *et al.* 2010[45] | a* | a* | a* | b* | b* | a* | b* | 7 | High | HB & ZA |
| Pham *et al.* 2013[46] | b* | a* | a* | a & b** | a* | a* | d | 7 | High | ZA & HB |
| Rath *et al.* 2016[47] | a* | a* | c | c | a* | a* | b* | 5 | Medium | ZA & AP |
| Rathnayake *et al.* 2013[48] | d | a* | a* | b* | a* | a* | a* | 6 | High | ZA & LN |
| Reilly *et al.* 2005[49] | a* | a* | c | b* | a* | a* | b* | 6 | High | ZA & LN |
| Rios-Castillo *et al.* 2015[50] | a* | a* | c | c | a* | a* | b* | 5 | Medium | ZA & LN |
| Risvas *et al.* 2012[51] | a* | a* | c | b* | a* | a* | b* | 6 | High | ZA & LN |
| Robinson *et al.* 2014[52] | a* | a* | c | b* | b* | a* | b* | 6 | High | HB & NH |
| Rooney *et al.* 2011[53] | b* | a* | c | a & b** | b* | a* | b* | 7 | High | HB & AP |
| Salsberry *et al.* 2005[54] | a* | a* | c | b* | b* | a* | b* | 5 | Medium | HB & NH |
| Salsberry *et al.* 2007[55] | a* | a* | c | b* | a* | a* | b* | 6 | High | ZA & LN |
| Sorensen *et al.* 2016[56] | b* | a* | c | a & b** | c | a* | b* | 6 | High | ZA & AP |
| Stamnes Kopp *et al.* 2012[57] | a* | a* | b* | b* | c | a* | b* | 6 | High | HB & ZA |
| Tan *et al.* 2015[58] | a* | a* | c | b* | a* | a* | a* | 6 | High | ZA & LN |
| Terry *et al.* 2011[59] | a* | a* | c | a & b** | a* | a* | a* | 7 | High | HB & ZA |
| Toemen *et al.* 2016[60] | a* | a* | c | c | a* | a* | b* | 5 | Medium | ZA & LN |
| Wen *et al.* 2014[61] | b* | a* | c | b* | a* | a* | b* | 6 | High | ZA & HB |
| Weng *et al.* 2013[62] | d | a* | c | a & b** | d | a* | d | 4 | Medium | HB & JR |
| Whitaker 2004[63] | c | a* | a* | b* | b* | a* | c | 5 | Medium | HB & NH |
| Wojcicki *et al.* 2015[64] | b* | a* | c | b* | c | a* | c | 4 | Medium | HB & AP |
| Wrotniak *et al.* 2008[65] | a* | a* | c | a & b** | a* | a* | b* | 7 | High | ZA & HB |
| Zalbahar *et al.* 2015[66] | b* | a* | a* | a & b** | a* | a* | b* | 8 | High | ZA & ES |
| Zhang *et al.* 2013[67] | a* | a* | c | c | a* | a* | a* | 5 | Medium | ZA & LN |
| **Summary totals for 1^st^ search (out of 67)** | **n*=56**  **84%** | **n*=67**  **100%** | **n*=24**  **36%** | **n*=33**  **49%**  **n**=20**  **30%** | **n*=58**  **87%** | **n*=67**  **100%** | **n*=53**  **79%** | **Range 3 to 8** | **Low n=0**  **Medium n=25**  **37%**  **High n=42**  **63%** |  |
| Additional studies identified in the updated search March 2019 | | | | | | | | | | |
| Audrotsos *et al.* 2018[68] | a* | a* | c | b* | a* | a* | a* | 6 | High | LN & NH^e^ |
| Aris *et al.* 2018[69] | b* | a* | a* | b* | a* | a* | b* | 7 | High | LN & NH^e^ |
| Bridgman *et al.* 2018[70] | a* | a* | b* | a & b** | a* | a* | c | 7 | High | LN & NH^e^ |
| Fujita *et al.* 2018[71] | a* | a* | b* | b* | b* | a* | b* | 7 | High | LN & NH^e^ |
| Iguacel *et al.* 2018[72] | a* | a* | c | b* | b* | a* | a* | 6 | High | LN & NH^e^ |
| Kjaer *et al.* 2019[73] | b* | a* | c | a & b** | a* | a* | b* | 7 | High | HB & NH^e^ |
| Mao *et al.* 2017[74] | a* | a* | b* | a & b** | a* | a* | a* | 8 | High | HB & NH^e^ |
| Mintjens *et al.* 2018[75] | a* | a* | c | c | a* | a* | b* | 5 | Medium | LN & NH^e^ |
| Toftemo *et al.* 2018[76] | a* | a* | c | a & b** | a* | a* | b* | 7 | High | HB & NH^e^ |
| Wallby *et al.* 2017[77] | b* | a* | a* | b* | b* | a* | b* | 7 | High | HB & NH^e^ |
| Zhang *et al.* 2018[78] | a* | a* | a* | b* | a* | a* | a* | 7 | High | HB & NH^e^ |
| Zheng *et al.* 2019[79] | a* | a* | c | b* | a* | a* | b* | 6 | High | HB & NH^e^ |
| **Summary totals including updated search (out of 79)** | **n*=68**  **86%** | **n*=79**  **100%** | **n*=30**  **38%** | **n*= 40**  **49%**  **n**= 24**  **30%** | **n*=70**  **89%** | **n*=79**  **100%** | **n*=64**  **81%** | **Range 3 to 8** | **Low n=0**  **Medium n=26**  **33%**  **High n=53**  **67%** |  |

Footnote:

^b^Newcastle-Ottawa question numbers 1-7, answers a-d, and associated number of stars (*) are detailed in S2 Fig.

^b^Minimum number of possible stars to be awarded = 0, maximum number of possible stars to be awarded = 8.

^c^Categories were allocated as: Low = 0-2 stars, Medium = 3-5 stars, High = 6-8 stars.

^d^Reviewers initials relate to manuscript authors: NH, Nicola Heslehurst; RV, Rute Vieira; ZA, Zainab Akhter; HB, Hayley Bailey; ES, Emma Slack; LN, Lem Ngongalah; AP, Augustina Pemu; and JR, Judith Rankin.

^e^Quality assessment carried out by LN and HB and validated by NH for all additional studies included following the updated searches in March 2019

**References:**

1. Ajslev TA, Andersen CS, Gamborg M, Sorensen TI, Jess T. Childhood overweight after establishment of the gut microbiota: the role of delivery mode, pre-pregnancy weight and early administration of antibiotics. Int J Obes (Lond). 2011;35(4):522-9.

2. Andres A, Hull HR, Shankar K, Casey PH, Cleves MA, Badger TM. Longitudinal body composition of children born to mothers with normal weight, overweight, and obesity. Obesity (Silver Spring). 2015;23(6):1252-8.

3. Basatemur E, Gardiner J, Williams C, Melhuish E, Barnes J, Sutcliffe A. Maternal prepregnancy BMI and child cognition: a longitudinal cohort study. Pediatrics. 2013;131(1):56-63.

4. Berkowitz RI, Stallings VA, Maislin G, Stunkard AJ. Growth of children at high risk of obesity during the first 6 y of life: implications for prevention. Am J Clin Nutr. 2005;81(1):140-6.

5. Bider-Canfield Z, Martinez MP, Wang X, Yu W, Bautista MP, Brookey J, et al. Maternal obesity, gestational diabetes, breastfeeding and childhood overweight at age 2 years. Pediatric Obesity. 2017;12(2):171-8.

6. Birbilis M, Moschonis G, Mougios V, Manios Y, Healthy Growth Study g. Obesity in adolescence is associated with perinatal risk factors, parental BMI and sociodemographic characteristics. Eur J Clin Nutr. 2013;67(1):115-21.

7. Catalano PM, Farrell K, Thomas A, Huston-Presley L, Mencin P, de Mouzon SH, et al. Perinatal risk factors for childhood obesity and metabolic dysregulation. Am J Clin Nutr. 2009;90(5):1303-13.

8. Daraki V, Georgiou V, Papavasiliou S, Chalkiadaki G, Karahaliou M, Koinaki S, et al. Metabolic profile in early pregnancy is associated with offspring adiposity at 4 years of age: the Rhea pregnancy cohort Crete, Greece. PLoS ONE. 2015;10(5):e0126327.

9. Davey Smith G, Steer C, Leary S, Ness A. Is there an intrauterine influence on obesity? Evidence from parent child associations in the Avon Longitudinal Study of Parents and Children (ALSPAC). Archives of disease in childhood. 2007;92(10):876-80. Epub 2007/06/28.

10. de Hoog ML, van Eijsden M, Stronks K, Gemke RJ, Vrijkotte TG. Overweight at age two years in a multi-ethnic cohort (ABCD study): the role of prenatal factors, birth outcomes and postnatal factors. BMC Public Health. 2011;11(1):611.

11. Deierlein AL, Siega-Riz AM, Chantala K, Herring AH. The association between maternal glucose concentration and child BMI at age 3 years. Diabetes Care. 2011;34(2):480-4.

12. Diesel JC, Eckhardt CL, Day NL, Brooks MM, Arslanian SA, Bodnar LM. Is gestational weight gain associated with offspring obesity at 36 months? Pediatr Obes. 2014;10(4):305-10. Epub 2014/10/01.

13. Durmus B, Arends LR, Ay L, Hokken-Koelega AC, Raat H, Hofman A, et al. Parental anthropometrics, early growth and the risk of overweight in pre-school children: the Generation R Study. Pediatric Obesity. 2012;8(5):339-50.

14. Ehrenthal DB, Maiden K, Rao A, West DW, Gidding SS, Bartoshesky L, et al. Independent relation of maternal prenatal factors to early childhood obesity in the offspring. Obstet Gynecol. 2013;121(1):115-21.

15. Eisenman JC, Sarzynski MA, Tucker J, Heelan KA. Maternal prepregnancy overweight and offspring fatness and blood pressure: role of physical activity. Pediatr Exerc Sci. 2010;22(3):369-78.

16. Fleten C, Nystad W, Stigum H, Skjaerven R, Lawlor DA, Davey Smith G, et al. Parent-offspring body mass index associations in the Norwegian Mother and Child Cohort Study: a family-based approach to studying the role of the intrauterine environment in childhood adiposity. Am J Epidemiol. 2012;176(2):83-92.

17. Gademan MG, Vermeulen M, Oostvogels AJ, Roseboom TJ, Visscher TL, van Eijsden M, et al. Maternal prepregancy BMI and lipid profile during early pregnancy are independently associated with offspring's body composition at age 5-6 years: the ABCD study. PLoS ONE. 2014;9(4):e94594.

18. Gaillard R, Steegers EA, Duijts L, Felix JF, Hofman A, Franco OH, et al. Childhood cardiometabolic outcomes of maternal obesity during pregnancy: the Generation R Study. Hypertension. 2014;63(4):683-91.

19. Gillman MW, Rifas-Shiman SL, Kleinman K, Oken E, Rich-Edwards JW, Taveras EM. Developmental origins of childhood overweight: potential public health impact. Obesity (Silver Spring). 2008;16(7):1651-6.

20. Guo L, Liu J, Ye R, Liu J, Zhuang Z, Ren A. Gestational Weight Gain and Overweight in Children Aged 3-6 Years. J Epidemiol. 2015;25(8):536-43.

21. Hinkle SN, Sharma AJ, Swan DW, Schieve LA, Ramakrishnan U, Stein AD. Excess gestational weight gain is associated with child adiposity among mothers with normal and overweight prepregnancy weight status. J Nutr. 2012;142(10):1851-8.

22. Jacota M, Forhan A, Saldanha-Gomes C, Charles MA, Heude B, for the EMCCSG. Maternal weight prior and during pregnancy and offspring's BMI and adiposity at 5–6 years in the EDEN mother–child cohort. Pediatric Obesity. 2016.

23. Janjua NZ, Mahmood B, Islam MA, Goldenberg RL. Maternal and early childhood risk factors for overweight and obesity among low-income predominantly black children at age five years: A prospective cohort study. Journal of Obesity. 2012;457173.

24. Jharap VV, Santos S, Steegers EAP, Jaddoe VWV, Gaillard R. Associations of maternal obesity and excessive weight gain during pregnancy with subcutaneous fat mass in infancy. Early Human Development. 2017;108:23-8.

25. Jin WY, Lv Y, Bao Y, Tang L, Zhu ZW, Shao J, et al. Independent and combined effects of maternal prepregnancy body mass index and gestational weight gain on offspring growth at 0-3 years of age. BioMed Research International. 2016;4720785.

26. Kaar JL, Crume T, Brinton JT, Bischoff KJ, McDuffie R, Dabelea D. Maternal obesity, gestational weight gain, and offspring adiposity: the exploring perinatal outcomes among children study. J Pediatr. 2014;165(3):509-15.

27. Kitsantas P, Pawloski LR, Gaffney KF. Maternal prepregnancy body mass index in relation to Hispanic preschooler overweight/obesity. Eur J Pediatr. 2010;169(11):1361-8.

28. Knight B, Shields BM, Hill A, Powell RJ, Wright D, Hattersley AT. The impact of maternal glycemia and obesity on early postnatal growth in a nondiabetic Caucasian population. Diabetes Care. 2007;30(4):777-83.

29. Kubo A, Ferrara A, Laurent CA, Windham GC, Greenspan LC, Deardorff J, et al. Associations Between Maternal Pregravid Obesity and Gestational Diabetes and the Timing of Pubarche in Daughters. Am J Epidemiol. 2016;184(1):7-14.

30. Laitinen J, Jaaskelainen A, Hartikainen AL, Sovio U, Vaarasmaki M, Pouta A, et al. Maternal weight gain during the first half of pregnancy and offspring obesity at 16 years: a prospective cohort study. Bjog. 2012;119(6):716-23.

31. Leng J, Li W, Zhang S, Liu H, Wang L, Liu G, et al. GDM Women's Pre-Pregnancy Overweight/Obesity and Gestational Weight Gain on Offspring Overweight Status. PLoS ONE. 2015;10(6):e0129536.

32. Li C, Kaur H, Choi WS, Huang TT, Lee RE, Ahluwalia JS. Additive interactions of maternal prepregnancy BMI and breast-feeding on childhood overweight. Obes Res. 2005;13(2):362-71.

33. Li N, Liu E, Guo J, Pan L, Li B, Wang P, et al. Maternal prepregnancy body mass index and gestational weight gain on offspring overweight in early infancy. PLoS ONE. 2013;8(10):e77809.

34. Li A, Teo KK, Morrison KM, McDonald SD, Atkinson SA, Anand SS, et al. A genetic link between prepregnancy body mass index, postpartum weight retention, and offspring weight in early childhood. Obesity (Silver Spring). 2017;25(1):236-43.

35. Lindberg SM, Adams AK, Prince RJ. Early predictors of obesity and cardiovascular risk among American Indian children. Matern Child Health J. 2012;16(9):1879-86.

36. Makela J, Lagstrom H, Kaljonen A, Simell O, Niinikoski H. Hyperglycemia and lower diet quality in pregnant overweight women and increased infant size at birth and at 13 months of age--STEPS study. Early Human Development. 2013;89(6):439-44.

37. Mamun AA, Lawlor DA, O'Callaghan MJ, Williams GM, Najman JM. Family and early life factors associated with changes in overweight status between ages 5 and 14 years: findings from the Mater University Study of Pregnancy and its outcomes. Int J Obes. 2005;29(5):475-82.

38. Margerison-Zilko CE, Shrimali BP, Eskenazi B, Lahiff M, Lindquist AR, Abrams BF. Trimester of maternal gestational weight gain and offspring body weight at birth and age five. Matern Child Health J. 2012;16(6):1215-23.

39. Massion S, Wickham S, Pearce A, Barr B, Law C, Taylor-Robinson D. Exploring the impact of early life factors on inequalities in risk of overweight in UK children: findings from the UK Millennium Cohort Study. Archives of disease in childhood. 2016. Epub 2016/05/11.

40. Mesman I, Roseboom TJ, Bonsel GJ, Gemke RJ, van der Wal MF, Vrijkotte TGM. Maternal pre-pregnancy body mass index explains infant’s weight and BMI at 14 months: results from a multi-ethnic birth cohort study. Archives of disease in childhood. 2009;94(8):587-95.

41. Morgen C, Angquist L, Baker J, Andersen A, Michaelsen K, SoRensen T. Prenatal risk factors infuencing childhood BMI and overweight independent of birth weight and infancy BMI - A path analysis within the Danish national birth cohort. Obesity Facts. 2017;10:21-2.

42. O'Callaghan MJ, Williams GM, Andersen MJ, Bor W, Najman JM. Prediction of obesity in children at 5 years: a cohort study. J Paediatr Child Health. 1997;33(4):311-6.

43. Oken E, Rifas-Shiman SL, Field AE, Frazier AL, Gillman MW. Maternal gestational weight gain and offspring weight in adolescence. Obstet Gynecol. 2008;112(5):999-1006.

44. Olson CM, Strawderman MS, Dennison BA. Maternal weight gain during pregnancy and child weight at age 3 years. Matern Child Health J. 2009;13(6):839-46.

45. Olson CM, Demment MM, Carling SJ, Strawderman MS. Associations Between Mothers' and Their Children's Weights at 4 Years of Age. Childhood obesity (Print). 2010;6(4):201-7. Epub 2011/07/12.

46. Pham MT, Brubaker K, Pruett K, Caughey AB. Risk of childhood obesity in the toddler offspring of mothers with gestational diabetes. Obstet Gynecol. 2013;121(5):976-82.

47. Rath SR, Marsh JA, Newnham JP, Zhu K, Atkinson HC, Mountain J, et al. Parental pre-pregnancy BMI is a dominant early-life risk factor influencing BMI of offspring in adulthood. Obesity Science and Practice. 2016;2(1):48-57.

48. Rathnayake KM, Satchithananthan A, Mahamithawa S, Jayawardena R. Early life predictors of preschool overweight and obesity: a case-control study in Sri Lanka. BMC Public Health. 2013;13:994.

49. Reilly JJ, Armstrong J, Dorosty AR, Emmett PM, Ness A, Rogers I, et al. Early life risk factors for obesity in childhood: cohort study. BMJ. 2005;330(7504):1357.

50. Rios-Castillo I, Cerezo S, Corvalan C, Martinez M, Kain J. Risk factors during the prenatal period and the first year of life associated with overweight in 7-year-old low-income Chilean children. Maternal and Child Nutrition. 2015;11(4):595-605.

51. Risvas G, Papaioannou I, Panagiotakos DB, Farajian P, Bountziouka V, Zampelas A. Perinatal and family factors associated with preadolescence overweight/obesity in Greece: the GRECO study. J Epidemiol Glob Health. 2012;2(3):145-53.

52. Robinson CA, Cohen AK, Rehkopf DH, Deardorff J, Ritchie L, Jayaweera RT, et al. Pregnancy and post-delivery maternal weight changes and overweight in preschool children. Prev Med. 2014;60:77-82.

53. Rooney BL, Mathiason MA, Schauberger CW. Predictors of obesity in childhood, adolescence, and adulthood in a birth cohort. Matern Child Health J. 2011;15(8):1166-75.

54. Salsberry PJ, Reagan PB. Dynamics of early childhood overweight. Pediatrics. 2005;116(6):1329-38.

55. Salsberry PJ, Reagan PB. Taking the long view: the prenatal environment and early adolescent overweight. Res Nurs Health. 2007;30(3):297-307.

56. Sorensen TIA, Ajslev TA, Angquist L, Morgen CS, Ciuchi IG, Smith GD. Comparison of associations of maternal peri-pregnancy and paternal anthropometrics with child anthropometrics from birth through age 7 y assessed in the Danish National Birth Cohort. Am J Clin Nutr. 2016;104(2):389-96.

57. Stamnes Kopp UM, Dahl-Jorgensen K, Stigum H, Frost Andersen L, Naess O, Nystad W. The associations between maternal pre-pregnancy body mass index or gestational weight change during pregnancy and body mass index of the child at 3 years of age. Int J Obes (Lond). 2012;36(10):1325-31.

58. Tan HC, Roberts J, Catov J, Krishnamurthy R, Shypailo R, Bacha F. Mother's pre-pregnancy BMI is an important determinant of adverse cardiometabolic risk in childhood. Pediatric Diabetes. 2015;16(6):419-26.

59. Terry MB, Wei Y, Esserman D, McKeague IW, Susser E. Pre- and postnatal determinants of childhood body size: cohort and sibling analyses. Journal of Developmental Origins of Health and Disease. 2011;2(2):99-111. Epub 03/02.

60. Toemen L, Gishti O, Van Osch-Gevers L, Steegers EAP, Helbing WA, Felix JF, et al. Maternal obesity, gestational weight gain and childhood cardiac outcomes: Role of childhood body mass index. Int J Obes. 2016;40(7):1070-8.

61. Wen LM, Baur LA, Rissel C, Xu H, Simpson JM. Correlates of body mass index and overweight and obesity of children aged 2 years: findings from the healthy beginnings trial. Obesity (Silver Spring). 2014;22(7):1723-30.

62. Weng SF, Redsell SA, Nathan D, Swift JA, Yang M, Glazebrook C. Estimating overweight risk in childhood from predictors during infancy. Pediatrics. 2013;132(2):e414-21.

63. Whitaker RC. Predicting preschooler obesity at birth: the role of maternal obesity in early pregnancy. Pediatrics. 2004;114(1):e29-36.

64. Wojcicki JM, Young MB, Perham-Hester KA, de Schweinitz P, Gessner BD. Risk factors for obesity at age 3 in Alaskan children, including the role of beverage consumption: results from Alaska PRAMS 2005-2006 and its three-year follow-up survey, CUBS, 2008-2009. PLoS ONE. 2015;10(3):e0118711.

65. Wrotniak BH, Shults J, Butts S, Stettler N. Gestational weight gain and risk of overweight in the offspring at age 7 y in a multicenter, multiethnic cohort study. Am J Clin Nutr. 2008;87(6):1818-24.

66. Zalbahar N, Jan Mohamed HJB, Loy SL, Najman J, McIntyre HD, Mamun A. Association of parental body mass index before pregnancy on infant growth and body composition: Evidence from a pregnancy cohort study in Malaysia. Obesity Research and Clinical Practice. 2016;10:S35-S47.

67. Zhang J, Himes JH, Guo Y, Jiang J, Yang L, Lu Q, et al. Birth weight, growth and feeding pattern in early infancy predict overweight/obesity status at two years of age: a birth cohort study of Chinese infants. PLoS ONE. 2013;8(6):e64542.

68. Androutsos O, Moschonis G, Ierodiakonou D, Karatzi K, De Bourdeaudhuij I, Iotova V, et al. Perinatal and lifestyle factors mediate the association between maternal education and preschool children's weight status: the ToyBox study. Nutrition. 2018;48:6-12.

69. Aris IM, Bernard JY, Chen LW, Tint MT, Pang WW, Soh S-E, et al. Modifiable risk factors in the first 1000 days for subsequent risk of childhood overweight in an Asian cohort: significance of parental overweight status. Int J Obes. 2018;42(1):44.

70. Bridgman SL, Azad MB, Persaud RR, Chari RS, Becker AB, Sears MR, et al. Impact of maternal pre‐pregnancy overweight on infant overweight at 1 year of age: associations and sex‐specific differences. Pediatric Obesity. 2018;13(10):579-89.

71. Fujita Y, Kouda K, Nakamura H, Iki M. Relationship Between Maternal Pre-pregnancy Weight and Offspring Weight Strengthens as Children Develop: A Population-Based Retrospective Cohort Study. J Epidemiol. 2018:JE20170137.

72. Iguacel I, Escartín L, Fernández-Alvira JM, Iglesia I, Labayen I, Moreno LA, et al. Early life risk factors and their cumulative effects as predictors of overweight in Spanish children. International Journal of Public Health. 2018;63(4):501-12.

73. Kjaer TW, Faurholt-Jepsen D, Medrano R, Elwan D, Mehta K, Christensen VB, et al. Higher birthweight and maternal pre-pregnancy BMI persist with obesity association at age 9 in high risk Latino children. Journal of Immigrant and Minority Health. 2019:1-9.

74. Mao G, Nachman RM, Sun Q, Zhang X, Koehler K, Chen Z, et al. Individual and Joint Effects of Early-Life Ambient PM 2.5 Exposure and Maternal Prepregnancy Obesity on Childhood Overweight or Obesity. Environmental Health Perspectives. 2017;125(6):067005.

75. Mintjens S, Gemke RJBJ, van Poppel MNM, Vrijkotte TGM, Roseboom TJ, van Deutekom AW. Maternal Prepregnancy Overweight and Obesity Are Associated with Reduced Physical Fitness But Do Not Affect Physical Activity in Childhood: The Amsterdam Born Children and Their Development Study. Childhood Obesity. 2018;15(1):31-9.

76. Toftemo I, Jenum AK, Lagerløv P, Júlίusson PB, Falk RS, Sletner L. Contrasting patterns of overweight and thinness among preschool children of different ethnic groups in Norway, and relations with maternal and early life factors. BMC Public Health. 2018;18(1):1056.

77. Wallby T, Lagerberg D, Magnusson M. Relationship between breastfeeding and early childhood obesity: Results of a prospective longitudinal study from birth to 4 years. Breastfeeding Medicine. 2017;12(1):48-53.

78. Zhang W, Niu F, Ren X. Association of maternal pre‐pregnancy body mass index and gestational weight gain with Chinese infant growth. Journal of Paediatrics and Child Health. 2018.

79. Zheng M, Bowe SJ, Hesketh KD, Bolton K, Laws R, Kremer P, et al. Relative effects of postnatal rapid growth and maternal factors on early childhood growth trajectories. Paediatric and Perinatal Epidemiology. 2019.
