# Supplementary material for: The association between maternal body mass index and child obesity: A systematic review and meta-analysis
Source: PLoS Med. 2019 Jun 11;16(6):e1002817. doi: 10.1371/journal.pmed.1002817 (PMC6559702; doi:10.1371/journal.pmed.1002817)
Supplement: S7 Table — (DOCX) [file pmed.1002817.s017.docx]

# S7 Table: Contacting authors for additional information

| **Paper** | **Essential data requested^a^** | **Non-essential data requested^b^** | **Response** | **Data provided** |
| --- | --- | --- | --- | --- |
| Ajslev *et al.* 2011[1] | - Maternal BMI frequency data | - The definitions of maternal recommended weight for underweight and normal - Frequency data for all child normal, overweight and obese categories. - Confirm if the AOR 1.113 (1.12-1.14) in the paper is for combined child overweight/obese and combined maternal overweight/obese | No response^c^ | None |
| Andres *et al.* 2015[2] | - Mean child BMI z-score and SD for each child age (1, 2, 3, 4, 5, and 6 years) - Maternal BMI category and frequency data for each maternal BMI category if possible | None | Data provided | **Raw data combined for both sexes**  **Age 1**  RW: mean=0.56, SD=0.90, n=18  OW: mean=0.61, SD=0.90, n=28  OB: mean=1.04, SD=0.94, n=26  **Age 2**  RW: mean=0.43, SD=0.90, n=28  OW: mean=0.82, SD=0.90, n=28  OB: mean=0.71, SD=0.78, n=27  **Age 3**  RW: mean=0.27, SD=0.96, n=18  OW: mean=0.19, SD=0.62, n=18  OB: mean=0.50, SD=0.73, n=35  **Age 4**  RW: mean=0.10, SD=0.80, n=65  OW: mean=0.24, SD=0.68, n=37  OB: mean=0.87, SD=1.08, n=46  **Age 5**  RW: mean=0.13, SD=0.87, n=76  OW: mean=0.52, SD=1.03, n=46  OB: mean=1.03, SD=1.23, n=51  **Age 6**  RW: mean=0.04, SD=0.97, n=69  OW: mean=0.60, SD=1.12, n=31  OB: mean=0.81, SD=1.17, n=47  **Data adjusted for covariates**  **Age 1**  RW: mean=0.52, SD=0.15  OW: mean=0.80, SD=0.13  OB: mean=0.86, SD=0.14  **Age 2**  RW: mean=0.55, SD=0.12  OW: mean=0.73, SD=0.12  OB: mean=0.70, SD=0.12  **Age 3**  RW: mean=0.30, SD=0.10  OW: mean=0.44, SD=0.13  OB: mean=0.58, SD=0.11  **Age 4**  RW: mean=0.16, SD=0.10  OW: mean=0.37, SD=0.12  OB: mean=0.81, SD=0.11  **Age 5**  RW: mean=0.19, SD=0.11  OW: mean=0.51, SD=0.13  OB: mean=0.90, SD=0.12  **Age 6**  RW: mean=0.09, SD=0.12  OW: mean=0.52, SD=0.15  OB: mean=0.80, SD=0.18  Note: the authors also provided data separated by child sex |
| Basatemur *et al.* 2013[3] | - SD of child BMI for each maternal BMI category, for child ages 5 and 7 | - Frequency data for child BMI z-scores normal weight, overweight and obese, for each maternal BMI category | No response^c^ | None |
| Berkowitz *et al.* 2005[4] | None | - Frequency of child overweight and obese for the maternal high and low risk categories; for child age 4 Grouped maternal BMI categories according to WHO BMI criteria Confirm of whether the control group for child overweight/obese analysis was <85^th^ percentile | No response^c^ | None |
| Bider-Canfield *et al.* 2017[5] | - Number or percentage of children who are normal weight and overweight | - Number or percentage of children in each maternal BMI category | Unable to provide^d^ | None |
| Birbilis *et al.* 2013[6] | - Frequency data for maternal and child BMI categories - Definition of the child weight comparison group used in the analysis | None | No response^c^ | None |
| Catalano *et al.* 2009[7] | - Frequency data for child normal weight, overweight and obese for each maternal BMI category | - Split maternal BMI category <30kg/m^2^ into <18.5kg/m^2^, 18.5-24.9kg/m^2^ and 25-29.9kg/m^2^ and provide child BMI frequency data if possible | Data provided | **Essential data**  **Maternal BMI<25kg/m^2^** (Number of children who were RW, OW and OB)  RW - 20  OW - 7  OB – 12  **Maternal BMI≥25kg/m^2^** (Number of children who were RW, OW and OB)  RW – 38  OW – 7  OB – 5  **Non-essential data**  **Maternal BMI<18.5kg/m^2^** (Number of children who were RW, OW and OB)  RW – 4  OW – 0  OB – 1  **Maternal BMI 18.5-24.9kg/m^2^** (Number of children who were RW, OW and OB)  RW – 34  OW – 7  OB – 4  **Maternal BMI 25-29.9kg/m^2^** (Number of children who were RW, OW and OB)  RW – 9  OW – 5  OB – 2  **Maternal BMI≥30kg/m^2^** (Number of children who were RW, OW and OB)  RW – 11  OW – 2  OB – 10 |
| Daraki *et al.* 2015[8] | None | - Frequency data for child weight separately for child overweight/obese and maternal overweight/obese categories | No response^c^ | None |
| de Hoog *et al.* 2011[9] | None | - Frequency data for child overweight/obese by maternal BMI category | Unable to provide^e^ | None |
| Diesel *et al.* 2014[10] | None | - Split child non-obese (<95^th^ percentile) group into separate categories of normal and overweight (<85th and 85-95th percentile) - Number of cases (or %) for each by maternal BMI category | No response^c^ | None |
| Deierlein *et al.* 2011[11] | None | - Frequency data for child overweight/obese for each maternal BMI category | No response^c^ | None |
| Durmus *et al.* 2012[12] | - Frequency data for child normal and overweight/obese groups for each maternal BMI category, for ages 1, 2 and 3 years old | - Split the maternal BMI category recommended weight/underweight into separate categories of recommended weight and underweight - Split the child overweight/obese category into separate categories of overweight and obese for ages 1-4 | No response^c^ | None |
| Ehrenthal *et al.* 2013[13] | - Number of women in each maternal BMI category - Mean child BMI and SD for each maternal BMI category | - Frequency data for each maternal BMI category | No response^c^ | None |
| Eisenman *et al.* 2010[14] | - Child mean, SD and n for each maternal BMI category | - Split maternal BMI category <25kg/m^2^ into <18.5kg/m^2^ and 18.5-24.9kg/m^2^ | No response^c^ | None |
| Gademan *et al.* 2014[15] | None | - Split the child overweight/obese group into separate categories of overweight and obese - Frequency data for each maternal BMI category | No response^c^ | None |
| Gaillard *et al.* 2014[16] | None | - Split the child overweight/obese group into separate categories of overweight and obese - Frequency data for each maternal BMI category | Data provided | **Maternal BMI<20kg/m^2^** (Number of children who were OW and OB)  OW – 45  OB – 12  **Maternal BMI 20-24.9kg/m^2^** (Number of children who were OW and OB)  OW – 318  OB – 75  **Maternal BMI 25-29.9kg/m^2^** (Number of children who were OW and OB)  OW – 185  OB - 60  **Maternal BMI≥30kg/m^2^** (Number of children who were OW and OB)  OW – 98  OB - 73 |
| Guo *et al.* 2015[17] | None | - Split maternal and child overweight/obese groups into separate categories of overweight and obese - Frequency data for each maternal BMI category | No response^c^ | None |
| Jacota *et al.* 2017[18] | None | - Linear regression co-efficient and SE for offspring BMI at 5 years | No response^c^ | None |
| Janjua *et al.* 2012[19] | None | - Frequency data for child overweight and recommended weight by maternal BMI category - Data for maternal underweight and recommended weight separately | No response^c^ | None |
| Jin *et al.* 2016[20] | None | - Mean BMI z-scores and SDs for ages 1, 2 and 3, for each maternal BMI category - Number of children at each age group - Number of children who are normal weight and overweight for each maternal BMI category, for child ages 1,2 and 3 | No response^c^ | None |
| Kaar *et al.* 2014[21] | - Mean child BMI, SD and number of children in each maternal BMI category | - Split maternal BMI≥25kg/m^2^ into 25-29.9kg/m^2^ and ≥30kg/m^2^ and provide frequency data for each - Frequency data by child age 6, 7, 8, 9, 10, 11, 12, 13 (currently reported combined 6-13 years) | Data provided | **Essential data**  RW: Mean=17.923, SD=3.466, n=227  OW/OB: Mean=20.411, SD=5.191, n=202  **Non-essential data**  OW: Mean=19.932, SD=4.862, n=111  OB: Mean=20.995, SD=5.537, n=91  **Age 6**  RW – Mean=14.230, SD=1.620, n=3  OW – Mean=13.553, SD=0.528, n=2  OB – no children in this group  **Age 7**  RW – Mean=15.330, SD=1.981, n=7  OW – Mean=16.665, SD=2.200, n=3  OB – Mean=15.395, SD=1.620, n=4  **Age 8**  RW – Mean=15.735, SD=2.695, n=27  OW – Mean=17.400, SD=3.345, n=14  OB – Mean=19.258, SD=4.345, n=16  **Age 9**  RW – Mean=17.686, SD=3.475, n=47  OW – Mean=18.068, SD=3.774, n=23  OB – Mean=18.946, SD=3.477, n=19  **Age 10**  RW – Mllean=17.142, SD=3.002, n=39  OW – Mean=19.913, SD=4.520, n=26  OB – Mean=22.684, SD=5.840, n=21  **Age 11**  RW – Mean=18.617, SD=3.483, n=69  OW – Mean=20.785, SD= 4.223, n=24  OB – Mean=21.190, SD=5.466, n=18  **Age 12**  RW – Mean=19.918, SD=3.343, n=34  OW – Mean=24.767, SD=6.131, n=12  OB – Mean=24.010, SD=6.634, n=11  **Age 13**  RW – Mean=21.560, n=1  OW – Mean=23.195, SD=4.852, n=7  OB – Mean=29.498, SD=7.888, n=2 |
| Kitsantas *et al.* 2010[22] | - Provide frequency data for maternal BMI categories for White/Hispanic mothers at 2 years and 4 years - Clarify if the numbers reported are the number of mothers in each BMI category at each time point or the number of children, cases or controls | - Split maternal overweight/obese categories and provide child frequency data for each - Split the child overweight group into overweight and obese categories | No response^c^ | None |
| Stamnes Kopp *et al.* 2012[23] | None | - Provide the SD of child BMI for each maternal BMI category - Provide frequency data for child overweight/obese for each maternal BMI category | No response^c^ | None |
| Kubo *et al.* 2016[24] | None | - Split the maternal BMI category <25kg/m^2^ into 18.5-25kg/m^2^ and <18.5kg/m^2^; and provide frequency data for each | Data provided | **Maternal UW** (number (%) of children who were RW, OW and OB)  RW – 9 (100)  OW – 0 (0.00)  OB – 0 (0.00)  N=9  **Maternal RW** (number (%) of children who were RW, OW and OB)  RW – 162 (79.02)  OW – 27 (13.70)  OB – 16 (7.80)  N=205  **Additional data provided**  **Maternal OW** (number (%) of children who were RW, OW and OB)  RW – 68 (65.38)  OW – 18 (17.31)  OB – 18 (17.31)  N=104  **Maternal OB** (number (%) of children who were RW, OW and OB)  RW – 40 (58.82)  OW – 10 (14.71)  OB – 18 (26.47)  N=68  Total number of children = 386  Total number of RW children = 279  Total number of OW children = 55  Total number of OB children = 52  Frequency missing = 35 |
| Laitinen *et al.* 2012[25] | None | - Split the child overweight/obese category and provide the number of cases for each | No response^c^ | None |
| Li *et al.* 2005[26] | - Provide frequency data for child overweight/obese for each maternal BMI category | - Provide frequency data for each child age 7, 8, 9 and 10 years - currently reported as 7-10 years | No response^c^ | None |
| Li *et al.* 2016[27] | None | - Provide β and SE for child BMI z-score analysis, for each child age | No response^c^ | None |
| Lindberg *et al.* 2012[28] | - Provide frequency data for child overweight/obese for each maternal BMI category (<25kg/m^2^, 25-29.9kg/m^2^ and ≥30kg/m^2^) - Define the reference group for ORs presented in the paper - Confirm if the reference group for the maternal BMI≥30kg/m^2^ analysis is BMI<30kg/m^2^ | None | No response^c^ | None |
| Makela *et al.* 2013[29] | None | - Provide categorical child BMI data (presented as continuous) | No response^c^ | None |
| Mamun *et al.* 2005[30] | None | - Split frequency data by maternal overweight/obese BMI categories and child overweight/obese categories, for both 5 years and 14 years data | No response^c^ | None |
| Margerison Zilko *et al.* 2012[31] | None | - Split maternal BMI overweight/obese into separate categories of overweight and obese and provide frequency data for each | Unable to provide^d^ | None |
| Massion *et al.* 2016[32] | None | - Split child overweight/obese and maternal overweight/obese groups and provide frequency data separately for each category | Data provided | **Maternal RW** (Number of children who were RW, OW and OB)  RW – 6554  OW – 534  OB – 259  **Maternal OW** (Number of children who were RW, OW and OB)  RW - 1691  OW - 278  OB – 175  **Maternal OB** (Number of children who were RW, OW and OB)  RW - 578  OW – 194  OB – 160  Total sample = 10423 |
| Mesman *et al.* 2009[33] | None | - Provide categorical child BMI data (presented as continuous) | No response^c^ | None |
| Olson *et al.* 2009[34] | - Provide the CI for the OR 1.109 (association between child and maternal BMI) | - Split the frequency data for overweight/obese into separate categories of overweight and obese for both maternal and child weight categories | Author responded | Not provided: study sample is a subset of the data provided below (Olson *et al.* 2010), so may not be appropriate to include both in meta-analysis |
| Olson *et al.* 2010[35] | - Provide the CI for OR 1.208 (85^th^-95^th^ vs <85^th^ percentile) and OR 2.268 (≥95^th^ vs <85^th^ percentile) | None | Data provided | - OR 1.208 (85^th^ -95^th^ percentile vs <85^th^ percentile) 95% CI = 0.0817, 1.786 - OR 2.268 (≥95^th^ percentile vs <85^th^ percentile) 95% CI = 1.414, 3.637 |
| Pham *et al.* 2013[36] | - Provide frequency data for child BMI≥85^th^ percentile, for each maternal BMI category | - Split child weight categories into 85-95th and ≥95th percentiles | Unable to provide^e^ | None |
| Rath *et al.* 2016[37] |  | - Provide the number of women in each maternal BMI category, for each child age - For children aged 1, provide the number of children who were normal weight and obese for each maternal BMI category | No response^c^ | None |
| Rathnayake *et al.* 2013[38] | - Provide frequency data for child cases and controls for each maternal weight category | - Split maternal overweight/recommended weight into separate categories and provide frequency of child overweight for each category | No response^c^ | None |
| Reilly *et al.* 2005[39] | - Provide frequency data for child cases and controls for each maternal weight category | None | No response^c^ | None |
| Rios-Castillo *et al.* 2015[40] | None | - Split the child overweight/obese and maternal overweight/obese groups into separate categories of overweight and obese; and provide frequency data of child cases and controls for each maternal BMI category | Unable to provide^d^ | None |
| Risvas *et al.* 2012[41] | - Provide frequency data for child overweight/obese for each maternal BMI category - Confirm if the reference group for maternal BMI is BMI<25kg/m^2^ | - Split the child overweight/obese group into separate categories of overweight and obese and provide frequency data for each maternal BMI category | No response^c^ | None |
| Robinson *et al.* 2014[42] | None | - Provide frequency data for maternal and child categories - Split the child overweight/obese category (currently ≥85^th^ percentile) into separate categories of 85^th^-95^th^ and ≥95th percentiles | No response^c^ | None |
| Rooney *et al.* 2011[43] | - Provide frequency data for child BMI≥85^th^ percentile for maternal BMI categories <30kg/m^2^ and ≥30kg/m^2^ | - Split maternal BMI category <30kg/m^2^ into WHO categories of underweight, normal weight and overweight; and child BMI≥85^th^ percentile into 85^th^-95^th^ and ≥95^th^ percentile | No response^c^ | None |
| Salsberry *et al.* 2007[44] | - Provide frequency data for child BMI ≥95^th^ percentile for each maternal BMI category | - Split child BMI categories into normal weight, overweight and obese; and maternal BMI<25kg/m^2^ into underweight and recommended weight | No response^c^ | None |
| Tan *et al.* 2015[45] | - Provide the 95% CI for the reported maternal and child BMI regression coefficients (0.19 adjusted, 0.4 unadjusted) | - Provide child frequency data for overweight and obese separately for each maternal BMI category | No response^c^ | None |
| Wen *et al.* 2014[46] | - Provide the mean child BMI, SD and frequency of child normal weight, overweight and obese for each maternal BMI category | None | No response^c^ | None |
| Weng *et al.* 2013[47] | None | - Split child overweight/obese into separate categories of overweight and obese and provide the number of cases or percentages for each maternal BMI category | Data provided | **Maternal UW** (Total number of children and number of those who were OW and OB)  N = 291  OW – 35  OB – 5  **Maternal RW** (Total number of children and number of those who were OW and OB)  N = 4583  OW – 958  OB – 187  **Maternal OW** (Total number of children and number of those who were OW and OB)  N =1444  OW – 417  OB – 98  **Maternal OB** (Total number of children and number of those who were OW and OB)  N = 695  OW – 233  OB – 65  **Author’s comment**  Note: This was the cohort we used to derive the algorithm from (it’s 75% of the total size) – validation cohort has very similar proportions. The overweight category includes overweight as well as obesity, so the n for those who were overweight but not obese will be overweight – obese |
| Whitaker 2004[48] | - Provide the number of women in each maternal BMI category, for each child age (2, 3, and 4 years) - Confirm if the analysis comparison group for child ≥95th percentile is child weight <95th percentile | - Provide the number of children in the child weight categories 85^th^-95^th^ and ≥95^th^ percentile, for each child age | No response^c^ | None |
| Wojcicki *et al.* 2015[49] | None | - Split the child overweight/obese (≥85th percentile) group into separate categories of overweight and obese, and provide the frequency data for each maternal BMI category | No response^c^ | None |
| Wrotniak *et al.* 2008[50] | None | - Split the child weight groups <95^th^ and ≥95^th^ percentile into <85^th^, 85^th^-95^th^ and ≥95^th^ percentile; and provide frequency data for each maternal BMI category | No response^c^ | None |
| Zhang *et al.* 2013[51] | None | - Confirm the control group definitions for the analysis of child BMI ≥85^th^ and ≥95^th^ percentile | No response^c^ | None |
| Fleten *et al.* 2012[52] | None | - Provide the actual figures for mean child BMI and SD for each maternal BMI category (shown on graph in report) - Provide the number of women in each maternal BMI category. - Provide frequency data for child BMI, for each maternal BMI category - Provide the regression co-efficient and confidence intervals for maternal and child BMI, for ages 1 and 2, as well as the number of children in the sample at age 1 and 2 | No response^c^ | None |
| Morgen *et al.* 2017[53] | None | - Provide the number of non-obese and obese children at ages 7 and 11; for maternal BMI categories <18.5kg/m^2^, 18.5-24.9kg/m^2^, 25-29.9kg/m^2^ and ≥30kg/m^2^ | Unable to provide^d^ | None |
| Leng *et al.* 2015[54] | None | - Provide the number or percentage of non-overweight (<85^th^ percentile) and overweight (≥85^th^ percentile) children in each maternal BMI category | No response^c^ | None |

Abbreviations: UW, underweight; RW, recommended weight; OW, overweight; OB, obese; N, total number in group.

Footnote:

^a^Data required for the study to be included in any meta-analysis.

^b^Additional data to enable the study to be included in multiple meta-analyses (e.g. linear and non-linear) or to facilitate more accurate estimates in the meta-analysis (e.g. using comparable reference groups).

^c^Non-responding authors contacted up to three times.

^d^Authors no longer have access to requested data.

^e^Referred to alternative author but no response received

**References:**

1. Ajslev TA, Andersen CS, Gamborg M, Sorensen TI, Jess T. Childhood overweight after establishment of the gut microbiota: the role of delivery mode, pre-pregnancy weight and early administration of antibiotics. Int J Obes. 2011;35(4):522-9.

2. Andres A, Hull HR, Shankar K, Casey PH, Cleves MA, Badger TM. Longitudinal body composition of children born to mothers with normal weight, overweight, and obesity. Obesity. 2015;23(6):1252-8.

3. Basatemur E, Gardiner J, Williams C, Melhuish E, Barnes J, Sutcliffe A. Maternal prepregnancy BMI and child cognition: a longitudinal cohort study. Pediatrics. 2013;131(1):56-63.

4. Berkowitz RI, Stallings VA, Maislin G, Stunkard AJ. Growth of children at high risk of obesity during the first 6 y of life: implications for prevention. Am J Clin Nutr. 2005;81(1):140-6.

5. Bider-Canfield Z, Martinez MP, Wang X, Yu W, Bautista MP, Brookey J, et al. Maternal obesity, gestational diabetes, breastfeeding and childhood overweight at age 2 years. Pediatric Obesity. 2017;12(2):171-8.

6. Birbilis M, Moschonis G, Mougios V, Manios Y, Healthy Growth Study Obesity in adolescence is associated with perinatal risk factors, parental BMI and sociodemographic characteristics. Eur J Clin Nutr. 2013;67(1):115-21.

7. Catalano PM, Farrell K, Thomas A, Huston-Presley L, Mencin P, de Mouzon SH, et al. Perinatal risk factors for childhood obesity and metabolic dysregulation. Am J Clin Nutr. 2009;90(5):1303-13.

8. Daraki V, Georgiou V, Papavasiliou S, Chalkiadaki G, Karahaliou M, Koinaki S, et al. Metabolic profile in early pregnancy is associated with offspring adiposity at 4 years of age: the Rhea pregnancy cohort Crete, Greece. PLoS ONE. 2015;10(5):e0126327.

9. de Hoog ML, van Eijsden M, Stronks K, Gemke RJ, Vrijkotte TG. Overweight at age two years in a multi-ethnic cohort (ABCD study): the role of prenatal factors, birth outcomes and postnatal factors. BMC Public Health. 2011;11(1):611.

10. Diesel JC, Eckhardt CL, Day NL, Brooks MM, Arslanian SA, Bodnar LM. Is gestational weight gain associated with offspring obesity at 36 months? Pediatr Obes. 2014;10(4):305-10. Epub 2014/10/01.

11. Deierlein AL, Siega-Riz AM, Chantala K, Herring AH. The association between maternal glucose concentration and child BMI at age 3 years. Diabetes Care. 2011;34(2):480-4.

12. Durmus B, Arends LR, Ay L, Hokken-Koelega AC, Raat H, Hofman A, et al. Parental anthropometrics, early growth and the risk of overweight in pre-school children: the Generation R Study. Pediatric Obesity. 2012;8(5):339-50.

13. Ehrenthal DB, Maiden K, Rao A, West DW, Gidding SS, Bartoshesky L, et al. Independent relation of maternal prenatal factors to early childhood obesity in the offspring. Obstet Gynecol. 2013;121(1):115-21.

14. Eisenman JC, Sarzynski MA, Tucker J, Heelan KA. Maternal prepregnancy overweight and offspring fatness and blood pressure: role of physical activity. Pediatr Exerc Sci. 2010;22(3):369-78.

15. Gademan MG, Vermeulen M, Oostvogels AJ, Roseboom TJ, Visscher TL, van Eijsden M, et al. Maternal prepregancy BMI and lipid profile during early pregnancy are independently associated with offspring's body composition at age 5-6 years: the ABCD study. PLoS ONE. 2014;9(4):e94594.

16. Gaillard R, Steegers EA, Duijts L, Felix JF, Hofman A, Franco OH, et al. Childhood cardiometabolic outcomes of maternal obesity during pregnancy: the Generation R Study. Hypertension. 2014;63(4):683-91.

17. Guo L, Liu J, Ye R, Liu J, Zhuang Z, Ren A. Gestational Weight Gain and Overweight in Children Aged 3-6 Years. J Epidemiol. 2015;25(8):536-43.

18. Jacota M, Forhan A, Saldanha-Gomes C, Charles MA, Heude B, for the EMCCSG. Maternal weight prior and during pregnancy and offspring's BMI and adiposity at 5–6 years in the EDEN mother–child cohort. Pediatric Obesity. 2016.

19. Janjua NZ, Mahmood B, Islam MA, Goldenberg RL. Maternal and early childhood risk factors for overweight and obesity among low-income predominantly black children at age five years: A prospective cohort study. Journal of Obesity. 2012.

20. Jin WY, Lv Y, Bao Y, Tang L, Zhu ZW, Shao J, et al. Independent and combined effects of maternal prepregnancy body mass index and gestational weight gain on offspring growth at 0-3 years of age. BioMed Research International. 2016.

21. Kaar JL, Crume T, Brinton JT, Bischoff KJ, McDuffie R, Dabelea D. Maternal obesity, gestational weight gain, and offspring adiposity: the exploring perinatal outcomes among children study. J Pediatr. 2014;165(3):509-15.

22. Kitsantas P, Pawloski LR, Gaffney KF. Maternal prepregnancy body mass index in relation to Hispanic preschooler overweight/obesity. Eur J Pediatr. 2010;169(11):1361-8.

23. Stamnes Kopp UM, Dahl-Jorgensen K, Stigum H, Frost Andersen L, Naess O, Nystad W. The associations between maternal pre-pregnancy body mass index or gestational weight change during pregnancy and body mass index of the child at 3 years of age. Int J Obes. 2012;36(10):1325-31.

24. Kubo A, Ferrara A, Laurent CA, Windham GC, Greenspan LC, Deardorff J, et al. Associations Between Maternal Pregravid Obesity and Gestational Diabetes and the Timing of Pubarche in Daughters. Am J Epidemiol. 2016;184(1):7-14.

25. Laitinen J, Jaaskelainen A, Hartikainen AL, Sovio U, Vaarasmaki M, Pouta A, et al. Maternal weight gain during the first half of pregnancy and offspring obesity at 16 years: a prospective cohort study. BJOG. 2012;119(6):716-23..

26. Li C, Kaur H, Choi WS, Huang TT, Lee RE, Ahluwalia JS. Additive interactions of maternal prepregnancy BMI and breast-feeding on childhood overweight. Obes Res. 2005;13(2):362-71.

27. Li A, Teo KK, Morrison KM, McDonald SD, Atkinson SA, Anand SS, et al. A genetic link between prepregnancy body mass index, postpartum weight retention, and offspring weight in early childhood. Obesity. 2017;25(1):236-43.

28. Lindberg SM, Adams AK, Prince RJ. Early predictors of obesity and cardiovascular risk among American Indian children. Matern Child Health J. 2012;16(9):1879-86.

29. Makela J, Lagstrom H, Kaljonen A, Simell O, Niinikoski H. Hyperglycemia and lower diet quality in pregnant overweight women and increased infant size at birth and at 13 months of age--STEPS study. Early Human Development. 2013;89(6):439-44.

30. Mamun AA, Lawlor DA, O'Callaghan MJ, Williams GM, Najman JM. Family and early life factors associated with changes in overweight status between ages 5 and 14 years: findings from the Mater University Study of Pregnancy and its outcomes. Int J Obes. 2005;29(5):475-82.

31. Margerison-Zilko CE, Shrimali BP, Eskenazi B, Lahiff M, Lindquist AR, Abrams BF. Trimester of maternal gestational weight gain and offspring body weight at birth and age five. Matern Child Health J. 2012;16(6):1215-23.

32. Massion S, Wickham S, Pearce A, Barr B, Law C, Taylor-Robinson D. Exploring the impact of early life factors on inequalities in risk of overweight in UK children: findings from the UK Millennium Cohort Study. Archives of disease in childhood. 2016. Epub 2016/05/11.

33. Mesman I, Roseboom TJ, Bonsel GJ, Gemke RJ, van der Wal MF, Vrijkotte TGM. Maternal pre-pregnancy body mass index explains infant’s weight and BMI at 14 months: results from a multi-ethnic birth cohort study. Archives of disease in childhood. 2009;94(8):587-95.

34. Olson CM, Strawderman MS, Dennison BA. Maternal weight gain during pregnancy and child weight at age 3 years. Matern Child Health J. 2009;13(6):839-46.

35. Olson CM, Demment MM, Carling SJ, Strawderman MS. Associations Between Mothers' and Their Children's Weights at 4 Years of Age. Childhood obesity. 2010;6(4):201-7.

36. Pham MT, Brubaker K, Pruett K, Caughey AB. Risk of childhood obesity in the toddler offspring of mothers with gestational diabetes. Obstet Gynecol. 2013;121(5):976-82.

37. Rath SR, Marsh JA, Newnham JP, Zhu K, Atkinson HC, Mountain J, et al. Parental pre-pregnancy BMI is a dominant early-life risk factor influencing BMI of offspring in adulthood. Obesity Science and Practice. 2016;2(1):48-57.

38. Rathnayake KM, Satchithananthan A, Mahamithawa S, Jayawardena R. Early life predictors of preschool overweight and obesity: a case-control study in Sri Lanka. BMC Public Health. 2013;13:994.

39. Reilly JJ, Armstrong J, Dorosty AR, Emmett PM, Ness A, Rogers I, et al. Early life risk factors for obesity in childhood: cohort study. BMJ. 2005;330(7504):1357.

40. Rios-Castillo I, Cerezo S, Corvalan C, Martinez M, Kain J. Risk factors during the prenatal period and the first year of life associated with overweight in 7-year-old low-income Chilean children. Maternal and Child Nutrition. 2015;11(4):595-605.

41. Risvas G, Papaioannou I, Panagiotakos DB, Farajian P, Bountziouka V, Zampelas A. Perinatal and family factors associated with preadolescence overweight/obesity in Greece: the GRECO study. J Epidemiol Glob Health. 2012;2(3):145-53.

42. Robinson CA, Cohen AK, Rehkopf DH, Deardorff J, Ritchie L, Jayaweera RT, et al. Pregnancy and post-delivery maternal weight changes and overweight in preschool children. Prev Med. 2014;60:77-82.

43. Rooney BL, Mathiason MA, Schauberger CW. Predictors of obesity in childhood, adolescence, and adulthood in a birth cohort. Matern Child Health J. 2011;15(8):1166-75.

44. Salsberry PJ, Reagan PB. Taking the long view: the prenatal environment and early adolescent overweight. Res Nurs Health. 2007;30(3):297-307.

45. Tan HC, Roberts J, Catov J, Krishnamurthy R, Shypailo R, Bacha F. Mother's pre-pregnancy BMI is an important determinant of adverse cardiometabolic risk in childhood. Pediatric Diabetes. 2015;16(6):419-26.

46. Wen LM, Baur LA, Rissel C, Xu H, Simpson JM. Correlates of body mass index and overweight and obesity of children aged 2 years: findings from the healthy beginnings trial. Obesity (Silver Spring). 2014;22(7):1723-30.

47. Weng SF, Redsell SA, Nathan D, Swift JA, Yang M, Glazebrook C. Estimating overweight risk in childhood from predictors during infancy. Pediatrics. 2013;132(2):e414-21.

48. Whitaker RC. Predicting preschooler obesity at birth: the role of maternal obesity in early pregnancy. Pediatrics. 2004;114(1):e29-36..

49. Wojcicki JM, Young MB, Perham-Hester KA, de Schweinitz P, Gessner BD. Risk factors for obesity at age 3 in Alaskan children, including the role of beverage consumption: results from Alaska PRAMS 2005-2006 and its three-year follow-up survey, CUBS, 2008-2009. PLoS ONE. 2015;10(3):e0118711.

50. Wrotniak BH, Shults J, Butts S, Stettler N. Gestational weight gain and risk of overweight in the offspring at age 7 y in a multicenter, multiethnic cohort study. Am J Clin Nutr. 2008;87(6):1818-24.

51. Zhang J, Himes JH, Guo Y, Jiang J, Yang L, Lu Q, et al. Birth weight, growth and feeding pattern in early infancy predict overweight/obesity status at two years of age: a birth cohort study of Chinese infants. PLoS ONE. 2013;8(6):e64542.

52. Fleten C, Nystad W, Stigum H, Skjaerven R, Lawlor DA, Davey Smith G, et al. Parent-offspring body mass index associations in the Norwegian Mother and Child Cohort Study: a family-based approach to studying the role of the intrauterine environment in childhood adiposity. Am J Epidemiol. 2012;176(2):83-92.

53. Morgen C, Angquist L, Baker J, Andersen A, Michaelsen K, SoRensen T. Prenatal risk factors infuencing childhood BMI and overweight independent of birth weight and infancy BMI - A path analysis within the Danish national birth cohort. Obesity Facts. 2017;10:21-2.

54. Leng J, Li W, Zhang S, Liu H, Wang L, Liu G, et al. GDM Women's Pre-Pregnancy Overweight/Obesity and Gestational Weight Gain on Offspring Overweight Status. PLoS ONE. 2015;10(6):e0129536.
