# Supplementary material for: The association between maternal body mass index and child obesity: A systematic review and meta-analysis
Source: PLoS Med. 2019 Jun 11;16(6):e1002817. doi: 10.1371/journal.pmed.1002817 (PMC6559702; doi:10.1371/journal.pmed.1002817)
Supplement: S8 Table — (DOCX) [file pmed.1002817.s018.docx]

# S8 Table: Nonlinear meta-analyses using cubic splines regression^a^

**Table A: Child overweight/obese (BMI ≥85^th^ percentile)**

| Overall |  | Coef. | Std. err. | z | P | 95% Confidence Interval | |
| --- | --- | --- | --- | --- | --- | --- | --- |
|  | spline1 | 0.1420 | 0.0162 | 8.76 | <0.001 | 0.1102 | 0.1738 |
|  | spline2 | -0.0823 | 0.0140 | -5.86 | <0.001 | -0.1098 | -0.0547 |

**Table B: Child overweight (BMI 85^th^ to 95^th^ percentile)**

| Overall |  | Coef. | Std. err. | z | P | 95% Confidence Interval | |
| --- | --- | --- | --- | --- | --- | --- | --- |
|  | spline1 | 0.0990 | 0.0172 | 5.75 | <0.001 | 0.0652 | 0.1328 |
|  | spline2 | -0.0450 | 0.0080 | -5.62 | <0.001 | -0.0608 | -0.0293 |

**Table C: Child obese (BMI ≥95^th^ percentile)**

| Overall |  | Coef. | Std. err. | z | P | 95% Confidence Interval | |
| --- | --- | --- | --- | --- | --- | --- | --- |
|  | spline1 | 0.1484 | 0.0173 | 8.59 | <0.001 | 0.1145 | 0.1823 |
|  | spline2 | -0.0470 | 0.0108 | -4.34 | <0.001 | -0.0682 | -0.0258 |

**Table D: Child BMI continuous outcome**

| Overall |  | Coef. | Std. err. | z | P | 95% Confidence Interval | |
| --- | --- | --- | --- | --- | --- | --- | --- |
|  | spline1 | 0.1001 | 0.0123 | 8.10 | <0.001 | 0.0759 | 0.1243 |
|  | spline2 | -0.0351 | 0.0303 | -1.16 | 0.246 | -0.0945 | 0.0242 |

**Table E: Child BMI z-score continuous outcome**

| Overall |  | Coef. | Std. err. | z | P | 95% Confidence Interval | |
| --- | --- | --- | --- | --- | --- | --- | --- |
|  | spline1 | 0.0672 | 0.0151 | 4.44 | <0.001 | 0.0375 | 0.0968 |
|  | spline2 | -0.0337 | 0.0204 | -1.65 | 0.099 | -0.0738 | 0.0063 |

Abbreviations: z, value for the z statistic; Coef., coefficient; Std. Err., Standard error; P, p-value.

Footnote:

^a^Nonlinearity was assessed by testing that the coefficient of the second spline was equal to zero.
