# Supplementary material for: The association between maternal body mass index and child obesity: A systematic review and meta-analysis
Source: PLoS Med. 2019 Jun 11;16(6):e1002817. doi: 10.1371/journal.pmed.1002817 (PMC6559702; doi:10.1371/journal.pmed.1002817)
Supplement: S9 Table — (DOCX) [file pmed.1002817.s019.docx]

# S9 Table: Additional data reported that were not included in meta-analysis for child obesity (≥95th percentile)

| **Study** | **Reason for exclusion from meta-analysis^a^** | **Child age** | **Sample size^b^** | **Location** | **Study population (description of duplicate data included in meta-analysis if relevant)** | **Quality score** | **Maternal BMI reference group** | **Maternal underweight: child obesity^c^** | **Maternal overweight: child obesity^c^** | **Maternal obesity: child obesity^c^** | **Continuous maternal BMI: child obesity^c^** |
| --- | --- | --- | --- | --- | --- | --- | --- | --- | --- | --- | --- |
| Ajslev *et al.* 2011[1] | No frequency data provided | 7 | 25356 | Denmark | Danish National Birth Cohort | 6 | Recommended BMI: 0.7% child obesity | Not reported | 2.5% child obesity | 5% child obesity | Not reported |
| Reilly *et al.* 2005[2] | No frequency data provided | 7 | 5493 | UK | ALSPAC Study^d^ | 6 | <30kg/m^2^ | Not reported | Not reported | AOR 4.25 (95%CI 2.86, 6.32) | Not reported |
| Birbilis *et al.* 2013[3] | No frequency data provided | 9 to 13 | 2294 | Greece | Healthy Growth Study | 6 | Recommended BMI not defined | AOR 0.7 (95% CI 0.3, 1.58) | AOR 1.3 (95% CI 0.82, 1.95) | AOR 2.15 (95% CI 1.27, 3.7) | Not reported |
| Weng *et al.* 2013[4] | Multiple ages reported for the same cohort | 3 | 5370 | UK | Millennium Cohort Study (Massion *et al.* 2016[5] included in meta-analysis with this cohort data age 11) | 4 | 18.5-24.9kg/m^2^ | Calculated OR 0.37 (95% CI 0.15, 0.90) | Calculated OR 1.94 (95% CI 1.50, 2.50) | Calculated OR 3.01 (95% CI 2.23, 4.07) | Not reported |
| Rath *et al.* 2016[6] | Multiple ages reported for the same cohort | 3 | 916 | Australia | Western Australian Pregnancy Cohort Study | 5 | <24.9kg/m^2^ | Not reported | OR 3.36 (95% CI 1.59, 6.75) | OR 5.58 (95% CI 2.24, 12.66) | Not reported |
| Rath *et al.* 2016[6] |  | 5 | 1190 |  |  | 5 | <24.9kg/m^2^ | Not reported | OR 2.08 (95% CI 1.00, 4.04) | OR 4.27 (95% CI 2.00, 8.51) | Not reported |
| Salsberry *et al.* 2005[7] | Multiple ages reported for the same cohort | 3 | 3022 | USA | NLSY^d^ | 6 | 18.5-24.9kg/m^2^ | AOR 0.97 (95% CI 0.65, 1.44) | AOR 1.04 (95% CI 0.81, 1.33) | AOR 1.37 (95% CI 1.02, 1.84) | Not reported |
| Salsberry *et al.* 2005[7] |  | 5 | 3022 |  |  | 6 | 18.5-24.9kg/m^2^ | AOR 0.75 (95% CI 0.47, 1.19) | AOR 1.4 (95% CI 1.07, 1.19) | AOR 1.69 (95% CI 1.22, 2.34) | Not reported |
| Li *et al.* 2005[8] | No frequency data provided + multiple ages reported for the same cohort | 7 to 10 | 2490 | USA | NLSY^d^ | 5 | Recommended BMI not defined | Not reported | AOR 2.5 (95%CI 1.8,3.6) | AOR 4.1 (95%CI 2.6, 6.4) | Not reported |
| Salsberry *et al.* 2007[9] |  | 12 to 13 | 3368 |  |  | 6 | <24.9kg/m^2^ | Not reported | AOR 2.18 (95% CI 1.51, 3.13) | AOR 4.28 (95% CI 2.69, 6.83) | Not reported |
| Whitaker 2004[10] | Multiple ages reported for the same cohort | 2 | 6511 | Ohio USA | Special Supplemental Nutrition Program for Women, Infants, and Children | 5 | 18.5-24.10kg/m^2^ | AOR 0.41 (95% CI 0.21, 0.81) | AOR 1.42 (95% CI 1.13, 1.79) | Class I+II: AOR 2.28 (95% CI 1.84, 2.83); Class III: AOR 3.05 (95% CI 2.22, 4.18) | Not reported |
| Whitaker 2004[10] |  | 3 | 5743 |  |  | 5 | 18.5-24.11kg/m^2^ | AOR 0.63 (95% CI 0.36, 1.1) | AOR 1.69 (95% CI 1.35, 2.1) | Class I+II: AOR 3.06 (95% CI 2.49, 3.76); Class III: AOR 3.82 (95% CI 2.8, 5.19) | Not reported |
| Additional studies identified in the updated searches March 2019 | | | | | | | | | | | |
| Bridgman et al. 2018[11] | Study identified in updated search | 1 | 955 | Canada | Canadian Healthy Infant Longitudinal Development (CHILD) Study | 7 | 18.5-24.9kg/m^2^ | Not reported | AOR 2.51 (95% CI 1.11, 5.67) | AOR 2.62 (95% CI 1.10, 6.22) | Not reported |
| Kjaer *et al.* 2019[12] | Study identified in updated search | 9 | 53 | USA | University of California, San Francisco Medical Center and San Francisco General Hospital | 7 | Continuous | Not reported | Not reported | Not reported | AOR 1.09 (95% CI 1.00, 1.20) |
| Wallby *et al.* 2017[13] | Study identified in updated search | 4 | 30,508 | Sweden | Population: Uppsala and Orebro, Sweden | 7 | Normal Weight | AOR 0.42 (95% CI 0.13, 1.29) | AOR 1.80 (95% CI 1.45, 2.22) | AOR 3.78 (95% CI 3.03, 4.70) | Not reported |

Abbreviations: BMI, body mass index; (A)OR, (adjusted) odds ratio; CI, confidence interval.

Footnote:

^a^Summary of reasons for exclusion from the meta-analysis: Three studies did not report the frequency data required and children from these cohorts were not included in the meta-analysis[1-3]. Six studies reported duplicate cohort data for children already included in the meta-analysis at different ages[4, 6-10]. Three studies identified in the updated search [11-13].

^b^Sample size included in the analysis reported in the table rather than sample size of the entire cohort/study population.

^c^Summary of associations between maternal BMI and child obesity: 11 studies[1-4, 6-10] reported 16 associations with maternal obesity, all ORs were statistically significant ranging from 1.37 to 5.58 (meta-analysis result for comparison 3.64, 95% CI 2.68, 4.95). 10 studies[1, 3, 4, 6-10] reported 13 associations between child obesity and maternal overweight with ORs ranging from 1.04 to 3.36; 10 were statistically significant (meta-analysis result for comparison 1.89, 95% CI 1.62, 2.19). Five studies[3, 4, 7, 10] reported seven associations between maternal underweight and child obesity. ORs ranged from 0.37 to 0.97, only two were statistically significant.

^d^Abbreviated cohort names, for full cohort names see S4 Table.

**References:**

1. Ajslev TA, Andersen CS, Gamborg M, Sorensen TI, Jess T. Childhood overweight after establishment of the gut microbiota: the role of delivery mode, pre-pregnancy weight and early administration of antibiotics. Int J Obes (Lond). 2011;35(4):522-9.

2. Reilly JJ, Armstrong J, Dorosty AR, Emmett PM, Ness A, Rogers I, et al. Early life risk factors for obesity in childhood: cohort study. BMJ. 2005;330(7504):1357.

3. Birbilis M, Moschonis G, Mougios V, Manios Y, Healthy Growth Study g. Obesity in adolescence is associated with perinatal risk factors, parental BMI and sociodemographic characteristics. Eur J Clin Nutr. 2013;67(1):115-21.

4. Weng SF, Redsell SA, Nathan D, Swift JA, Yang M, Glazebrook C. Estimating overweight risk in childhood from predictors during infancy. Pediatrics. 2013;132(2):e414-21.

5. Massion S, Wickham S, Pearce A, Barr B, Law C, Taylor-Robinson D. Exploring the impact of early life factors on inequalities in risk of overweight in UK children: findings from the UK Millennium Cohort Study. Archives of disease in childhood. 2016. Epub 2016/05/11.

6. Rath SR, Marsh JA, Newnham JP, Zhu K, Atkinson HC, Mountain J, et al. Parental pre-pregnancy BMI is a dominant early-life risk factor influencing BMI of offspring in adulthood. Obesity Science and Practice. 2016;2(1):48-57.

7. Salsberry PJ, Reagan PB. Dynamics of early childhood overweight. Pediatrics. 2005;116(6):1329-38.

8. Li C, Kaur H, Choi WS, Huang TT, Lee RE, Ahluwalia JS. Additive interactions of maternal prepregnancy BMI and breast-feeding on childhood overweight. Obes Res. 2005;13(2):362-71.

9. Salsberry PJ, Reagan PB. Taking the long view: the prenatal environment and early adolescent overweight. Res Nurs Health. 2007;30(3):297-307.

10. Whitaker RC. Predicting preschooler obesity at birth: the role of maternal obesity in early pregnancy. Pediatrics. 2004;114(1):e29-36.

11. Bridgman SL, Azad MB, Persaud RR, Chari RS, Becker AB, Sears MR, et al. Impact of maternal pre‐pregnancy overweight on infant overweight at 1 year of age: associations and sex‐specific differences. Pediatric Obesity. 2018;13(10):579-89.

12. Kjaer TW, Faurholt-Jepsen D, Medrano R, Elwan D, Mehta K, Christensen VB, et al. Higher birthweight and maternal pre-pregnancy BMI persist with obesity association at age 9 in high risk Latino children. Journal of Immigrant and Minority Health. 2019:1-9.

13. Wallby T, Lagerberg D, Magnusson M. Relationship between breastfeeding and early childhood obesity: Results of a prospective longitudinal study from birth to 4 years. Breastfeeding Medicine. 2017;12(1):48-53.
