# Supplementary material for: The association between maternal body mass index and child obesity: A systematic review and meta-analysis
Source: PLoS Med. 2019 Jun 11;16(6):e1002817. doi: 10.1371/journal.pmed.1002817 (PMC6559702; doi:10.1371/journal.pmed.1002817)
Supplement: S10 Table — (DOCX) [file pmed.1002817.s020.docx]

# S10 Table: Additional data reported that were not included in meta-analysis for child overweight or obesity (≥85th percentile)

| **Study** | **Reason for exclusion from meta-analysis^a^** | **Child age** | **Sample size^b^** | **Location** | **Study population (description of duplicate data included in meta-analysis if relevant)** | **Quality score** | **Maternal BMI reference group** | **Categorical maternal overweight: child OW/OB^c^** | **Categorical maternal obesity: child OW/OB^c^** | **Continuous maternal BMI: child OW/OB^c^** |
| --- | --- | --- | --- | --- | --- | --- | --- | --- | --- | --- |
| de Hoog *et al.* 2011[1] | Multiple ages reported for the same cohort | 2 | 3156 | Netherlands | ABCD Study^d^ (Gademan *et al.* 2014[2] included in meta-analysis with this cohort data age 5) | 5 | Continuous BMI | Not reported | Not reported | OR 1.09 (95% CI 1.07, 1.12) |
| Mamun *et al.* 2005[3] | Multiple ages reported for the same cohort | 5 | 2758 | Australia | Mater-University Study of Pregnancy (O Callaghan *et al.* 1997[4] included in meta-analysis with this cohort data age 5, reporting 3 maternal BMI categories which enabled inclusion in nonlinear meta-analysis) | 7 | Categorical BMI 18.50 - 24.99kg/m^2^ | Calculated OR 2.23 (95% CI 1.76, 2.83) | | Not reported |
| Mamun *et al.* 2005[3] |  | 14 | 2758 |  |  | 7 | Categorical BMI 18.50 - 24.99kg/m^2^ | Calculated OR 4.00 (95% CI 3.24, 4.93) | | Not reported |
| Morgen *et al.* 2017[5] | Exposure maternal BMI z-score not BMI | 7 | 39398 | Denmark | Danish National Birth Cohort | 5 | Continuous BMI z-score | Not reported | Not reported | AOR 1.54 (95% CI 1.47, 1.61) |
| Morgen *et al.* 2017[5] |  | 11 | 23159 |  |  | 5 | Continuous BMI z-score | Not reported | Not reported | AOR 1.60 (95% CI 1.48, 1.72) |
| Ajslev *et al.* 2011[6] | No frequency data provided | 7 | 28344 |  |  | 6 | Categorical BMI <25kg/m^2^ | AOR 1.13 (95% CI 1.12, 1.14) | | Not reported |
| Olson *et al.* 2009[7] | Multiple ages reported for the same cohort | 3 | 208 | USA | Bassett Mothers Health Project (Olson *et al.* 2009[7] included in meta-analysis with this cohort data age 4) | 7 | Both continuous BMI and categorical BMI <26kg/m^2^ | Calculated OR 2.09 (95% CI 1.14, 3.81) | | AOR 1.109 (95% CI not reported) |
| Weng *et al.* 2013[8] | Multiple ages reported for the same cohort | 3 | 6337 | UK | Millennium Cohort Study (Massion *et al.* 2016[9] included in meta-analysis with this cohort data age 11) | 4 | <18.5kg/m^2^ | AOR 2.35 (95% CI 1.60, 3.47) | AOR 2.98 (95% CI 1.98, 4.47) | Not reported |
| Rooney *et al.* 2011[10] | Multiple ages reported for the same cohort | 4 to 5 | 359 | USA | Not reported, large healthcare system from Midwest USA | 7 | Categorical BMI <30kg/m^2^ | Not reported | Calculated OR 4.59 (95% CI 2.22, 9.49) | Not reported |
| Rathnayake *et al.* 2013[11] | No frequency data provided | 3 to 5 | 142 | Sri Lanka | Not report, sample form 16 schools | 6 | Categorical BMI <27.5kg/m^2^ | Not reported | AOR 3.96 (95% CI 1.46, 10.76) | Not reported |
| Bider-Canfield *et al.* 2017[12] | No frequency data provided | 2 | 15710 | USA | Kaiser Permante Southern California | 8 | Categorical BMI <25kg/m^2^ | AOR 1.46 (95% CI 1.30, 1.63) | AOR 2.21 (95% CI 1.97, 2.48) | Not reported |
| Pham *et al.* 2013[13] | No frequency data provided | 2 to 4 | 2491 | USA | Kaiser Permanente Northern California | 7 | Categorical BMI 18.5-24.9kg/m^2^ | AOR 1.4 (95% CI 1.1, 1.8) | AOR 2.5 (95% CI 1.9, 3.4) | Not reported |
| Kitsantas *et al.* 2010[14] | Multiple ages reported for the same cohort and missing frequency data | 2 | 2270 | USA | Early Childhood Longitudinal Study-Birth Cohort (Hinkle *et al.* 2012[15] included in meta-analysis with this cohort data age 5) | 4 | Categorical BMI 19.8-26kg/m^2^ | White ethnic group: AOR 1.33 (95% CI 1.03, 1.71); Hispanic ethnic group AOR 1.69 (95% CI 1.08, 2.67) | | Not reported |
| Kitsantas *et al.* 2010[14] |  | 4 | 3650 |  |  |  | Categorical BMI 19.8-26kg/m^2^ | White ethnic group: AOR 1.42 (95% CI 1.05, 1.93); Hispanic ethnic group AOR 2.74 (95% CI 1.60, 4.69) | | Not reported |
| Additional studies identified in the updated searches March 2019 | | | | | | | | | | |
| Androutsos *et al.* 2018[16] | Study identified in updated search | 4 to 6 | 7541 | Multiple (Belgium, Bulgaria, Germany, Greece, Poland, and Spain) | ToyBox study | 6 | Categorical BMI ≤24.9kg/m^2^ | AOR 1.52 (95% CI 1.10, 2.09) | | Not reported |
| Aris *et al.* 2018[17] | Study identified in updated search | 2 | 858 | Singapore | Growing Up in Singapore Towards healthy Outcomes (GUSTO) Study | 7 | Categorical BMI <25kg/m^2^ | Predicted probability 11.8% (95% CI 9.8%, 13%) | | Not reported |
| Iguacel *et al.* 2018[18] | Study identified in updated search | 6 | 1031 | Spain | Growth and Feeding during Infancy and Early Childhood in Aragon (CALINA) Study | 6 | <25kg/m^2^ | AOR 1.91 (95% CI 1.38, 2.66) | AOR 2.20 (95% CI 1.41, 3.42) | Not reported |
| Mao *et al.* 2017[19] | Study identified in updated search | 2-9 | 1,446 | USA | Boston Birth Cohort | 8 | Categorical BMI 18.5-24.9kg/m^2^ | ARR 1.3 (95% CI 1.2, 1.6) | ARR 1.6 (95% CI 1.3, 1.8) | ARR 1.3 (95% CI 1.1,1.4) |
| Toftemo *et al.* 2018[20] | Study identified in updated search | 4-5 | 570 | Norway | STORK Groruddalen | 7 | Categorical BMI 18.5-24.9kg/m^2^ | AOR 2.56 (95% CI 1.52, 4.61) | | Not reported |
| Zhang *et al.* 2018[21] | Study identified in updated search | 1 | 3764 | China | Population: Affiliated Hospital of Jining Medical University, China | 7 | Categorical BMI 18.5-23.9kg/m^2^ | ARR 1.36 (95% CI 1.19, 1.53) | ARR 1.58 (95% CI 1.16, 1.96) | Not reported |

Abbreviations: BMI, body mass index; OW, overweight; OB, obesity; (A)OR, (adjusted) odds ratio; CI, confidence interval.

Footnote:

^a^Summary of reasons for exclusion from the meta-analysis: Four studies did not report the frequency data required and children from these cohorts were not included in the meta-analysis[6, 11-13]. Six studies reported duplicate cohort data for children already included in the meta-analysis at different ages[1, 3, 7, 8, 10, 14]. One study[5] reported associations between child overweight and obesity and maternal BMI z-score and therefore the units of exposure were not comparable to the studies included in the meta-analysis which reported maternal BMI. Six studies were identified in the updated search [16-21].

^b^Sample size included in the analysis reported in the table rather than sample size of the entire cohort/study population.

^c^Summary of associations between maternal BMI and child overweight or obesity: Eight studies[8, 10-13, 18, 19, 21] reported associations with maternal obesity, with statistically significant ORs ranging from 1.58 to 4.59 (meta-analysis result for comparison OR 2.69, 95% CI 2.10, 3.46). Six studies[8, 12, 13, 18, 19, 21] reported associations with maternal overweight, with statistically significant increased odds ranging from 1.4 to 2.35 (meta-analysis result 1.65, 95% CI 1.47, 1.85). Seven cohorts[3, 6, 7, 14, 16, 17, 20] reported associations with maternal BMI≥25kg/m^2^ all were statistically significant ORs between 1.13 to 4.00. Four studies[1, 5, 7, 19] also reported significant ORs for child overweight or obesity with continuous maternal BMI ranging between 1.09 and 1.60 (meta-analysis result for comparison 1.55, 95% CI 1.43, 1.69).

^d^Abbreviated cohort names, for full cohort names see S4Table

**References:**

1. de Hoog ML, van Eijsden M, Stronks K, Gemke RJ, Vrijkotte TG. Overweight at age two years in a multi-ethnic cohort (ABCD study): the role of prenatal factors, birth outcomes and postnatal factors. BMC Public Health. 2011;11(1):611.

2. Gademan MG, Vermeulen M, Oostvogels AJ, Roseboom TJ, Visscher TL, van Eijsden M, et al. Maternal prepregancy BMI and lipid profile during early pregnancy are independently associated with offspring's body composition at age 5-6 years: the ABCD study. PLoS ONE. 2014;9(4):e94594.

3. Mamun AA, Lawlor DA, O'Callaghan MJ, Williams GM, Najman JM. Family and early life factors associated with changes in overweight status between ages 5 and 14 years: findings from the Mater University Study of Pregnancy and its outcomes. Int J Obes (Lond). 2005;29(5):475-82.

4. O'Callaghan MJ, Williams GM, Andersen MJ, Bor W, Najman JM. Prediction of obesity in children at 5 years: a cohort study. J Paediatr Child Health. 1997;33(4):311-6.

5. Morgen C, Angquist L, Baker J, Andersen A, Michaelsen K, SoRensen T. Prenatal risk factors infuencing childhood BMI and overweight independent of birth weight and infancy BMI - A path analysis within the Danish national birth cohort. Obesity Facts. 2017;10:21-2.

6. Ajslev TA, Andersen CS, Gamborg M, Sorensen TI, Jess T. Childhood overweight after establishment of the gut microbiota: the role of delivery mode, pre-pregnancy weight and early administration of antibiotics. Int J Obes (Lond). 2011;35(4):522-9.

7. Olson CM, Strawderman MS, Dennison BA. Maternal weight gain during pregnancy and child weight at age 3 years. Matern Child Health J. 2009;13(6):839-46.

8. Weng SF, Redsell SA, Nathan D, Swift JA, Yang M, Glazebrook C. Estimating overweight risk in childhood from predictors during infancy. Pediatrics. 2013;132(2):e414-21.

9. Massion S, Wickham S, Pearce A, Barr B, Law C, Taylor-Robinson D. Exploring the impact of early life factors on inequalities in risk of overweight in UK children: findings from the UK Millennium Cohort Study. Archives of Disiease in Childhood. 2016.

10. Rooney BL, Mathiason MA, Schauberger CW. Predictors of obesity in childhood, adolescence, and adulthood in a birth cohort. Matern Child Health J. 2011;15(8):1166-75.

11. Rathnayake KM, Satchithananthan A, Mahamithawa S, Jayawardena R. Early life predictors of preschool overweight and obesity: a case-control study in Sri Lanka. BMC Public Health. 2013;13:994.

12. Bider-Canfield Z, Martinez MP, Wang X, Yu W, Bautista MP, Brookey J, et al. Maternal obesity, gestational diabetes, breastfeeding and childhood overweight at age 2 years. Pediatric Obesity. 2017;12(2):171-8.

13. Pham MT, Brubaker K, Pruett K, Caughey AB. Risk of childhood obesity in the toddler offspring of mothers with gestational diabetes. Obstet Gynecol. 2013;121(5):976-82.

14. Kitsantas P, Pawloski LR, Gaffney KF. Maternal prepregnancy body mass index in relation to Hispanic preschooler overweight/obesity. Eur J Pediatr. 2010;169(11):1361-8.

15. Hinkle SN, Sharma AJ, Swan DW, Schieve LA, Ramakrishnan U, Stein AD. Excess gestational weight gain is associated with child adiposity among mothers with normal and overweight prepregnancy weight status. J Nutr. 2012;142(10):1851-8.

16. Androutsos O, Moschonis G, Ierodiakonou D, Karatzi K, De Bourdeaudhuij I, Iotova V, et al. Perinatal and lifestyle factors mediate the association between maternal education and preschool children's weight status: the ToyBox study. Nutrition. 2018;48:6-12.

17. Aris IM, Bernard JY, Chen LW, Tint MT, Pang WW, Soh S-E, et al. Modifiable risk factors in the first 1000 days for subsequent risk of childhood overweight in an Asian cohort: significance of parental overweight status. Int J Obes (Lond). 2018;42(1):44.

18. Iguacel I, Escartín L, Fernández-Alvira JM, Iglesia I, Labayen I, Moreno LA, et al. Early life risk factors and their cumulative effects as predictors of overweight in Spanish children. International Journal of Public Health. 2018;63(4):501-12.

19. Mao G, Nachman RM, Sun Q, Zhang X, Koehler K, Chen Z, et al. Individual and Joint Effects of Early-Life Ambient PM 2.5 Exposure and Maternal Prepregnancy Obesity on Childhood Overweight or Obesity. Environmental Health Perspectives. 2017;125(6):067005.

20. Toftemo I, Jenum AK, Lagerløv P, Júlίusson PB, Falk RS, Sletner L. Contrasting patterns of overweight and thinness among preschool children of different ethnic groups in Norway, and relations with maternal and early life factors. BMC Public Health. 2018;18(1):1056.

21. Zhang W, Niu F, Ren X. Association of maternal pre‐pregnancy body mass index and gestational weight gain with Chinese infant growth. Journal of Paediatrics and Child Health. 2018.
