# Supplementary material for: The association between maternal body mass index and child obesity: A systematic review and meta-analysis
Source: PLoS Med. 2019 Jun 11;16(6):e1002817. doi: 10.1371/journal.pmed.1002817 (PMC6559702; doi:10.1371/journal.pmed.1002817)
Supplement: S11 Table — (DOCX) [file pmed.1002817.s021.docx]

# S11 Table: Additional data reported that were not included in meta-analysis for child overweight (85th to 95th percentile)

| **Study** | **Reason for exclusion from meta-analysis^a^** | **Child age** | **Sample size^b^** | **Location** | **Study population (description of duplicate data included in meta-analysis if relevant)** | **Quality score** | **Maternal BMI reference group** | **Maternal underweight: child overweight^c^** | **Maternal overweight: child overweight^c^** | **Maternal obesity: child overweight^c^** |
| --- | --- | --- | --- | --- | --- | --- | --- | --- | --- | --- |
| Weng *et al.* 2013 [1] | Multiple ages reported for the same cohort | 3 | 6337 | UK | Millennium Cohort Study (Massion *et al.* 2016[2] included in meta-analysis with this cohort data age 11) | 4 | 18.5-24.9kg/m^2^ | Calculated OR 0.50 (95% CI 0.35, 0.72) | Calculated OR 1.61 (95% CI 1.41, 1.85) | Calculated OR 2.11 (95% CI 1.76, 2.51) |
| Durmus *et al.* 2012 [2] | Multiple ages reported for the same cohort | 4 | 3510 | Netherlands | Generation R Study (Gaillard *et al.* 2004[4] included in meta-analysis with this cohort data age 6) | 7 | <25kg/m^2^ | Not reported | Calculated OR 1.56 (95% CI 1.21, 2.01) | Calculated OR 2.29 (95% CI 1.63, 3.21) |
| Janjua *et al.* 2012 [3] | No frequency data provided | 5 | 615 | USA | Longitudinal Study of Pregnancy Outcomes and Childhood Psychomotor Development | 6 | <24.9kg/m^2^ | Not reported | ARR 1.9 (95% CI 1.34, 2.70) | ARR 1.88 (95% CI 1.33, 2.65) |
| Birbilis *et al.* 2013 [4] | No frequency data provided | 9 to 13 | 2294 | Greece | Healthy Growth Study | 6 | "Normal weight" not defined | AOR 0.47 (95% CI 0.29, 0.74) | AOR 0.92 (95% CI 0.66, 1.30) | AOR 1.26 (95% CI 0.66, 2.40) |

Abbreviations: BMI, body mass index; (A)OR, (adjusted) odds ratio; (A)RR. (adjusted) risk ratio; CI, confidence interval.

Footnote:

^a^Summary of reasons for exclusion from the meta-analysis: Two studies did not report the frequency data required and children from these cohorts were not included in the meta-analysis[3, 4]. Two studies reported duplicate cohort data for children already included in the meta-analysis at different ages[1,2].

^b^Sample size included in the analysis reported in the table rather than sample size of the entire cohort/study population.

^c^Summary of associations between maternal BMI and child overweight: Four studies[1, 2, 3, 4] reported significantly increased odds for maternal obesity ranging between 1.26 and 2.29 (meta-analysis result for comparison 1.80, 95% CI 1.25, 2.59). All four studies reported maternal overweight, and three found significantly increased associations with child overweight[1, 2, 4]. Two [1, 4] reported significantly reduced ORs for child overweight with maternal underweight.

**References:**

1. Weng SF, Redsell SA, Nathan D, Swift JA, Yang M, Glazebrook C. Estimating overweight risk in childhood from predictors during infancy. Pediatrics. 2013;132(2):e414-21.

2. Durmus B, Arends LR, Ay L, Hokken-Koelega AC, Raat H, Hofman A, et al. Parental anthropometrics, early growth and the risk of overweight in pre-school children: the Generation R Study. Pediatric Obesity. 2012;8(5):339-50.

3. Janjua NZ, Mahmood B, Islam MA, Goldenberg RL. Maternal and early childhood risk factors for overweight and obesity among low-income predominantly black children at age five years: A prospective cohort study. Journal of Obesity. 2012;457173.

4. Birbilis M, Moschonis G, Mougios V, Manios Y, Healthy Growth Study g. Obesity in adolescence is associated with perinatal risk factors, parental BMI and sociodemographic characteristics. Eur J Clin Nutr. 2013;67(1):115-21.
