# Supplementary material for: The association between maternal body mass index and child obesity: A systematic review and meta-analysis
Source: PLoS Med. 2019 Jun 11;16(6):e1002817. doi: 10.1371/journal.pmed.1002817 (PMC6559702; doi:10.1371/journal.pmed.1002817)
Supplement: S12 Table — (DOCX) [file pmed.1002817.s022.docx]

# S12 Table: Additional data for narrative overview for continuous child BMI and z-score outcomes (categorical maternal BMI exposure)

| **Study** | **Reason for exclusion from meta-analysis^a^** | **Child age** | **Sample size^b^** | **Location** | **Study population (description of duplicate data included in meta-analysis if relevant)** | **Unit of child outcome** | **Maternal underweight: mean (SD) child BMI/z-score^c^** | **Maternal recommended weight: mean (SD) child BMI/z-score^c^** | **Maternal overweight: mean (SD) child BMI/z-score^c^** | **Maternal obesity: mean (SD) child BMI/z-score^c^** | **P value** |
| --- | --- | --- | --- | --- | --- | --- | --- | --- | --- | --- | --- |
| Andres *et al.* 2015[1] | Multiple ages reported for the same cohort | 1 | 72 | USA | Beginnings Study (oldest age 6 used) | BMI z-score | Not reported | 0.52 (0.15) | 0.80 (0.13) | 0.86 (0.14) | NR |
|  |  | 2 | 83 |  |  |  |  | 0.55 (0.12) | 0.73 (0.12) | 0.70 (0.12) |  |
|  |  | 3 | 71 |  |  |  |  | 0.30 (0.10) | 0.44 (0.13) | 0.58 (0.11) |  |
|  |  | 4 | 148 |  |  |  |  | 0.16 (0.10) | 0.37 (0.12) | 0.81 (0.11) |  |
|  |  | 5 | 173 |  |  |  |  | 0.19 (0.11) | 0.51 (0.13) | 0.90 (0.12) |  |
| Berkowitz *et al.* 2005[2] | Multiple ages reported for the same cohort | 4 | 70 | USA | Infant Growth Study (oldest age 6 used) | BMI | Not reported | 15.7 (1.00) | 16.5 (2.00) | | NR |
| Jharap *et al.* 2017[3] | Multiple ages reported for the same cohort | 2 | 659 | Netherlands | Generation R (Toemen *et al.* 2016[4] included in meta-analysis with this cohort data age 6) | BMI | 15.6 (1.4) | 15.8 (1.2) | 16.1 (1.3) | 16.6 (1.4) | NR |
| Toemen *et al.* 2016[4] | Multiple ages reported for the same cohort | 6 | 4852 | Netherlands | Generation R (study reported both child BMI and z-score outcome for age 6, BMI included in meta-analysis) | BMI z-score | -0.14 (0.84) | 0.17 (0.86) | 0.50 (0.95) | 0.95 (1.10) | NR |
| Leng *et al.* 2015[5] | BMI and z-score reported for same cohort | 1 to 5 | 1263 | China | Tianjin GDM screening project (reported both BMI and z-score, BMI included) | z-score | 0.38 (SE 0.08) | | 0.54 (SE 0.09) | 0.73 (SE 0.12) | NR |
| Wen *et al.* 2014[6] | Beta and 95% CI reported rather than mean (SD) | 2 | 242 | Australia | Healthy Beginnings Trial | BMI | 1 | | Beta 0.87 (95% CI 0.41, 1.33) | | NR |
| Ehrenthal *et al.* 2013[7] | Beta and 95% CI reported rather than mean (SD) | 4 | 3302 | USA | Delaware Mother Baby Cohort | z-score | Beta -0.354 (95% CI -0.054, -0.164) | 0 | Beta 0.261 (95% CI 0.169, 0.355) | Class I/II Beta 0.497 (95% CI 0.382, 0.611); Class III Beta 0.755 (95% CI 0.636, 0.874) | NR |
| Jin *et al.* 2016[8] | Data only presented in a figure with no corresponding values | 20, 30 and 40 months | 826 | China | Zhejiang Province of China | z-score | Child z-score plot lowest at all ages for maternal underweight | Child z-score plot in between maternal underweight and maternal overweight at all ages | Child z-score significantly increased at all ages compared with maternal recommended BMI (p<0.001) | | <0.001 |
| Basatemur *et al.* 2013[9] | Mean reported but no SD | 5 | 16993 | UK | Millennium Cohort Study | z-score | 0.04 | 0.36 | 0.70 | 1.03 | <0.05 |
|  |  | 7 | 16993 |  |  |  | -0.06 | 0.30 | 0.70 | 1.11 | <0.05 |
| Stamnes Kopp *et al.* 2012[10] | Mean reported but no SD | 3 | 31169 | Norway | Norwegian Mother and Child Cohort Study | BMI | 15.7 | 16.1 | 16.2 | Class I 16.4; Class II 16.4; Class III 16.5 | <0.001 |
| Additional studies identified in the updated searches March 2019 | | | | | | | | | | | |
| Mintjens et al. 2018[11] | Study identified in updated search + multiple ages reported for the same cohort | 8-9 | 194 | Netherlands | ABCD Study | z-score | 16.0 (1.4) | | 16.5 (1.6) | | 0.10 |
| Zheng et al. 2019[12] | Study identified in updated search | 3 to 42 months | 3065 | Australia | Melbourne Infant Feeding Activity and Nutrition Trial (InFANT) Program and the InFANT Extend Program | z-score | 0 | | Adjusted Beta 0.17 (95% CI 0.07,0.28) | | NR |

Abbreviations: BMI, body mass index; SD, standard deviation; NR, Not Reported.

Footnote:

^a^Summary of reasons for exclusion from the meta-analysis: Five studies did not report all of the data required and children from these cohorts were not included in the meta-analysis[6-10]. Five studies reported duplicate cohort data for children already included in the meta-analysis at different ages, or where the study reported both continuous BMI and z-score outcomes then BMI was included and z-score excluded[1-5]. Two studies were identified after the updated search [11, 12]

^b^Sample size included in the analysis reported in the table rather than sample size of the entire cohort/study population.

^c^Summary of associations between maternal BMI categories and continuous child BMI or z-score: additional data were available for associations between continuous child BMI or z-score outcome between ages 1 and 9 and categorical maternal BMI but were not included in the meta-analysis. Overall, these studies reported an increasing child BMI or z-score as maternal BMI increased[1-12].

**References:**

1. Andres A, Hull HR, Shankar K, Casey PH, Cleves MA, Badger TM. Longitudinal body composition of children born to mothers with normal weight, overweight, and obesity. Obesity (Silver Spring). 2015;23(6):1252-8.

2. Berkowitz RI, Stallings VA, Maislin G, Stunkard AJ. Growth of children at high risk of obesity during the first 6 y of life: implications for prevention. Am J Clin Nutr. 2005;81(1):140-6.

3. Jharap VV, Santos S, Steegers EAP, Jaddoe VWV, Gaillard R. Associations of maternal obesity and excessive weight gain during pregnancy with subcutaneous fat mass in infancy. Early Human Development. 2017;108:23-8.

4. Toemen L, Gishti O, Van Osch-Gevers L, Steegers EAP, Helbing WA, Felix JF, et al. Maternal obesity, gestational weight gain and childhood cardiac outcomes: Role of childhood body mass index. Int J Obes. 2016;40(7):1070-8.

5. Leng J, Li W, Zhang S, Liu H, Wang L, Liu G, et al. GDM Women's Pre-Pregnancy Overweight/Obesity and Gestational Weight Gain on Offspring Overweight Status. PLoS ONE. 2015;10(6):e0129536.

6. Wen LM, Baur LA, Rissel C, Xu H, Simpson JM. Correlates of body mass index and overweight and obesity of children aged 2 years: findings from the healthy beginnings trial. Obesity. 2014;22(7):1723-30.

7. Ehrenthal DB, Maiden K, Rao A, West DW, Gidding SS, Bartoshesky L, et al. Independent relation of maternal prenatal factors to early childhood obesity in the offspring. Obstet Gynecol. 2013;121(1):115-21.

8. Jin WY, Lv Y, Bao Y, Tang L, Zhu ZW, Shao J, et al. Independent and combined effects of maternal prepregnancy body mass index and gestational weight gain on offspring growth at 0-3 years of age. BioMed Research International. 2016;4720785.

9. Basatemur E, Gardiner J, Williams C, Melhuish E, Barnes J, Sutcliffe A. Maternal prepregnancy BMI and child cognition: a longitudinal cohort study. Pediatrics. 2013;131(1):56-63.

10. Stamnes Kopp UM, Dahl-Jorgensen K, Stigum H, Frost Andersen L, Naess O, Nystad W. The associations between maternal pre-pregnancy body mass index or gestational weight change during pregnancy and body mass index of the child at 3 years of age. Int J Obes. 2012;36(10):1325-31.

11. Mintjens S, Gemke RJBJ, van Poppel MNM, Vrijkotte TGM, Roseboom TJ, van Deutekom AW. Maternal Prepregnancy Overweight and Obesity Are Associated with Reduced Physical Fitness But Do Not Affect Physical Activity in Childhood: The Amsterdam Born Children and Their Development Study. Childhood Obesity. 2018;15(1):31-9.

12. Zheng M, Bowe SJ, Hesketh KD, Bolton K, Laws R, Kremer P, et al. Relative effects of postnatal rapid growth and maternal factors on early childhood growth trajectories. Paediatric and Perinatal Epidemiology. 2019.
