# Supplementary material for: The association between maternal body mass index and child obesity: A systematic review and meta-analysis
Source: PLoS Med. 2019 Jun 11;16(6):e1002817. doi: 10.1371/journal.pmed.1002817 (PMC6559702; doi:10.1371/journal.pmed.1002817)
Supplement: S13 Table — (DOCX) [file pmed.1002817.s023.docx]

# S13 Table: Additional data for narrative overview for continuous child BMI and z-score outcomes

| **Study** | **Child age** | **Sample size^a^** | **Location** | **Study population (description of duplicate data included in meta-analysis if relevant)** | **Quality score** | **Unit of maternal exposure** | **Unit of child outcome** | **Coefficients^b^** |
| --- | --- | --- | --- | --- | --- | --- | --- | --- |
| Davey Smith *et al.* 2007[1] | 7 | 4654 | UK | ALSPAC Study^c^ | 5 | BMI z-score | BMI z-score | Adjusted 0.26 (95% CI 0.23, 0.29) |
| Davey Smith *et al.* 2007[1] | 7 | 4654 |  |  | 5 | BMI | BMI | 0.138 (95% CI 0.124, 0.153) |
| Durmus *et al.* 2012[2] | 1 | 4116 | Netherlands | Generation R Study | 7 | BMI z-score | BMI | Adjusted 0.09 (95% CI 0.06, 0.13) |
| Durmus *et al.* 2012[2] | 2 | 4116 |  |  | 7 | BMI z-score | BMI | Adjusted 0.14 (95% CI 0.11, 0.18) |
| Durmus *et al.* 2012[2] | 3 | 4116 |  |  | 7 | BMI z-score | BMI | Adjusted 0.15 (95% CI 0.11, 0.19) |
| Durmus *et al.* 2012[2] | 4 | 4116 |  |  | 7 | BMI z-score | BMI | Adjusted 0.17 (95% CI 0.13, 0.21) |
| Gaillard *et al.* 2014[3] | 6 | 4871 |  |  | 7 | BMI z-score | BMI z-score | Adjusted 0.16 (95% CI 0.13, 0.19) |
| Ehrenthal *et al.* 2013[4] | 4 | 3302 | USA | Delaware Mother Baby Cohort | 7 | BMI | BMI z-score | Adjusted 0.050 (95% CI 0.044, 0.056) |
| Gademan *et al.* 2014[5] | 5 | 1727 | Netherlands | ABCD Study^c^ | 6 | BMI | BMI | Adjusted 0.1 (95% CI 0.08, 0.12) |
| Mesman *et al.* 2009[6] | 1 | 3171 |  |  | 6 | BMI | BMI | Adjusted 0.03 (95% CI 0.02, 0.43) |
| Jacota *et al.* 2016[7] | 5 to 6 | 1035 | France | EDEN mother–child cohort | 5 | BMI | BMI z-score | Adjusted 0.65 (SE 0.20) |
| Knight *et al.* 2007[8] | 1 | 427 | UK | EFSOCH Study^c^ | 5 | BMI z-score | BMI z-score | Adjusted 0.12 (SE 0.05) |
| Knight *et al.* 2007[8] | 2 | 427 |  |  | 5 | BMI z-score | BMI z-score | Adjusted 0.11 (SE 0.053) |
| Li *et al.* 2017[9] | 0 to 5 | 436 | Canada | FAMILY Cohort^c^ | 7 | BMI | BMI z-score | Adjusted 0.025 (SE 0.005) |
| Morgen *et al.* 2017[10] | 7 | 29374 | Denmark | Danish National Birth Cohort | 5 | BMI z-score | BMI z-score | Adjusted 0.21 (95% CI 0.20, 0.22) |
| Morgen *et al.* 2017[10] | 11 | 18044 |  |  | 5 | BMI z-score | BMI z-score | Adjusted 0.29 (95% CI 0.27, 0.30) |
| Sorensen *et al.* 2016[11] | 1 | 30,566 |  |  | 6 | BMI | BMI z-score | Adjusted 0.075 (95% CI 0.061, 0.089) |
| Terry *et al.* 2011[12] | 7 | 20523 | USA | Collaborative Perinatal Project | 7 | BMI | BMI | Adjusted 0.07 (SE 0.004) |
| Wen *et al.* 2014[13] | 2 | 242 | Australia | Healthy Beginnings Trial | 6 | BMI | BMI | Unadjusted 0.03 (95% CI -0.01, 0.07) |
| Zalbahar *et al.* 2015[14] | 1 | 145 | Malaysia | USM Pregnancy Cohort^c^ | 8 | BMI | BMI z-score | Adjusted 0.04 (95% CI 0.00, 0.07) |
| Tan *et al.* 2015[15] | 8 to 18 | 68 | USA | Prenatal Exposures and Preeclampsia Prevention | 6 | BMI | BMI z-score | Adjusted 0.19, p=0.02 |
| Additional studies identified in the updated searches March 2019 | | | | | | | | |
| Fujita et al. 2018[16] | 3 | 480 | Japan | Population: Fukurio, Japan | 7 | BMI | BMI z-score | Male: Adjusted 0.075, p=0.277  Female: Adjusted 0.22, p=0.002 |
| Fujita et al. 2018[16] | 13 | 480 | Japan | Population: Fukurio, Japan | 7 | BMI | BMI z-score | Male: Adjusted 0.19, p=0.005  Female: Adjusted 0.508, p<0.001 |

Abbreviations: BMI, body mass index; CI, confidence interval; SE, standard error.

Footnote:

^a^Sample size included in the analysis reported in the table rather than sample size of the entire cohort/study population.

^b^Summary of associations between continuous maternal BMI and child BMI or z-score: A further 16 studies reported associations between continuous maternal and child BMI or BMI z-scores for ages 1-18; seven studies reported associations between maternal BMI and child z-score[4, 7, 9, 11, 14-16], five between maternal and child BMI[1, 5, 6, 12, 13], four between maternal and child z-score[1, 3, 8, 10] and one maternal z-score and child BMI[2]. All showed a significantly increased association with the exception of one study[13]. Fujita *et al.*[16] also reported non-significant association for males aged 3, but significant associations at age 13 and among females aged 3 and 13.

^c^Abbreviated cohort names, for full cohort names see table S4.

**References:**

1. Davey Smith G, Steer C, Leary S, Ness A. Is there an intrauterine influence on obesity? Evidence from parent child associations in the Avon Longitudinal Study of Parents and Children (ALSPAC). Archives of disease in childhood. 2007;92(10):876-80. Epub 2007/06/28.

2. Durmus B, Arends LR, Ay L, Hokken-Koelega AC, Raat H, Hofman A, et al. Parental anthropometrics, early growth and the risk of overweight in pre-school children: the Generation R Study. Pediatric Obesity. 2012;8(5):339-50.

3. Gaillard R, Steegers EA, Duijts L, Felix JF, Hofman A, Franco OH, et al. Childhood cardiometabolic outcomes of maternal obesity during pregnancy: the Generation R Study. Hypertension. 2014;63(4):683-91.

4. Ehrenthal DB, Maiden K, Rao A, West DW, Gidding SS, Bartoshesky L, et al. Independent relation of maternal prenatal factors to early childhood obesity in the offspring. Obstet Gynecol. 2013;121(1):115-21.

5. Gademan MG, Vermeulen M, Oostvogels AJ, Roseboom TJ, Visscher TL, van Eijsden M, et al. Maternal prepregancy BMI and lipid profile during early pregnancy are independently associated with offspring's body composition at age 5-6 years: the ABCD study. PLoS ONE. 2014;9(4):e94594.

6. Mesman I, Roseboom TJ, Bonsel GJ, Gemke RJ, van der Wal MF, Vrijkotte TGM. Maternal pre-pregnancy body mass index explains infant’s weight and BMI at 14 months: results from a multi-ethnic birth cohort study. Archives of disease in childhood. 2009;94(8):587-95.

7. Jacota M, Forhan A, Saldanha-Gomes C, Charles MA, Heude B, for the EMCCSG. Maternal weight prior and during pregnancy and offspring's BMI and adiposity at 5–6 years in the EDEN mother–child cohort. Pediatric Obesity. 2016.

8. Knight B, Shields BM, Hill A, Powell RJ, Wright D, Hattersley AT. The impact of maternal glycemia and obesity on early postnatal growth in a nondiabetic Caucasian population. Diabetes Care. 2007;30(4):777-83.

9. Li A, Teo KK, Morrison KM, McDonald SD, Atkinson SA, Anand SS, et al. A genetic link between prepregnancy body mass index, postpartum weight retention, and offspring weight in early childhood. Obesity. 2017;25(1):236-43.

10. Morgen C, Angquist L, Baker J, Andersen A, Michaelsen K, SoRensen T. Prenatal risk factors infuencing childhood BMI and overweight independent of birth weight and infancy BMI - A path analysis within the Danish national birth cohort. Obesity Facts. 2017;10:21-2.

11. Sorensen TIA, Ajslev TA, Angquist L, Morgen CS, Ciuchi IG, Smith GD. Comparison of associations of maternal peri-pregnancy and paternal anthropometrics with child anthropometrics from birth through age 7 y assessed in the Danish National Birth Cohort. Am J Clin Nutr. 2016;104(2):389-96.

12. Terry MB, Wei Y, Esserman D, McKeague IW, Susser E. Pre- and postnatal determinants of childhood body size: cohort and sibling analyses. Journal of Developmental Origins of Health and Disease. 2011;2(2):99-111.

13. Wen LM, Baur LA, Rissel C, Xu H, Simpson JM. Correlates of body mass index and overweight and obesity of children aged 2 years: findings from the healthy beginnings trial. Obesity (Silver Spring). 2014;22(7):1723-30.

14. Zalbahar N, Jan Mohamed HJB, Loy SL, Najman J, McIntyre HD, Mamun A. Association of parental body mass index before pregnancy on infant growth and body composition: Evidence from a pregnancy cohort study in Malaysia. Obesity Research and Clinical Practice. 2016;10:S35-S47.

15. Tan HC, Roberts J, Catov J, Krishnamurthy R, Shypailo R, Bacha F. Mother's pre-pregnancy BMI is an important determinant of adverse cardiometabolic risk in childhood. Pediatric Diabetes. 2015;16(6):419-26.

16. Fujita Y, Kouda K, Nakamura H, Iki M. Relationship Between Maternal Pre-pregnancy Weight and Offspring Weight Strengthens as Children Develop: A Population-Based Retrospective Cohort Study. J Epidemiol. 2018:JE20170137.
