# Supplementary material for: The association between maternal body mass index and child obesity: A systematic review and meta-analysis
Source: PLoS Med. 2019 Jun 11;16(6):e1002817. doi: 10.1371/journal.pmed.1002817 (PMC6559702; doi:10.1371/journal.pmed.1002817)
Supplement: S14 Table — (DOCX) [file pmed.1002817.s024.docx]

# S14 Table: Maternal BMI and child overweight/obesity (BMI ≥85^th^ percentile) sensitivity analysis^a^

|  | **I^2^** % (95% CI) | **Linear analyses**  OR (95% CI) | **Nonlinear Analyses: Maternal BMI Midpoint (kg/m^2^)^b^**  OR (95% CI) | | | |
| --- | --- | --- | --- | --- | --- | --- |
|  |  | **Per 5 unit increase in maternal BMI** | **17.5** | **22.5** | **27.5** | **35.0** |
| Berkowitz *et al.* 2005[1] | 92.7 (85.8, 97.0) | 1.54 (1.42,1.68) | N/A | N/A | N/A | N/A |
| Catalano *et al.* 2009[2] | 93.1 (87.2, 97.7) | 1.55 (1.42,1.70) | 0.5 (0.42,0.6) | 1 | 1.64 (1.45,1.85) | 2.68 (2.07,3.48) |
| Daraki *et al.* 2015[3] | 93.0 (86.6, 97.6) | 1.57 (1.43,1.71) | N/A | N/A | N/A | N/A |
| Gademan *et al.* 2014[4] | 92.4 (85.7, 97.5) | 1.53 (1.41,1.67) | 0.52 (0.45,0.62) | 1 | 1.6 (1.43,1.79) | 2.57 (2.01,3.29) |
| Gaillard *et al.* 2014[5] | 92.0 (84.7, 97.4) | 1.53 (1.41,1.67) | 0.51 (0.43,0.61) | 1 | 1.61 (1.43,1.81) | 2.56 (1.99,3.28) |
| Guo *et al.* 2015[6] | 91.1 (83.0, 96.9) | 1.57 (1.44,1.71) | 0.48 (0.42,0.56) | 1 | 1.68 (1.5,1.89) | 2.78 (2.14,3.62) |
| Hinkle *et al.* 2012[7] | 92.5 (85.7, 97.4) | 1.57 (1.43,1.71) | 0.5 (0.42,0.6) | 1 | 1.65 (1.46,1.87) | 2.75 (2.1,3.6) |
| Kubo *et al.* 2016[8] | 93.2 (87.1, 97.6) | 1.56 (1.43,1.70) | 0.51 (0.43,0.6) | 1 | 1.64 (1.45,1.85) | 2.7 (2.07,3.52) |
| Laitinen *et al.* 2012[9] | 92.5 (85.7, 97.5) | 1.54 (1.41,1.68) | 0.51 (0.43,0.61) | 1 | 1.61 (1.43,1.82) | 2.59 (2,3.35) |
| Li *et al.* 2013[10] | 88.9 (79.2, 96.1) | 1.57 (1.44,1.72) | 0.49 (0.41,0.6) | 1 | 1.68 (1.48,1.89) | 2.86 (2.23,3.66) |
| Lindberg *et al.* 2012[11] | 92.5 (85.7, 97.4) | 1.57 (1.44,1.71) | 0.49 (0.42,0.59) | 1 | 1.67 (1.48,1.88) | 2.79 (2.15,3.61) |
| Margerison-Zilko *et al.* 2012[12] | 93.0 (86.6, 97.6) | 1.56 (1.43,1.71) | 0.5 (0.42,0.6) | 1 | 1.65 (1.46,1.86) | 2.74 (2.1,3.59) |
| Massion *et al.* 2016[13] | 91.4 (83.7, 97.2) | 1.53 (1.41,1.67) | 0.51 (0.42,0.61) | 1 | 1.61 (1.43,1.82) | 2.53 (1.98,3.22) |
| O'Callaghan *et al.* 1997[14] | 93.0 (86.7, 97.6) | 1.55 (1.42,1.70) | 0.51 (0.43,0.61) | 1 | 1.63 (1.44,1.84) | 2.68 (2.05,3.52) |
| Olson *et al.* 2010[15] | 93.1 (87.0, 97.6) | 1.56 (1.43,1.71) | 0.5 (0.42,0.59) | 1 | 1.65 (1.46,1.87) | 2.72 (2.09,3.55) |
| Rios-Castillo *et al.* 2007[16] | 93.1 (87.0, 97.6) | 1.56 (1.43,1.71) | 0.5 (0.43,0.6) | 1 | 1.64 (1.46,1.84) | 2.69 (2.1,3.46) |
| Risvas *et al.* 2012[17] | 91.7 (84.3, 97.3) | 1.53 (1.41,1.66) | N/A | N/A | N/A | N/A |
| Robinson *et al.* 2014[18] | 92.2 (85.0, 97.3) | 1.57 (1.44,1.72) | 0.51 (0.44,0.6) | 1 | 1.69 (1.5,1.91) | 2.81 (2.17,3.64) |
| Rooney *et al.* 2011[19] | 92.5 (85.7, 97.4) | 1.54 (1.41,1.67) | 0.5 (0.43,0.6) | 1 | 1.64 (1.46,1.84) | 2.69 (2.1,3.46) |
| Tan *et al.* 2015[20] | 92.8 (86.6, 97.5) | 1.54 (1.42,1.68) | 0.5 (0.43,0.6) | 1 | 1.64 (1.46,1.84) | 2.69 (2.1,3.46) |
| Wen *et al.* 2014[21] | 92.7 (86.0, 97.4) | 1.57 (1.44,1.71) | N/A | N/A | N/A | N/A |
| Zhang *et al.* 2013[22] | 93.0 (87.0, 97.6) | 1.55 (1.42,1.69) | N/A | N/A | N/A | N/A |

Abbreviations: OR, odds ratio; CI, confidence interval; BMI, body mass index; N/A, not applicable as study was excluded from nonlinear analysis for reporting only 2 BMI categories.

Footnote:

^a^Sensitivity analyses were performed by excluding one study at a time from the meta-analysis to identify the effect of any one individual study.

^b^The summary OR represent BMI mid-points of categories of underweight (17.5kg/m^2^), recommended BMI (22.5kg/m^2^), overweight 27.5kg/m^2^) and obesity (35.0kg/m^2^).

**References:**

1. Berkowitz RI, Stallings VA, Maislin G, Stunkard AJ. Growth of children at high risk of obesity during the first 6 y of life: implications for prevention. Am J Clin Nutr. 2005;81(1):140-6.

2. Catalano PM, Farrell K, Thomas A, Huston-Presley L, Mencin P, de Mouzon SH, et al. Perinatal risk factors for childhood obesity and metabolic dysregulation. Am J Clin Nutr. 2009;90(5):1303-13.

3. Daraki V, Georgiou V, Papavasiliou S, Chalkiadaki G, Karahaliou M, Koinaki S, et al. Metabolic profile in early pregnancy is associated with offspring adiposity at 4 years of age: the Rhea pregnancy cohort Crete, Greece. PLoS ONE. 2015;10(5):e0126327.

4. Gademan MG, Vermeulen M, Oostvogels AJ, Roseboom TJ, Visscher TL, van Eijsden M, et al. Maternal prepregancy BMI and lipid profile during early pregnancy are independently associated with offspring's body composition at age 5-6 years: the ABCD study. PLoS ONE. 2014;9(4):e94594.

5. Gaillard R, Steegers EA, Duijts L, Felix JF, Hofman A, Franco OH, et al. Childhood cardiometabolic outcomes of maternal obesity during pregnancy: the Generation R Study. Hypertension. 2014;63(4):683-91.

6. Guo L, Liu J, Ye R, Liu J, Zhuang Z, Ren A. Gestational Weight Gain and Overweight in Children Aged 3-6 Years. J Epidemiol. 2015;25(8):536-43.

7. Hinkle SN, Sharma AJ, Swan DW, Schieve LA, Ramakrishnan U, Stein AD. Excess gestational weight gain is associated with child adiposity among mothers with normal and overweight prepregnancy weight status. J Nutr. 2012;142(10):1851-8.

8. Kubo A, Ferrara A, Laurent CA, Windham GC, Greenspan LC, Deardorff J, et al. Associations Between Maternal Pregravid Obesity and Gestational Diabetes and the Timing of Pubarche in Daughters. Am J Epidemiol. 2016;184(1):7-14.

9. Laitinen J, Jaaskelainen A, Hartikainen AL, Sovio U, Vaarasmaki M, Pouta A, et al. Maternal weight gain during the first half of pregnancy and offspring obesity at 16 years: a prospective cohort study. Bjog. 2012;119(6):716-23.

10. Li N, Liu E, Guo J, Pan L, Li B, Wang P, et al. Maternal prepregnancy body mass index and gestational weight gain on offspring overweight in early infancy. PLoS ONE. 2013;8(10):e77809.

11. Lindberg SM, Adams AK, Prince RJ. Early predictors of obesity and cardiovascular risk among American Indian children. Matern Child Health J. 2012;16(9):1879-86.

12. Margerison-Zilko CE, Shrimali BP, Eskenazi B, Lahiff M, Lindquist AR, Abrams BF. Trimester of maternal gestational weight gain and offspring body weight at birth and age five. Matern Child Health J. 2012;16(6):1215-23.

13. Massion S, Wickham S, Pearce A, Barr B, Law C, Taylor-Robinson D. Exploring the impact of early life factors on inequalities in risk of overweight in UK children: findings from the UK Millennium Cohort Study. Archives of disease in childhood. 2016. Epub 2016/05/11.

14. O'Callaghan MJ, Williams GM, Andersen MJ, Bor W, Najman JM. Prediction of obesity in children at 5 years: a cohort study. J Paediatr Child Health. 1997;33(4):311-6.

15. Olson CM, Demment MM, Carling SJ, Strawderman MS. Associations Between Mothers' and Their Children's Weights at 4 Years of Age. Childhood obesity. 2010;6(4):201-7.

16. Rios-Castillo I, Cerezo S, Corvalan C, Martinez M, Kain J. Risk factors during the prenatal period and the first year of life associated with overweight in 7-year-old low-income Chilean children. Maternal and Child Nutrition. 2015;11(4):595-605.

17. Risvas G, Papaioannou I, Panagiotakos DB, Farajian P, Bountziouka V, Zampelas A. Perinatal and family factors associated with preadolescence overweight/obesity in Greece: the GRECO study. J Epidemiol Glob Health. 2012;2(3):145-53.

18. Robinson CA, Cohen AK, Rehkopf DH, Deardorff J, Ritchie L, Jayaweera RT, et al. Pregnancy and post-delivery maternal weight changes and overweight in preschool children. Prev Med. 2014;60:77-82.

19. Rooney BL, Mathiason MA, Schauberger CW. Predictors of obesity in childhood, adolescence, and adulthood in a birth cohort. Matern Child Health J. 2011;15(8):1166-75.

20. Tan HC, Roberts J, Catov J, Krishnamurthy R, Shypailo R, Bacha F. Mother's pre-pregnancy BMI is an important determinant of adverse cardiometabolic risk in childhood. Pediatric Diabetes. 2015;16(6):419-26.

21. Wen LM, Baur LA, Rissel C, Xu H, Simpson JM. Correlates of body mass index and overweight and obesity of children aged 2 years: findings from the healthy beginnings trial. Obesity (Silver Spring). 2014;22(7):1723-30.

22. Zhang J, Himes JH, Guo Y, Jiang J, Yang L, Lu Q, et al. Birth weight, growth and feeding pattern in early infancy predict overweight/obesity status at two years of age: a birth cohort study of Chinese infants. PLoS ONE. 2013;8(6):e64542.
