# Supplementary material for: The association between maternal body mass index and child obesity: A systematic review and meta-analysis
Source: PLoS Med. 2019 Jun 11;16(6):e1002817. doi: 10.1371/journal.pmed.1002817 (PMC6559702; doi:10.1371/journal.pmed.1002817)
Supplement: S15 Table — (DOCX) [file pmed.1002817.s025.docx]

# S15 Table: Maternal BMI and child overweight (BMI 85^th^ to 95^th^ percentile) sensitivity analysis^a^

|  | **I^2^ % (95% CI)** | **Linear analyses**  OR (95% CI) | **Nonlinear Analyses: Maternal BMI Midpoint (kg/m^2^)^b^**  OR (95% CI) | | | |
| --- | --- | --- | --- | --- | --- | --- |
|  |  | **Per 5 unit increase in maternal BMI** | **17.5** | **22.5** | **27.5** | **35.0** |
| Berkowitz *et al.* 2005[1] | 93.8 (84.1, 98.2) | 1.31 (1.15,1.48) | N/A | N/A | N/A | N/A |
| Catalano *et al.* 2009[2] | 94.0 (84.8, 98.7) | 1.32 (1.17,1.50) | 0.64 (0.52,0.79) | 1 | 1.42 (1.19,1.7) | 1.82 (1.24,2.67) |
| Gaillard *et al.* 2014[3] | 91.4 (76.1, 98.2) | 1.27 (1.13,1.43) | 0.69 (0.58,0.82) | 1 | 1.34 (1.15,1.56) | 1.63 (1.13,2.35) |
| Hinkle *et al.* 2012[4] | 93.8 (83.0, 98.6) | 1.32 (1.15,1.52) | 0.65 (0.52,0.81) | 1 | 1.41 (1.16,1.71) | 1.81 (1.19,2.75) |
| Kubo *et al.* 2016[5] | 94.2 (84.9, 98.7) | 1.32 (1.16,1.51) | 0.64 (0.52,0.8) | 1 | 1.41 (1.17,1.7) | 1.8 (1.2,2.69) |
| Li *et al.* 2013[6] | 89.4 (72.0, 97.6) | 1.34 (1.17,1.53) | 0.59 (0.48,0.72) | 1 | 1.49 (1.25,1.77) | 1.83 (1.2,2.78) |
| Lindberg *et al.* 2012[7] | 93.5 (82.6, 98.6) | 1.34 (1.18,1.52) | 0.62 (0.51,0.77) | 1 | 1.45 (1.21,1.73) | 1.88 (1.27,2.79) |
| Massion *et al.* 2016[8] | 89.2 (69.6, 97.8) | 1.27 (1.13,1.42) | 0.66 (0.52,0.83) | 1 | 1.36 (1.14,1.63) | 1.58 (1.16,2.16) |
| O’Callaghan *et al.* 1997[9] | 94.3 (84.4, 98.7) | 1.32 (1.15,1.51) | 0.64 (0.52,0.81) | 1 | 1.41 (1.16,1.71) | 1.81 (1.19,2.74) |
| Olson *et al.* 2010[10] | 92.7 (80.4, 98.3) | 1.35 (1.20,1.52) | 0.61 (0.5,0.75) | 1 | 1.47 (1.24,1.74) | 1.96 (1.36,2.81) |

Abbreviations: OR, odds ratio; CI, confidence interval; BMI, body mass index; N/A, not applicable as study was excluded from nonlinear analysis for reporting only 2 BMI categories.

Footnote:

^a^Sensitivity analyses were performed by excluding one study at a time from the meta-analysis to identify the effect of any one individual study.

^b^The summary OR represent BMI mid-points of categories of underweight (17.5kg/m^2^), recommended BMI (22.5kg/m^2^), overweight 27.5kg/m^2^) and obesity (35.0kg/m^2^).

**References:**

1. Berkowitz RI, Stallings VA, Maislin G, Stunkard AJ. Growth of children at high risk of obesity during the first 6 y of life: implications for prevention. Am J Clin Nutr. 2005;81(1):140-6.

2. Catalano PM, Farrell K, Thomas A, Huston-Presley L, Mencin P, de Mouzon SH, et al. Perinatal risk factors for childhood obesity and metabolic dysregulation. Am J Clin Nutr. 2009;90(5):1303-13.

3. Gaillard R, Steegers EA, Duijts L, Felix JF, Hofman A, Franco OH, et al. Childhood cardiometabolic outcomes of maternal obesity during pregnancy: the Generation R Study. Hypertension. 2014;63(4):683-91.

4. Hinkle SN, Sharma AJ, Swan DW, Schieve LA, Ramakrishnan U, Stein AD. Excess gestational weight gain is associated with child adiposity among mothers with normal and overweight prepregnancy weight status. J Nutr. 2012;142(10):1851-8.

5. Kubo A, Ferrara A, Laurent CA, Windham GC, Greenspan LC, Deardorff J, et al. Associations Between Maternal Pregravid Obesity and Gestational Diabetes and the Timing of Pubarche in Daughters. Am J Epidemiol. 2016;184(1):7-14.

6. Li N, Liu E, Guo J, Pan L, Li B, Wang P, et al. Maternal prepregnancy body mass index and gestational weight gain on offspring overweight in early infancy. PLoS ONE. 2013;8(10):e77809.

7. Lindberg SM, Adams AK, Prince RJ. Early predictors of obesity and cardiovascular risk among American Indian children. Matern Child Health J. 2012;16(9):1879-86.

8. Massion S, Wickham S, Pearce A, Barr B, Law C, Taylor-Robinson D. Exploring the impact of early life factors on inequalities in risk of overweight in UK children: findings from the UK Millennium Cohort Study. Archives of disease in childhood. 2016. Epub 2016/05/11.

9. O'Callaghan MJ, Williams GM, Andersen MJ, Bor W, Najman JM. Prediction of obesity in children at 5 years: a cohort study. J Paediatr Child Health. 1997;33(4):311-6.

10. Olson CM, Demment MM, Carling SJ, Strawderman MS. Associations Between Mothers' and Their Children's Weights at 4 Years of Age. Childhood obesity. 2010;6(4):201-7.
