# Supplementary material for: The association between maternal body mass index and child obesity: A systematic review and meta-analysis
Source: PLoS Med. 2019 Jun 11;16(6):e1002817. doi: 10.1371/journal.pmed.1002817 (PMC6559702; doi:10.1371/journal.pmed.1002817)
Supplement: S16 Table — (DOCX) [file pmed.1002817.s026.docx]

# S16 Table: Maternal BMI and child obesity (BMI ≥95^th^ percentile) sensitivity analysis^a^

|  | **I^2^ %**  **(95% CI)** | **Linear analyses**  OR (95% CI) | **Nonlinear Analyses: Maternal BMI Midpoint (kg/m^2^)^b^**  OR (95% CI) | | | |
| --- | --- | --- | --- | --- | --- | --- |
|  |  | **Per 5 unit increase in maternal BMI** | **17.5** | **22.5** | **27.5** | **35.0** |
| Berkowitz *et al.* 2005[1] | 92.7 (85.9, 97.1) | 1.69 (1.54,1.87) | N/A | N/A | N/A | N/A |
| Catalano *et al.* 2009[2] | 92.9 (86.6, 97.6) | 1.70 (1.54,1.87) | 0.47 (0.39,0.58) | 1 | 1.88 (1.61,2.2) | 3.6 (2.62,4.96) |
| Diesel *et al.* 2014[3] | 92.6 (85.8, 97.4) | 1.72 (1.56,1.90) | 0.47 (0.39,0.58) | 1 | 1.9 (1.63,2.23) | 3.76 (2.74,5.17) |
| Gaillard *et al.* 2014[4] | 90.4 (81.3, 96.9) | 1.66 (1.52,1.82) | 0.48 (0.39,0.58) | 1 | 1.86 (1.59,2.17) | 3.37 (2.52,4.52) |
| Gillman *et al.* 2008[5] | 93.0 (86.5, 97.5) | 1.70 (1.54,1.88) | N/A | N/A | N/A | N/A |
| Hinkle *et al.* 2012[6] | 92.5 (85.5, 97.4) | 1.71 (1.55,1.89) | 0.48 (0.39,0.59) | 1 | 1.88 (1.6,2.21) | 3.71 (2.67,5.14) |
| Janjua *et al.* 2012[7] | 93.0 (86.5, 97.5) | 1.71 (1.55,1.89) | 0.48 (0.39,0.58) | 1 | 1.88 (1.61,2.2) | 3.67 (2.65,5.07) |
| Kubo *et al.* 2016[8] | 93.0 (86.6, 97.6) | 1.70 (1.54,1.88) | 0.48 (0.39,0.58) | 1 | 1.87 (1.6,2.19) | 3.61 (2.62,4.99) |
| Li *et al.* 2013[9] | 88.6 (78.3, 96.1) | 1.73 (1.57,1.91) | 0.47 (0.38,0.58) | 1 | 1.91 (1.63,2.25) | 3.87 (2.85,5.25) |
| Lindberg *et al.* 2012[10] | 92.9 (86.3, 97.5) | 1.71 (1.55,1.89) | 0.46 (0.38,0.56) | 1 | 1.92 (1.64,2.24) | 3.67 (2.65,5.09) |
| Massion *et al.* 2016[11] | 91.7 (83.8, 97.1) | 1.68 (1.52,1.85) | 0.48 (0.39,0.59) | 1 | 1.85 (1.58,2.17) | 3.47 (2.54,4.75) |
| O’Callaghan *et al.* 1997[12] | 92.9 (86.4, 97.5) | 1.70 (1.53,1.88) | 0.48 (0.4,0.59) | 1 | 1.86 (1.59,2.18) | 3.61 (2.61,5) |
| Oken *et al.* 2008[13] | 91.9 (84.2, 97.2) | 1.68 (1.52,1.85) | 0.49 (0.4,0.6) | 1 | 1.84 (1.57,2.15) | 3.52 (2.56,4.85) |
| Olson *et al.* 2010[14] | 92.6 (85.8, 97.4) | 1.68 (1.53,1.86) | 0.48 (0.39,0.58) | 1 | 1.86 (1.6,2.18) | 3.51 (2.56,4.81) |
| Rath *et al.* 2016[15] | 91.6 (83.6, 97.0) | 1.67 (1.52,1.83) | 0.5 (0.42,0.6) | 1 | 1.82 (1.58,2.09) | 3.42 (2.53,4.63) |
| Salsberry *et al.* 2005[16] | 92.7 (85.9, 97.4) | 1.71 (1.54,1.89) | 0.45 (0.39,0.51) | 1 | 1.97 (1.75,2.22) | 3.92 (2.92,5.27) |
| Whitaker 2004[17] | 91.2 (83.0, 96.9) | 1.72 (1.56,1.90) | 0.47 (0.38,0.58) | 1 | 1.91 (1.62,2.24) | 3.76 (2.72,5.19) |
| Wojcicki *et al.* 2015[18] | 92.1 (84.7, 97.3) | 1.73 (1.57,1.90) | 0.46 (0.38,0.54) | 1 | 1.94 (1.68,2.25) | 3.77 (2.74,5.21) |
| Wrotniak *et al.* 2008[19] | 92.3 (85.0, 97.3) | 1.71 (1.55,1.90) | 0.48 (0.39,0.59) | 1 | 1.88 (1.6,2.21) | 3.72 (2.69,5.16) |
| Zhang *et al.* 2013[20] | 92.4 (85.1, 96.9) | 1.72 (1.56,1.89) | N/A | N/A | N/A | N/A |

Abbreviations: OR, odds ratio; CI, confidence interval; BMI, body mass index; N/A, not applicable as study was excluded from nonlinear analysis for reporting only 2 BMI categories.

Footnote:

^a^Sensitivity analyses were performed by excluding one study at a time from the meta-analysis to identify the effect of any one individual study.

^b^The summary OR represent BMI mid-points of categories of underweight (17.5kg/m^2^), recommended BMI (22.5kg/m^2^), overweight 27.5kg/m^2^) and obesity (35.0kg/m^2^).

**References:**

1. Berkowitz RI, Stallings VA, Maislin G, Stunkard AJ. Growth of children at high risk of obesity during the first 6 y of life: implications for prevention. Am J Clin Nutr. 2005;81(1):140-6.

2. Catalano PM, Farrell K, Thomas A, Huston-Presley L, Mencin P, de Mouzon SH, et al. Perinatal risk factors for childhood obesity and metabolic dysregulation. Am J Clin Nutr. 2009;90(5):1303-13.

3. Diesel JC, Eckhardt CL, Day NL, Brooks MM, Arslanian SA, Bodnar LM. Is gestational weight gain associated with offspring obesity at 36 months? Pediatr Obes. 2014;10(4):305-10.

4. Gaillard R, Steegers EA, Duijts L, Felix JF, Hofman A, Franco OH, et al. Childhood cardiometabolic outcomes of maternal obesity during pregnancy: the Generation R Study. Hypertension. 2014;63(4):683-91.

5. Gillman MW, Rifas-Shiman SL, Kleinman K, Oken E, Rich-Edwards JW, Taveras EM. Developmental origins of childhood overweight: potential public health impact. Obesity. 2008;16(7):1651-6.

6. Hinkle SN, Sharma AJ, Swan DW, Schieve LA, Ramakrishnan U, Stein AD. Excess gestational weight gain is associated with child adiposity among mothers with normal and overweight prepregnancy weight status. J Nutr. 2012;142(10):1851-8.

7. Janjua NZ, Mahmood B, Islam MA, Goldenberg RL. Maternal and early childhood risk factors for overweight and obesity among low-income predominantly black children at age five years: A prospective cohort study. Journal of Obesity. 2012;457173.

8. Kubo A, Ferrara A, Laurent CA, Windham GC, Greenspan LC, Deardorff J, et al. Associations Between Maternal Pregravid Obesity and Gestational Diabetes and the Timing of Pubarche in Daughters. Am J Epidemiol. 2016;184(1):7-14.

9. Li N, Liu E, Guo J, Pan L, Li B, Wang P, et al. Maternal prepregnancy body mass index and gestational weight gain on offspring overweight in early infancy. PLoS ONE. 2013;8(10):e77809.

10. Lindberg SM, Adams AK, Prince RJ. Early predictors of obesity and cardiovascular risk among American Indian children. Matern Child Health J. 2012;16(9):1879-86.

11. Massion S, Wickham S, Pearce A, Barr B, Law C, Taylor-Robinson D. Exploring the impact of early life factors on inequalities in risk of overweight in UK children: findings from the UK Millennium Cohort Study. Archives of disease in childhood. 2016. Epub 2016/05/11.

12. O'Callaghan MJ, Williams GM, Andersen MJ, Bor W, Najman JM. Prediction of obesity in children at 5 years: a cohort study. J Paediatr Child Health. 1997;33(4):311-6.

13. Oken E, Rifas-Shiman SL, Field AE, Frazier AL, Gillman MW. Maternal gestational weight gain and offspring weight in adolescence. Obstet Gynecol. 2008;112(5):999-1006.

14. Olson CM, Demment MM, Carling SJ, Strawderman MS. Associations Between Mothers' and Their Children's Weights at 4 Years of Age. Childhood obesity. 2010;6(4):201-7.

15. Rath SR, Marsh JA, Newnham JP, Zhu K, Atkinson HC, Mountain J, et al. Parental pre-pregnancy BMI is a dominant early-life risk factor influencing BMI of offspring in adulthood. Obesity Science and Practice. 2016;2(1):48-57.

16. Salsberry PJ, Reagan PB. Dynamics of early childhood overweight. Pediatrics. 2005;116(6):1329-38.

17. Whitaker RC. Predicting preschooler obesity at birth: the role of maternal obesity in early pregnancy. Pediatrics. 2004;114(1):e29-36.

18. Wojcicki JM, Young MB, Perham-Hester KA, de Schweinitz P, Gessner BD. Risk factors for obesity at age 3 in Alaskan children, including the role of beverage consumption: results from Alaska PRAMS 2005-2006 and its three-year follow-up survey, CUBS, 2008-2009. PLoS ONE. 2015;10(3):e0118711.

19. Wrotniak BH, Shults J, Butts S, Stettler N. Gestational weight gain and risk of overweight in the offspring at age 7 y in a multicenter, multiethnic cohort study. Am J Clin Nutr. 2008;87(6):1818-24.

20. Zhang J, Himes JH, Guo Y, Jiang J, Yang L, Lu Q, et al. Birth weight, growth and feeding pattern in early infancy predict overweight/obesity status at two years of age: a birth cohort study of Chinese infants. PLoS ONE. 2013;8(6):e64542.
