# Supplementary material for: The association between maternal body mass index and child obesity: A systematic review and meta-analysis
Source: PLoS Med. 2019 Jun 11;16(6):e1002817. doi: 10.1371/journal.pmed.1002817 (PMC6559702; doi:10.1371/journal.pmed.1002817)
Supplement: S17 Table — (DOCX) [file pmed.1002817.s027.docx]

# S17 Table: Maternal BMI and continuous child BMI and BMI z-score outcomes (mean differences) sensitivity analysis^a^

|  | **I^2^** % (95% CI) | **Linear analyses**  SMD (95% CI) | **Nonlinear Analyses: Maternal BMI Midpoint (kg/m^2^)^b^**  SMD (95% CI) | | | |
| --- | --- | --- | --- | --- | --- | --- |
|  |  | **Per 5 unit increase in maternal BMI** | **17.5** | **22.5** | **27.5** | **35.0** |
| Andres *et al.* 2015[1] | 99.9 (99.8, 99.9) | 0.39 (0.08,0.70) | -0.52 (-0.69,-0.35) | 0 | 0.47 (0.31,0.62) | 1.02 (0.61,1.44) |
| Berkowitz *et al.* 2005[2] | 99.9 (99.8, 99.95) | 0.48 (0.13,0.83) | N/A | N/A | N/A | N/A |
| Daraki *et al.* 2015[3] | 99.6 (99.3, 99.8) | 0.33 (0.15,0.51) | N/A | N/A | N/A | N/A |
| Deierlein *et al.* 2011[4] | 99.9 (99.8 99.95) | 0.48 (0.13,0.83) | -0.52 (-0.68,-0.36) | 0 | 0.47 (0.31,0.62) | 1.05 (0.64,1.46) |
| Eisenman *et al.* 2010[5] | 99.9 (99.8, 99.95) | 0.48 (0.13,0.83) | N/A | N/A | N/A | N/A |
| Fleten *et al.* 2012[6] | 99.9 (99.8, 99.9) | 0.49 (0.14,0.84) | N/A | N/A | N/A | N/A |
| Gademan *et al.* 2014[7] | 99.9 (99.8, 99.95) | 0.48 (0.13,0.83) | -0.48 (-0.63,-0.33) | 0 | 0.44 (0.29,0.59) | 1.02 (0.59,1.44) |
| Gaillard *et al.* 2014[8] | 99.9 (99.8, 99.9) | 0.48 (0.13,0.83) | N/A | N/A | N/A | N/A |
| Hinkle *et al.* 2012[9] | 99.9 (99.8, 99.9) | 0.49 (0.14,0.84) | -0.53 (-0.69,-0.37) | 0 | 0.47 (0.32,0.63) | 1.05 (0.65,1.45) |
| Jacota *et al.* 2016[10] | 99.9 (99.8, 99.95) | 0.48 (0.13,0.83) | -0.53 (-0.69,-0.36) | 0 | 0.47 (0.32,0.63) | 1.05 (0.66,1.45) |
| Kaar *et al.* 2014a [11] | 99.9 (99.8, 99.95) | 0.47 (0.13,0.82) |  |  |  |  |
| Kaar *et al.* 2014b [11] | 99.9 (99.8, 99.95) | 0.49 (0.15,0.83) | -0.52 (-0.67,-0.36) | 0 | 0.46 (0.32,0.61) | 1.03 (0.65,1.41) |
| Kaar *et al.* 2014c [11] | 99.9 (99.8, 99.95) | 0.49 (0.14,0.84) | -0.48 (-0.62,-0.34) | 0 | 0.42 (0.29,0.55) | 0.92 (0.58,1.27) |
| Kaar *et al.* 2014d [11] | 99.9 (99.8, 99.95) | 0.48 (0.13,0.83) | -0.51 (-0.67,-0.36) | 0 | 0.46 (0.31,0.61) | 1.02 (0.62,1.41) |
| Kaar *et al.* 2014e [11] | 99.9 (99.8, 99.95) | 0.49 (0.14,0.84) | -0.45 (-0.57,-0.33) | 0 | 0.39 (0.28,0.5) | 0.85 (0.56,1.14) |
| Kaar *et al.* 2014f [11] | 99.9 (99.8, 99.95) | 0.48 (0.13,0.83) | -0.48 (-0.63,-0.34) | 0 | 0.43 (0.29,0.57) | 0.95 (0.58,1.31) |
| Kaar *et al.* 2014g [11] | 99.9 (99.8, 99.95) | 0.48 (0.13,0.83) | -0.47 (-0.6,-0.34) | 0 | 0.42 (0.29,0.54) | 0.91 (0.58,1.25) |
| Kaar *et al.* 2014h [11] | 99.9 (99.8, 99.95) | 0.48 (0.13,0.83) | -0.5 (-0.65,-0.35) | 0 | 0.44 (0.31,0.58) | 0.98 (0.62,1.35) |
| Leng *et al.* 2015[12] | 99.9 (99.8, 99.95) | 0.49 (0.14,0.84) | -0.53 (-0.69,-0.36) | 0 | 0.47 (0.31,0.63) | 1.04 (0.63,1.45) |
| Li *et al.* 2013[13] | 99.8 (99.7, 99.9) | 0.49 (0.14,0.84) | -0.53 (-0.69,-0.38) | 0 | 0.47 (0.33,0.62) | 1.05 (0.66,1.44) |
| Makela *et al.* 2013[14] | 99.9 (99.8, 99.95) | 0.47 (0.12,0.83) | N/A | N/A | N/A | N/A |
| Margerison Zilko *et al.* 2012[15] | 99.9 (99.8, 99.95) | 0.48 (0.13,0.83) | -0.52 (-0.68,-0.35) | 0 | 0.46 (0.31,0.62) | 1.04 (0.63,1.46) |
| Mesman *et al.* 2009[16] | 99.9 (99.8, 99.9) | 0.49 (0.14,0.84) | -0.52 (-0.68,-0.35) | 0 | 0.47 (0.32,0.62) | 1.07 (0.68,1.46) |
| Toemen *et al.* 2016[17] | 99.9 (99.8, 99.9) | 0.48 (0.13,0.83) | -0.5 (-0.67,-0.34) | 0 | 0.42 (0.29,0.55) | 0.83 (0.54,1.11) |
| Zalbahar *et al.* 2015[18] | 99.9 (99.8, 99.95) | 0.48 (0.13,0.83) | N/A | N/A | N/A | N/A |

Abbreviations: SMD, standardised mean difference; CI, confidence interval; BMI, body mass index; N/A, not applicable as study was excluded from nonlinear analysis for reporting only 2 BMI categories.

Footnote:

^a^Sensitivity analyses were performed by excluding one study at a time from the meta-analysis to identify the effect of any one individual study.

^b^The summary OR represent BMI mid-points of categories of underweight (17.5kg/m^2^), recommended BMI (22.5kg/m^2^), overweight 27.5kg/m^2^) and obesity (35.0kg/m^2^).

**References:**

1. Andres A, Hull HR, Shankar K, Casey PH, Cleves MA, Badger TM. Longitudinal body composition of children born to mothers with normal weight, overweight, and obesity. Obesity. 2015;23(6):1252-8.

2. Berkowitz RI, Stallings VA, Maislin G, Stunkard AJ. Growth of children at high risk of obesity during the first 6 y of life: implications for prevention. Am J Clin Nutr. 2005;81(1):140-6.

3. Daraki V, Georgiou V, Papavasiliou S, Chalkiadaki G, Karahaliou M, Koinaki S, et al. Metabolic profile in early pregnancy is associated with offspring adiposity at 4 years of age: the Rhea pregnancy cohort Crete, Greece. PLoS ONE. 2015;10(5):e0126327.

4. Deierlein AL, Siega-Riz AM, Chantala K, Herring AH. The association between maternal glucose concentration and child BMI at age 3 years. Diabetes Care. 2011;34(2):480-4.

5. Eisenman JC, Sarzynski MA, Tucker J, Heelan KA. Maternal prepregnancy overweight and offspring fatness and blood pressure: role of physical activity. Pediatr Exerc Sci. 2010;22(3):369-78.

6. Fleten C, Nystad W, Stigum H, Skjaerven R, Lawlor DA, Davey Smith G, et al. Parent-offspring body mass index associations in the Norwegian Mother and Child Cohort Study: a family-based approach to studying the role of the intrauterine environment in childhood adiposity. Am J Epidemiol. 2012;176(2):83-92.

7. Gademan MG, Vermeulen M, Oostvogels AJ, Roseboom TJ, Visscher TL, van Eijsden M, et al. Maternal prepregancy BMI and lipid profile during early pregnancy are independently associated with offspring's body composition at age 5-6 years: the ABCD study. PLoS ONE. 2014;9(4):e94594.

8. Gaillard R, Steegers EA, Duijts L, Felix JF, Hofman A, Franco OH, et al. Childhood cardiometabolic outcomes of maternal obesity during pregnancy: the Generation R Study. Hypertension. 2014;63(4):683-91.

9. Hinkle SN, Sharma AJ, Swan DW, Schieve LA, Ramakrishnan U, Stein AD. Excess gestational weight gain is associated with child adiposity among mothers with normal and overweight prepregnancy weight status. J Nutr. 2012;142(10):1851-8.

10. Jacota M, Forhan A, Saldanha-Gomes C, Charles MA, Heude B, for the EMCCSG. Maternal weight prior and during pregnancy and offspring's BMI and adiposity at 5–6 years in the EDEN mother–child cohort. Pediatric Obesity. 2016.

11. Kaar JL, Crume T, Brinton JT, Bischoff KJ, McDuffie R, Dabelea D. Maternal obesity, gestational weight gain, and offspring adiposity: the exploring perinatal outcomes among children study. J Pediatr. 2014;165(3):509-15.

12. Leng J, Li W, Zhang S, Liu H, Wang L, Liu G, et al. GDM Women's Pre-Pregnancy Overweight/Obesity and Gestational Weight Gain on Offspring Overweight Status. PLoS ONE. 2015;10(6):e0129536.

13. Li N, Liu E, Guo J, Pan L, Li B, Wang P, et al. Maternal prepregnancy body mass index and gestational weight gain on offspring overweight in early infancy. PLoS ONE. 2013;8(10):e77809.

14. Makela J, Lagstrom H, Kaljonen A, Simell O, Niinikoski H. Hyperglycemia and lower diet quality in pregnant overweight women and increased infant size at birth and at 13 months of age--STEPS study. Early Human Development. 2013;89(6):439-44.

15. Margerison-Zilko CE, Shrimali BP, Eskenazi B, Lahiff M, Lindquist AR, Abrams BF. Trimester of maternal gestational weight gain and offspring body weight at birth and age five. Matern Child Health J. 2012;16(6):1215-23.

16. Mesman I, Roseboom TJ, Bonsel GJ, Gemke RJ, van der Wal MF, Vrijkotte TGM. Maternal pre-pregnancy body mass index explains infant’s weight and BMI at 14 months: results from a multi-ethnic birth cohort study. Archives of disease in childhood. 2009;94(8):587-95.

17. Toemen L, Gishti O, Van Osch-Gevers L, Steegers EAP, Helbing WA, Felix JF, et al. Maternal obesity, gestational weight gain and childhood cardiac outcomes: Role of childhood body mass index. Int J Obes. 2016;40(7):1070-8.

18. Zalbahar N, Jan Mohamed HJB, Loy SL, Najman J, McIntyre HD, Mamun A. Association of parental body mass index before pregnancy on infant growth and body composition: Evidence from a pregnancy cohort study in Malaysia. Obesity Research and Clinical Practice. 2016;10:S35-S47.
