# Supplementary material for: The association between maternal body mass index and child obesity: A systematic review and meta-analysis
Source: PLoS Med. 2019 Jun 11;16(6):e1002817. doi: 10.1371/journal.pmed.1002817 (PMC6559702; doi:10.1371/journal.pmed.1002817)
Supplement: S18 Table — (DOCX) [file pmed.1002817.s028.docx]

# S18 Table: Results of univariate meta-regression models evaluating the effect of potential sources of heterogeneity

|  | **Child Obesity (≥95^th^ percentile)** | | **Child overweight or obesity (≥85^th^ percentile)** | | **Child overweight (85^th^ to 95^th^ percentile)** | | **Continuous child BMI** | |
| --- | --- | --- | --- | --- | --- | --- | --- | --- |
| **Variable** | ***I^2^* % (95% CI)** | **p value^d^** | ***I^2^* % (95% CI)** | **p value^d^** | ***I^2^* % (95% CI)** | **p value^d^** | ***I^2^* % (95% CI)** | **p value^d^** |
| **All studies** | 92.3 (85.4, 97.2) | N/A | 92.5 (86.0, 97.4) | N/A | 92.7 (80.5, 98.3) | N/A | 99.9 (99.8,99.9) | N/A |
| **Study factors** | | | | | | | | |
| Quality | 91.0 (82.8, 96.8) | 0.895 | 91.3 (83.6, 96.9) | 0.812 | 89.4 (71.3, 97.7) | 0.489 | 99.9 (99.8, 99.9) | 0.308 |
| Year of Publication | 91.8 (84.2, 97.0) | 0.524 | 92.6 (85.7, 97.4) | 0.393 | 93.5 (81.7, 98.6) | 0.803 | 99.9 (99.8, 99.9) | 0.338 |
| Publication Decade | 91.4 (83.3, 96.8) | 0.349 | 92.6 | 0.482 | 93.1 (80.5, 98.5) | 0.531 | 99.9 (99.8, 99.9) | 0.642 |
| Year the study started recruitment | 91.5 (83.6, 96.8) | 0.705 | 93.1 (86.4, 97.3) | 0.924 | 90.1 (74.1, 97.9) | 0.875 | 99.9 (99.85, 99.96) | 0.669 |
| Decade the study started recruitment | 92.2 (84.5, 96.9) | 0.872 | 93.4 (87.6, 97.9) | 0.776 | 93.8 (82.6, 98.7) | 0.883 | 99.9 (99.8, 99.96) | 0.466 |
| Continent of study | **76.4 (52.0, 92.3)** ^a^ | **<0.0001**^a^ | **80.3 (66.1, 96.9)** ^a^ | **0.002**^a^ | **0.08 (0.00,96.9)** ^a^ | **<0.001**^a^ | 99.8 (99.7, 99.9) | 0.536 |
| Regional or national data | 92.5 (85.7, 97.5) | 0.845 | 92.8 (86.5, 97.6) | 0.928 | 89.6 (71.9,97.9) | 0.261 | 99.9 (99.8, 99.9) | 0.615 |
| Prospective or retrospective | 87.9 (76.8, 96.6) | 0.056 | 90.3 (82.0, 96.8) | 0.462 | 85.9 (62.4,97.3) | 0.181 | 99.8 (99.7, 99.9) | 0.565 |
| Sample size | 90.0 (80.9,96.4) | 0.407 | 91.4 (83.6, 97.0) | 0.237 | 89.6 (72.4, 97.7) | 0.971 | 99.9 (99.8, 99.9) | 0.533 |
| **Maternal BMI exposure factors** | | | | | | | | |
| Number of maternal BMI categories | 92.2 (83.6, 97.1) | 0.093 | 91.7 (83.5, 97.5) | 0.542 | 89.3 (69.0, 97.6) | 0.161 | 99.9 (99.8, 99.9) | 0.540 |
| Maternal BMI measured or self-reported | 89.5 (80.1, 96.3) | 0.949 | 91.9 (84.7, 97.2) | 0.819 | 90.8 (75.3, 98.0) | 0.793 | 99.8 (99.7, 99.9) | 0.124 |
| **Child BMI outcome factors** | | | | | | | | |
| Child BMI measured or self-reported | 91.8 (83.6, 97.3) | 0.276 | ^b^ | ^b^ | ^b^ | ^b^ | ^b^ | ^b^ |
| BMI or z-score | 92.1 (84.8, 97.2) | 0.934 | 89.4 (80.0, 96.4) | 0.101 | 90.0 (71.9, 98.0) | 0.538 | 99.9 (99.8, 99.9) | 0.934 |
| Child Age | **81.2 (61.7, 93.6)** ^a^ | **<0.0001**^a^ | **83.2 (67.3, 94.8)** ^a^ | **0.0002**^a^ | 83.0 (52.2, 97.1) | 0.065 | 99.9 (99.8, 99.9) | 0.304 |
| Child ages combined | 92.1 (84.7, 97.3) | 0.396 | 91.6 (84.5, 97.1) | 0.813 | 92.9 (80.6, 98.6) | 0.284 | 99.9 (99.8,99.9) | 0.613 |
| Number of cases | 88.6 (78.4, 96.1) | 0.142 | 89.5 (80.1, 96.4) | 0.165 | 88.9 (70.3, 97.6) | 0.736 | N/A | N/A |
| Single/ combined child age | 93.0 (86.6, 97.7) | 0.831 | 91.6 (84.3, 97.1) | 0.814 | 93.0 (80.5, 98.5) | 0.286 | 99.9 (99.8, 99.95) | 0.613 |
| **Adjustments** | | | | | | | | |
| Adjusted | 90.8 (81.9, 96.8) | 0.119 | 90.8 (83.0, 96.8) | 0.884 | 93.9 (82.8, 98.8) | 0.910 | 99.9 (99.8, 99.9) | <0.0001 |
| Adjusted for GWG | 91.6 (83.8,97.1) | 0.338 | 88.5 (78.6, 96.0) | 0.119 | 88.2 (68.3, 97.5) | 0.538 | ^b^ | ^b^ |
| Adjusted for GDM | 88.1 (77.0, 96.0) | 0.291 | 90.9 (82.7, 96.8) | 0.898 | 88.2 (68.3, 97.5) | 0.538 | 99.9 (99.8, 99.9) | 0.423 |
| Adjusted for additional maternal co-morbidities | 92.5 (85.5, 97.4) | 0.631 | 92.7 (86.1, 97.4) | 0.636 | 93.1 (80.4, 98.5) | 0.871 | **99.8 (99.7, 99.9)** ^a^ | **0.009**^a^ |
| Adjusted for maternal age | 92.3 (84.9, 97.1) | 0.351 | 91.3 (83.8, 97.0) | 0.903 | 91.6 (77.0, 98.2) | 0.479 | 99.9 (99.8, 99.95) | 0.751 |
| Adjusted for parity | 91.8 (84.1, 97.0) | 0.658 | 91.6 (84.3, 97.3) | 0.365 | 86.9 (62.7, 97.6) | 0.058 | 99.9 (99.8, 99.9) | 0.680 |
| Adjusted for birth weight | 91.9 (84.3, 97.0) | 0.719 | 89.2 (79.3, 96.4) | 0.241 | 89.8 (71.8, 98.0) | 0.224 | 99.9 (99.8, 99.95) | 0.344 |
| Adjusted for gestational age at birth | 88.2 (76.8, 95.9) | 0.493 | 90.5 (82.2, 96.7) | 0.893 | 85.7 (58.9, 97.2) | 0.200 | 99.8 (99.7, 99.9) | 0.474 |
| Adjusted for offspring sex^c^ | 92.9 (86.1, 97.7) | 0.991 | 92.2 (84.9, 97.7) | 0.502 | 91.5 (72.6, 99.0) | 0.457 | 99.9 (99.8, 99.9) | 0.435 |
| Adjusted for ethnicity | 92.2 (84.8, 97.3) | 0.982 | 92.6 (86.1, 97.6) | 0.812 | 88.6 (66.1, 98.4) | 0.175 | 99.9 (99.8, 99.95) | 0.592 |
| Adjusted for breast/infant feeding | 91.3 (83.2, 96.7) | 0.509 | 91.3 (83.7, 96.9) | 0.875 | 92.0 (78.2, 98.3) | 0.574 | 99.9 (99.8, 99.9) | 0.471 |
| Adjusted for paternal BMI | 93.0 (86.4, 97.4) | 0.553 | 92.9 (86.6, 97.6) | 0.636 | 93.9 (82.8, 98.8) | 0.911 | 99.9 (99.8, 99.9) | 0.635 |
| Adjusted for child lifestyle | ^b^ | ^b^ | 93.0 (86.8, 97.6) | 0.532 | ^b^ | ^b^ | 99.9 (99.8, 99.9) | 0.724 |
| Adjusted for maternal smoking | 92.5 (85.6, 97.4) | 0.823 | 91.4 (83.9, 97.0) | 0.866 | 91.6 (77.0, 98.2) | 0.480 | **99.9 (99.8, 99.9)** ^a^ | **0.036**^a^ |
| Adjusted for SES measure | 90.8 (82.1, 96.8) | 0.721 | 92.2 (85.1, 97.3) | 0.876 | 92.0 (78.3, 98.3) | 0.574 | 99.9 (99.8, 99.9) | 0.631 |
| Adjusted for parental weight at time of child BMI measure | ^b^ | ^b^ | 91.7 (84.3, 97.3) | 0.069 | ^b^ | ^b^ | ^b^ | ^b^ |
| **Combined factors resulting in significant reduction in heterogeneity** | | | | | | | | |
| Child age + continent | **62.6 (25.4,89.8)** | <0.001 | **72.9 (47.6,95.3)** ^a^ | <0.001 | **25.4 (0.00,96.6)** | <0.001 | NA | NA |

Abbreviations: GWG, gestational weight gain; GDM, gestational diabetes mellitus; SES, socio-economic status; BMI, body mass index; NA, not applicable; I^2^, I^2^ statistic.

Footnote:

^a^Bold data show a significant reduction in heterogeneity.

^b^No variability was found between studies for this variable.

^c^The majority of included studies used sex-specific BMI criteria to define child weight status

**^d^**p-value associated with whether the variable is a source of heterogeneity
