# Supplementary material for: The association between maternal body mass index and child obesity: A systematic review and meta-analysis
Source: PLoS Med. 2019 Jun 11;16(6):e1002817. doi: 10.1371/journal.pmed.1002817 (PMC6559702; doi:10.1371/journal.pmed.1002817)
Supplement: S19 Table — (DOCX) [file pmed.1002817.s029.docx]

# S19 Table: Subgroup meta-analysis: Odds of childhood weight status per 5-kg/m^2^ increase in maternal BMI, according to the continent of study

|  | **Child Obesity (≥95^th^ percentile)**  **OR (95% CI)** | **Child overweight or obesity (≥85^th^ percentile)**  **OR (95% CI)** | **Child overweight (85^th^ to 95^th^ percentile)**  **OR (95% CI)** |
| --- | --- | --- | --- |
| **Asia** | 1.32 (1.27, 1.36) | 1.30 (1.23, 1.37) | 1.16 (1.13, 1.20) |
| **Australia** | N/A | 1.41 (1.03, 1.93) | N/A |
| **Europe** | 2.27 (1.95, 2.64) | 1.84 (1.64, 2.06) | 1.73 (1.63, 1.83) |
| **North America** | 1.62 (1.49, 1.75) | 1.42 (1.30, 1.54) | 1.17 (1.04, 1.32) |
| **South America** | 2.11 (1.57, 2.82) | 1.44 (1.23, 1.70) | N/A |

Abbreviations: OR, odds ratio; CI, confidence interval; N/A, not applicable as too few studies to carry out sub-group meta-analysis.
