# Supplementary material for: The association between maternal body mass index and child obesity: A systematic review and meta-analysis
Source: PLoS Med. 2019 Jun 11;16(6):e1002817. doi: 10.1371/journal.pmed.1002817 (PMC6559702; doi:10.1371/journal.pmed.1002817)
Supplement: S1 Text — (DOCX) [file pmed.1002817.s030.docx]

# S1 Text: Dose-response meta-analysis methods

The dose response meta-analysis was performed as suggested by Crippa & Orsini [1] in a two stage analysis.

In the first stage the aim was to estimate the dose-response association between the adjusted log relative risks and the levels of a specific exposure in a particular study. The model consisted of a linear regression model where the dependent variable was a vector of log odds ratios (not including the reference one), and the independent variable represented the corresponding dose (linear analysis) or a spline transformation of the dose (non-linear analysis). The model has no intercept because the log odds ratio is equal to zero for the reference group.

However, the error terms in dose-response data are not independent as they are constructed using a common reference group and Greenland & Longnecker [2] and Orsini *et al.* [3] showed that assuming zero covariance or correlation leads to biased estimates of the trend. Therefore, the Greenland & Longnecker [2] method was used to approximate the covariances and efficiently estimate the vector of regression coefficients of the model using the generalized least squares method.

Linear tail-restricted cubic splines with two spline transformations and three knots at the 5th, 50th and 95th percentiles of the aggregated exposure distribution were used to transform the data in the non-linear analysis. Nonlinearity was assessed by testing that the coefficient of the second spline was equal to zero [4], which requires ORs and CIs to be available for at least three maternal BMI categories.

The aim of the second stage of the analysis was to combine study-specific estimates using established methods for multivariate meta-analysis, also described in detail in Crippa & Orsini [1].

**References:**

1. Crippa A, Orsini N. Multivariate Dose-Response Meta-Analysis: The dosresmeta R Package. 2016. 2016;72(Code Snippet 1):15.

2. Greenland S, Longnecker MP. Methods for Trend Estimation from Summarized Dose-Response Data, with Applications to Meta-Analysis. Am J Epidemiol. 1992;135(11):1301-9.

3. Orsini N, Li R, Wolk A, Khudyakov P, Spiegelman D. Meta-analysis for linear and nonlinear dose-response relations: examples, an evaluation of approximations, and software. Am J Epidemiol. 2012;175(1):66-73.

4. Desquilbet L, Mariotti F. Dose-response analyses using restricted cubic spline functions in public health research. Statistics in medicine. 2010;29(9):1037-57.
